# Triterpenoid Saponins and Flavonoid Glycosides from the Flower of *Camellia flavida* and Their Cytotoxic and α-Glycosidase Inhibitory Activities

**DOI:** 10.3390/ijms252010977

**Published:** 2024-10-12

**Authors:** Siyuan Ma, Yuxin Wu, Hanfeng Min, Li Ge, Kedi Yang

**Affiliations:** Medical School of Guangxi University, Nanning 530004, China2228391049@st.gxu.edu.cn (Y.W.); 2328302011@st.gxu.edu.cn (H.M.)

**Keywords:** *Camellia flavida*, functional food, triterpenoid saponins, flavonoid glycosides, cytotoxicity, α-glycosidase inhibitory activity

## Abstract

*Camellia flavida* var. flavida, commonly known as “Jinhua Tea”, has its flowers and leaves traditionally utilized as tea and functional food sources. However, there is limited knowledge about its bioactive components and their biological activities. This study isolated ten previously unidentified glycoside compounds from the flowers of *Camellia flavida*, including three oleanane-type triterpenoid saponins (compounds **1**–**3**) and seven flavonoid glycosides (compounds **4**–**10**), collectively named flavidosides A–J. This study assessed the cytotoxicity of these compounds against a panel of human cancer cell lines and their α-glucosidase inhibitory activities. Notably, flavidoside C showed significant cytotoxicity against BEL-7402 and MCF-7 cell lines, with IC_50_ values of 4.94 ± 0.41 and 1.65 ± 0.39 μM, respectively. Flavidoside H exhibited potent α-glucosidase inhibitory activity, with an IC_50_ value of 1.17 ± 0.30 mM. These findings underscore the potential of *Camellia flavida* in the development of functional foods.

## 1. Introduction

Yellow camellia, belonging to the Theaceae family and referred to as “Jinhua Tea” in China, was designated as a new food source by the State Food and Drug Administration of China in 2010 and was approved as a food ingredient in 2023. Numerous studies have demonstrated its pharmacological benefits, including antibacterial activity [1], potential hepatitis treatment [2], blood sugar reduction [3], antioxidant effects [4], and cancer prevention capabilities [5]. *Camellia flavida* var. flavida, a distinguished species of yellow camellia, was identified in Chongzuo, Guangxi, in 1982. This species is characterized by its year-round blooming and substantially higher flower yield compared to other species. Due to its high commercial value and potential as a tea and functional food source, its cultivation has expanded significantly in recent years.

While recent studies by our group have focused on the chemical and biological properties of *C. flavida* leaves [6,7], research on its flowers has not yet been conducted. Consequently, this study investigates the bioactive constituents and biological activities of *C. flavida* flowers. Preliminary extraction studies revealed that *C. flavida* exhibited cytotoxic effects against tumor cells. A substantial body of evidence demonstrates that extracts of *C. flavida* exert inhibitory actions on non-small cell lung cancer [8], hepatocellular carcinoma [9], and colon cancer [10]. However, these studies predominantly employ crude extracts of *C. flavida*, whereas many modern chemotherapeutic agents are derived from single, purified natural compounds, such as paclitaxel, vincristine, and onychomycotoxin analogs [11]. Consequently, there is a compelling need to isolate and evaluate individual compounds from *C. flavida* for their antitumor potential; we assessed the cytotoxic activities of individual compounds against cancer cells lines using the MTT assay.

In traditional Chinese medicine, yellow camellia has long been consumed as a health beverage believed to lower blood sugar levels. Notably, the flavonoids of *Camellia nitidissima* flower extracts have demonstrated in vitro α-glucosidase inhibitory activity with an IC_50_ value of 45 μg/mL [12]. The activity of α-glucosidase plays a pivotal role in modulating carbohydrate utilization and absorption in the small intestine. The inhibition of α-glucosidase can effectively diminish carbohydrate digestion and absorption, leading to a notable reduction in postprandial blood glucose levels. This, in turn, aids in the management of metabolic disorders, including diabetes and obesity [13]. Although clinical medications, such as acarbose and voglibose, are available for diabetic patients, they are often associated with and accompanied by undesirable side effects, encompassing flatulence, diarrhea, and abdominal discomfort. As a result, numerous reports have underscored the efficacy of safe, natural phytochemical enzyme inhibitors derived from sources like mulberry leaves, guava fruits, and oil tea seeds in mitigating postprandial hyperglycemia. These findings indicate the potential of *C. flavida* as a functional food component to control blood glucose and alleviate symptoms of diabetes mellitus.

**Figure 1:**
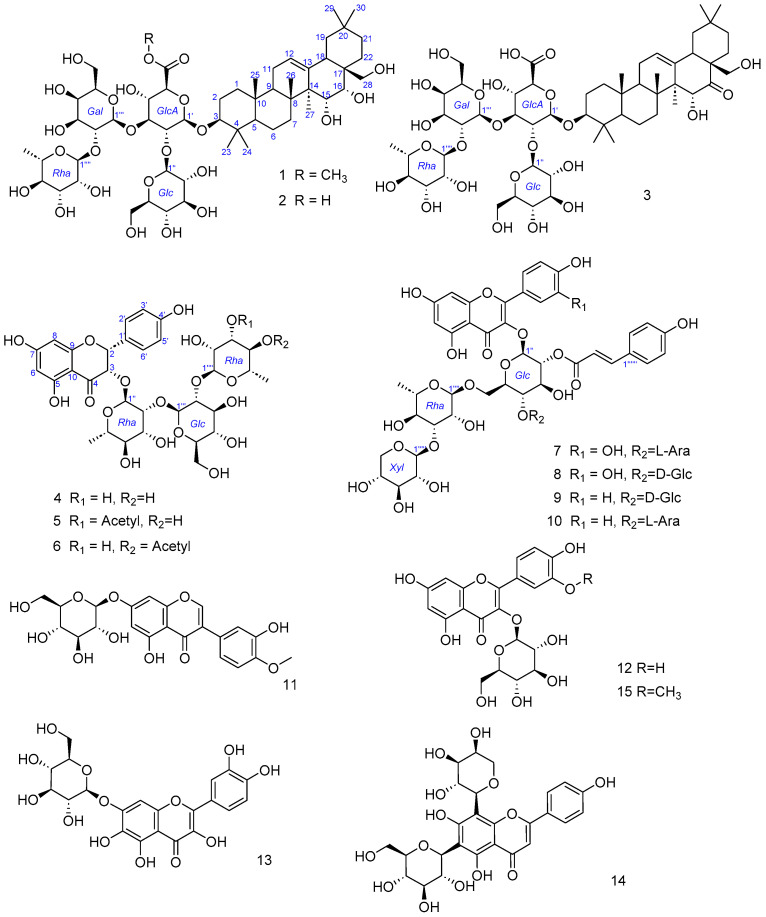
Structure of compounds **1**–**15** from the flower of *C. flavida*.

In this research, ten new compounds (Figure 1), including three triterpenoid saponins (**1**–**3**) and seven flavonoid glycosides (**4**–**10**), along with five previously identified flavonoids (**11**–**15**), were isolated from *C. flavida*. The cytotoxic activities of compounds **1**–**10** were assessed against six cancer cell lines using the MTT assay, and their α-glucosidase inhibitory activities were also evaluated.

## 2. Results and Discussion

**Figure 2:**
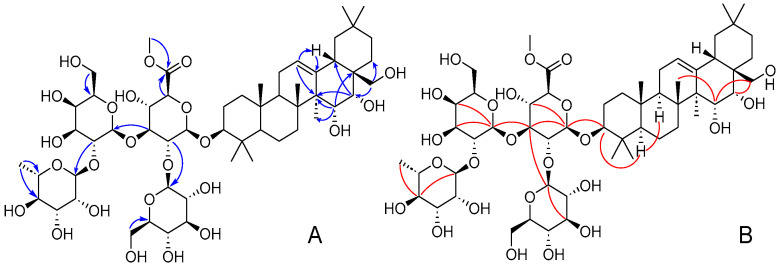
Key HMBC (**A**) and NOESY (**B**) correlations of compound **1**.

Compound **1** was isolated as a white powder, and its molecular formula was determined as C_55_H_90_O_24_ based on HR-ESI-MS analysis (Figure S1, *m*/*z* 1157.5726, [M + Na]^+^, calcd 1157.5720). The Liebermann–Burchard reaction for compound **1** was positive, indicating its identity as a potential triterpenoid. NMR analysis (Table 1 and Table 2, and Figures S2–S10) revealed seven singlet methyl groups at chemical shifts *δ*_H_ 0.82, 1.02, 1.05 × 2, 1.10, 1.12, and 1.81 in the up-field region, and *δ*_H_ 2.45 (dd, *J* = 13.5, 3.8 Hz, H-18), a notable proton signal with two characteristic unsaturated carbons at *δ*_C_ 146.0 (C-13) and *δ*_C_ 123.9 (C-12), indicating an olean-12-ene pentacyclic triterpenoid skeleton. Additionally, three oxymethine protons were observed at *δ*_H_ 3.17, 4.48, and 4.55, corresponding to carbons C-3 (*δ*_C_ 89.7), C-16 (*δ*_C_ 78.7), and C-15 (*δ*_C_ 67.4), respectively. Two methylene protons attached to C-28 (*δ*_C_ 69.6) appeared as a doublet at *δ*_H_ 3.60 and 3.79 (both d, *J* = 10.5 Hz). Moreover, HMBC correlations (Figure 2A) indicated three hydroxy groups linked to C-15, C-16, and C-28, demonstrated by HMBC cross-peaks from *δ*_H_ 4.48 (H-15) to *δ*_C_ 48.0 (C-14), 41.1 (C-17), and 21.0 (C-27), from *δ*_H_ 4.55 (H-16) to *δ*_C_ 48.0 (C-14) and 41.1 (C-17), and from *δ*_H_ 3.60/3.79 (H_2_-28) to *δ*_C_ 41.1 (C-17) and 31.0 (C-22). The α-orientation of H-3 was confirmed by the *trans*-diaxial coupling constant of a large ^3^*J* value (10.2 Hz) and NOESY correlations (Figure 2B) of *δ*_H_ 3.17 (H-3)/0.77 (H-5). The *cis*-orientations of H-15/H-16 were suggested by a small ^3^*J* value of H-16 (3.8 Hz), and NOESY correlations of *δ*_H_ 4.48 (H-15)/1.05 (H_3_-26)/3.60 (H_2_-28), *δ*_H_ 4.55 (H-16)/3.60 (H_2_-28), and *δ*_H_ 3.60 (H_2_-28)/2.45 (H-18)/1.05 (H_3_-29) established that H-15 and H-16 were β-oriented. Thus, the aglycone part of compound **1** was established as a 3,15,16,28-tetrahydroxyolean-12-ene framework. Moreover, the NMR characteristics were found to be very similar to those of camellianol F [14].

Anomeric proton signals at *δ*_H_ 4.86, 5.87, 6.10, and 6.23 were observed and displayed HSQC correlations with the anomeric carbon signals at *δ*_C_ 105.2, 102.9, 101.6, and 102.3, respectively, characteristic of the four monosaccharide units. The ^13^C-NMR signals of the four sugars were determined from the HMBC and TOCSY spectra. Specifically, *δ*_C_ 105.2, 79.5, 82.8, 70.9, 76.6, and 170.3 were assigned to glucopyranuronic acid. *δ*_C_ 102.9, 76.4, 78.5, 72.6, 78.2, and 63.5 were associated with glucopyranose. *δ*_C_ 101.6, 76.2, 76.1, 71.1, 77.0, and 62.0 were identified as galactopyranose, and *δ*_C_ 102.3, 72.6, 72.6, 73.9, 69.8, and 18.3 were attributed to rhamnopyranose. Additionally, acid hydrolysis of **1** yielded D-glucuronic acid, L-rhamnose, D-glucose, and D-galactopy, analyzed by HPLC.

Further analysis of the HMBC correlations of *δ*_H_ 3.69 (GlcA-6-OCH_3_) and 4.45 (GlcA-H-5) to *δ*_C_ 170.3 (GlcA-C-6) indicated a methyl ester group was present at GlcA-C-5. The β-configuration of glucopyranosyl and galactopyranosyl units was confirmed by the relatively large coupling constants (*J* = 7.4 and 7.7 Hz) of their anomeric protons. The broad singlet of its anomeric proton confirmed the α-configuration of the rhamnopyranosyl unit. The β-configuration of the 6-methyl glucopyranuronoyl unit was indicated by the NOESY correlations of *δ*_H_ 4.86 (GlcA-H-1)/4.67 (GlcA-H-3)/4.45 (GlcA-H-5).

The HMBC correlations from Gal-H-1 (*δ*_H_ 6.10) to GlcA-C-3 (*δ*_C_ 70.9) and from Glc-H-1 (*δ*_H_ 5.87) to GlcA-C-2 (*δ*_C_ 79.5) suggested that the GlcA was linked to Gal-C-1 and Glc-C-1 via GlcA-C-2 and GlcA-C-3, respectively. The rhamnose was connected to Gal-C-2, as evidenced by the HMBC correlation from Rha-H-1 (*δ*_H_ 6.23) to Gal-C-2 (*δ*_C_ 76.2). The HMBC correlation from GlcA-H-1 (*δ*_H_ 4.86) to C-3 (*δ*_C_ 89.7) of the aglycone unit confirmed that the tetrasaccharide moiety was located at C-3 of the triterpene skeleton. Therefore, the structure of **1** was established as 3β,15α,16α,28-tetrahydroxyolean-12-en-3-O-β-D-glucopyranosyl-(1→2)-[α-L-rhamnopyranosyl-(1→2)-β-D-galactopyranosyl-(1→3)]-β-D-glucuronopyranoside methyl ester, assigned the trivial name flavidoside A.

Compound **2** was obtained as a white powder and displayed a molecular formula of C_54_H_88_O_24_ by HR-ESI-MS (Figure S11, *m*/*z* 1143.5554, [M + Na]^+^, calcd 1143.5564). The NMR data (Table 1 and Table 2, and Figures S12–S19) for **2** were very similar to **1**. Compounds **1** and **2** shared the same glycoside moiety, specifically camellianol F. However, they differ in the type of sugar moiety that was attached to this common glycoside. The HSQC correlations from *δ*_H_ 4.86 to *δ*_C_ 105.4, from *δ*_H_ 5.90 to *δ*_C_ 102.7, from *δ*_H_ 6.18 to *δ*_C_ 101.4, and from *δ*_H_ 6.23 to *δ*_C_ 102.4 suggested the presence of four sugar units. According to other NMR spectra and acid hydrolysis followed by HPLC analysis, compound **2** comprised β-D-glucopyranuronic acid (GlcA), β-D-glucopyranosyl (Glc), α-L-rhamnopyranosyl (Rha), and β-D-galactopyranosyl (Gal) sugar units. GlcA was linked to Gal-C-1 and Glc-C-1 via GlcA-C-2 and C-3, respectively, and rhamnose was connected to Gal-C-2, as evidenced by the HMBC correlations (Figure S20) from *δ*_H_ 6.18 (Gal-H-1) to *δ*_C_ 82.8 (GlcA-C-3), from *δ*_H_ 5.90 (Glc-H-1) to *δ*_C_ 79.5 (GlcA-C-2), and from *δ*_H_ 6.23 (Rha-H-1) to *δ*_C_ 76.4 (Gal-C-2). Thus, the structure of compound **2** was established as 3β,15α,16α,28-tetrahydroxyolean-12-en-3-O-β-D-glucopyranosyl-(1→2)-[α-L-rhamnopyranosyl-(1→2)-β-D-galactopyranosyl-(1→3)]-β-D-glucuronopyranoside (flavidoside B).

Compound **3** was isolated as a white powder. The molecular formula was deduced as C_54_H_86_O_24_ by HR-ESI-MS (Figure S21, *m*/*z* 1141.5389 [M + Na]^+^, calcd 1141.5407). The ^1^H and ^13^C NMR data were very similar to those of compound 2, except for the absence of the hydroxy group at C-16, which was replaced by a carbonyl group in the triterpene skeleton. The sugar chain was identical in compounds **3** and **2**, as confirmed by the NMR data (Table 1 and Table 2, and Figures S22–S31). As a result, compound **3** was elucidated as 3β,15α,28-trihydroxyolean-12-en-16-on-3-O-β-D-glucopyranosyl-(1→2)-[α-L-rhamnopyranosyl-(1→2)-β-D-galactopyranosyl-(1→3)]-β-D-glucuronopyranoside (flavidoside C).

Compound **4** was isolated as a yellow amorphous powder. The molecular formula was determined to be C_33_H_42_O_19_ from its positive HR-ESI-MS (Figure S32, *m*/*z* 765.2207 [M + Na]^+^, calcd 765.2218). *δ*_H_ 5.92 (H-6) and 5.90 (H-8) in the ^1^H-NMR spectrum (Table 3 and Figures S33–S40) displayed a pair of doublets (*J* = 1.9 Hz) representing aromatic protons. The aromatic proton signals at *δ*_H_ 6.85 (2H, d, *J* = 8.4 Hz) and 7.36 (2H, d, *J* = 8.4 Hz) of the A_2_B_2_ type suggested a 1,4-disubstituted B-ring. Two oxygenated methines were observed at *δ*_H_ 4.55 and 5.48 (both d, *J* = 10.4 Hz). These findings indicated that compound **4** was a 3,5,7,4′-tetrahydroxyflavanone (dihydrokaempferol) [15].

Compound **4** contained three sugar moieties, deduced from the anomeric signals of *δ*_H/C_ 4.71/102.1, 4.02/105.3, and 5.21/101.8. The ^13^C-NMR, HMBC, and TOCSY spectra displayed the signal attribution of three sugars: *δ*_C_ 102.1, 82.4, 71.6, 73.9, and 70.9 were assigned to one rhamnopyranose; *δ*_C_ 101.8, 71.9, 71.8, 74.2, 70.3, and 17.9 were associated with another rhamnopyranose; *δ*_C_ 105.3, 78.9, 78.3, 70.9, 77.2, and 62.1 were identified as the glucopyranose. Moreover, the configurations of L-rhamnose and D-glucose were determined by acid hydrolysis and HPLC identification. The anomeric proton at *δ*_H_ 4.02 (d, *J* = 7.6 Hz) suggested the β-configuration of glucopyranosyl. The two α-configuration units of rhamnopyranosyl were determined by the broad singlet of their anomeric protons. Thus, compound **4** was identified as a 3,5,7,4′-tetrahydroxyflavanone substituted with two α-L-rhamnopyranosyl moieties and a β-D-glucopyranosyl moiety.

**Figure 3:**
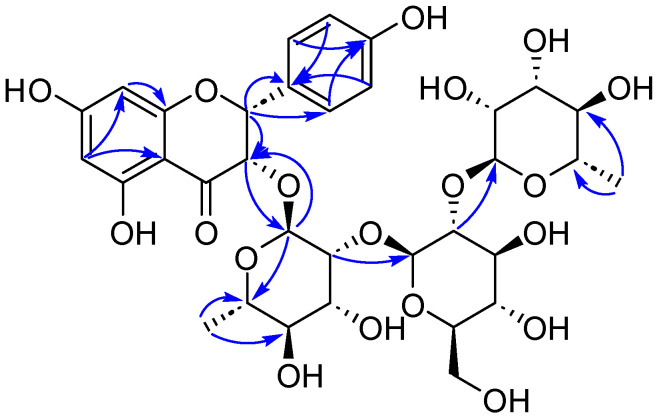
Key HMBC correlations of compound **4**.

The linkage of the rhamnopyranosyl unit at C-2 of the glucopyranosyl unit and the glucopyranosyl moiety was attached to C-2 of another rhamnopyranosyl moiety, confirmed by the HMBC correlations (Figure 3) from *δ*_H_ 5.21 (Rha2-H-1) to *δ*_C_ 78.9 (Glc-C-2) and from *δ*_H_ 4.02 (Glc-H-1) to *δ*_C_ 82.4 (Rha1-C-2). Moreover, the oligosaccharide chain was linked at C-3 of the aglycone, also confirmed by the HMBC correlation from *δ*_H_ 4.71 (Rha1-H-1) to *δ*_C_ 81.2 (C-3) and the reverse correlation from *δ*_H_ 4.55 (H-3) to Rha1-C-1 (*δ*_C_ 102.1).

**Figure 4:**
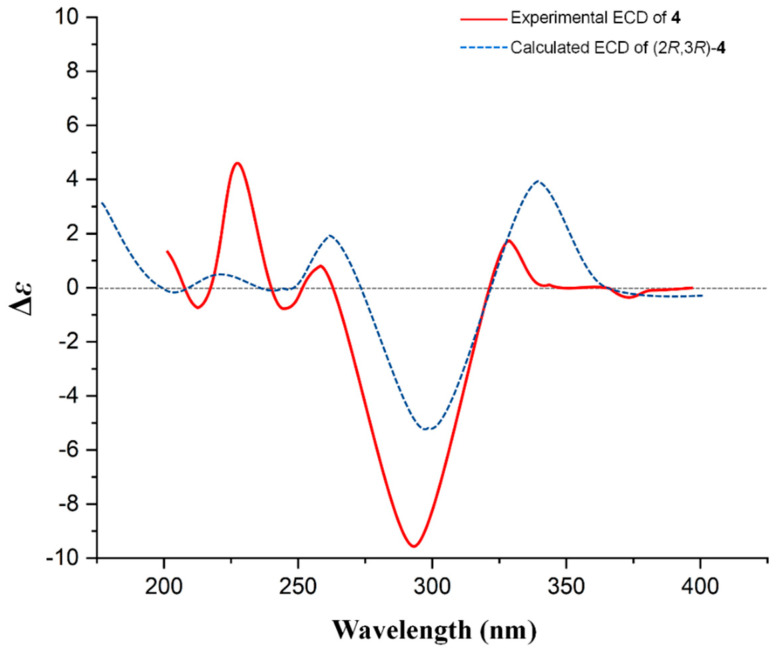
The experimental ECD of **4** and calculated ECD of (2*R*, 3*R*)-**4**.

The large ^3^*J*_H-2/H-3_ value (10.4 Hz) hinted at the *trans*-form orientation of H-2 and H-3. The ECD spectrum of 4 (Figure 4) showed a positive Cotton effect at 330 nm (Δ*ε* +2.19) and a strong negative Cotton effect at 292 nm (Δ*ε* −9.50), establishing that the aglycone of **4** was 2*R* and 3*R* [16]; this was confirmed by the calculated ECD of **4** as well. Based on the above analysis, compound 4 could be assigned as (2*R*,3*R*)-3,5,7,4′-tetrahydroxyflavanone-3-O-α-L-rhamnopyranosyl-(1→2)-β-D-glucopyranosyl-(1→2)-α-L-rhamnoside (flavidoside D).

**Figure 5:**
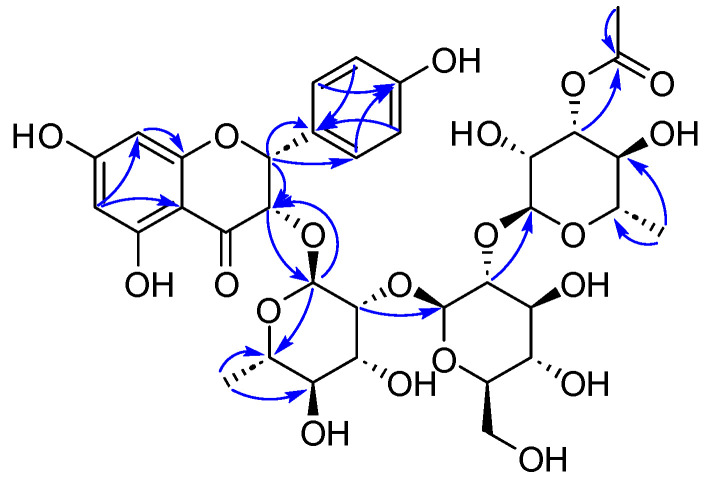
Key HMBC correlations of compound **5**.

**Figure 6:**
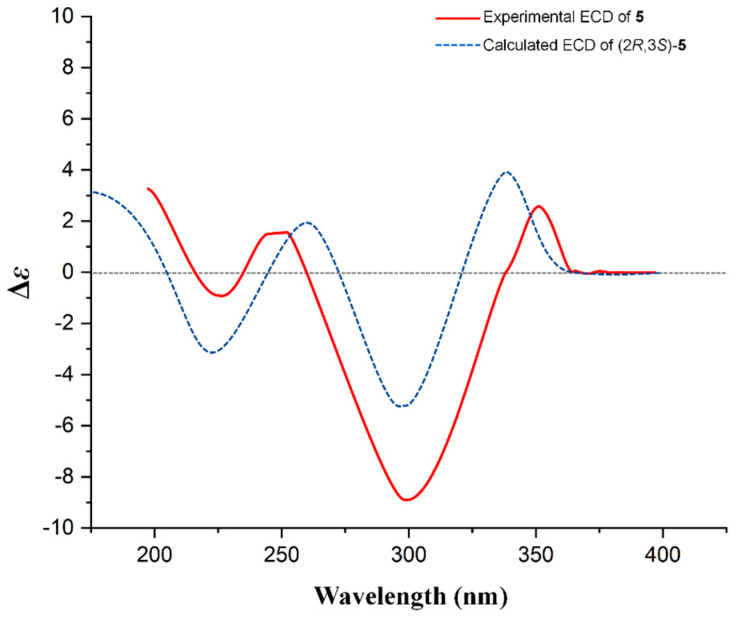
The experimental ECD of **5** and calculated ECD of (2*R*, 3*S*)-**5**.

Compound **5** was isolated as a yellow powder with the molecular formula determined to be C_35_H_44_O_20_ using HR-ESI-MS data (Figure S41, *m*/*z* 807.2327, [M + Na]^+^, calcd 807.2324). The ^1^H and ^13^C NMR data (Table 4, Figures S42–S49) for **5** were similar to **4**, with an added acetyl moiety at position Rha2-C-3, suggested by the HMBC correlations (Figure 5) from *δ*_H_ 5.03 (Rha2-H-3) and 2.08 (acetyl-H-2) to *δ*_C_ 172.8 (acetyl-C-1). The small ^3^*J*_H-2/H-3_ value (2.3 Hz) hinted at the *cis*-form orientation of H-2/H-3. The absolute configuration of 2*R*, 3*S* was confirmed by comparing the experimental ECD and calculated ECD data of **5** (Figure 6). Thus, compound 5 was assigned as (2R,3S)-3,5,7,4′-tetrahydroxyflavanone-3-O-[3-acetyl-O-α-L-rhamnopyranosyl-(1→2)-β-D-glucopyranosyl-(1→2)]-α-L-rhamnoside (flavidoside E).

Compound **6** was obtained as a yellow solid, with the same molecular formula as **5** (C_35_H_44_O_20_) deduced by HR-ESI-MS (Figure S50, *m*/*z* 807.2311 [M + Na]^+^, calcd 807.2324). The ^1^H and ^13^C NMR data (Table 4, Figures S51–S57) were very similar to compound **5** except for the position of the acetyl unit. The acetyl unit linked to C-4 of the rhamnopyranosyl moiety was confirmed by the HMBC correlation (Figure S58) from *δ*_H_ 4.72 (Rha2-H-4) to *δ*_C_ 170.2 (acetyl-C-1). The large ^3^*J*_H-2/H-3_ value (8.4 Hz) revealed the trans-form orientation of H-2/H-3 and the absolute configuration of 2*R*,3*R*, which was displayed by the ECD spectra (Figure S59). Thus, compound **6** was identified as (2*R*,3*R*)-3,5,7,4′-tetrahydroxyflavanone-3-O-[4-acetyl-O-α-L-rhamnopyranosyl-(1→2)-β-D-glucopyranosyl-(1→2)]-α-L-rhamnoside (flavidoside F).

**Figure 7:**
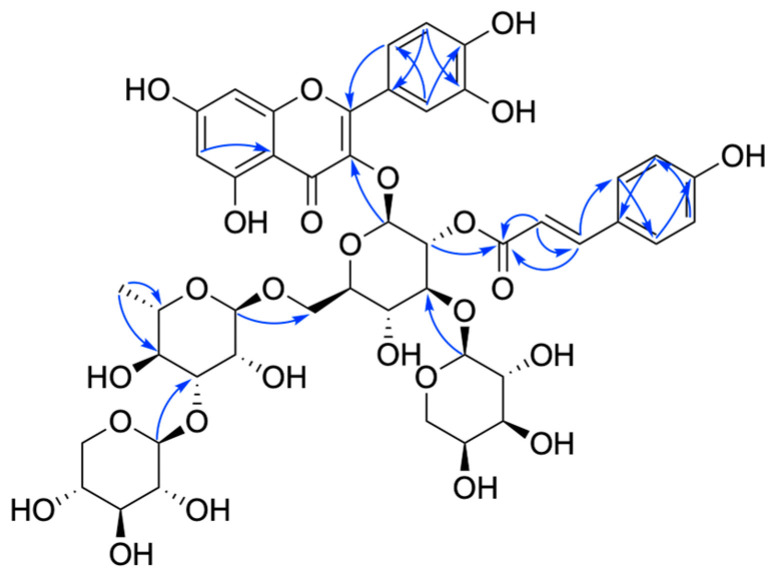
Key HMBC correlations of compound **7**.

Compound **7** was a yellow powder and displayed the molecular formula of C_46_H_52_O_26_ (Figure S60, *m*/*z* 1021.2837, [M + H]^+^, calcd 1021.2825) based on the HR-ESI-MS data. The presence of 3,5,7,3′,4′-pentahydroxyflavone aglycone (quercetin) [17] was confirmed by a pair of aromatic proton signals at *δ*_H_ 6.13 and 6.32 (both br s) along with 6.88 (d, *J* = 7.8 Hz), 7.55 (br d, *J* = 7.8 Hz), and 7.60 (1H, br s), as well as the HMBC correlations (Figure 7) from *δ*_H_ 7.60 (H-2′) and 7.55 (H-6′) to *δ*_C_ 158.9 (C-2) in the ^1^H-NMR spectrum (Table 5).

The 1D and 2D NMR spectra (Figures S61–S67) of **7** suggested that it contains four sugar units: glucopyranose (*δ*_C_ 100.7, 74.5, 84.5, 70.3, 76.8, 68.4), arabinopyranose (*δ*_C_ 105.2, 72.2, 73.9, 69.5, 67.2), rhamnopyranose (*δ*_C_ 102.2, 71.6, 82.4, 72.7, 69.5, 17.9), and xylopyranose (*δ*_C_ 106.2, 75.3, 77.5, 71.1, 66.8). Acid hydrolysis and HPLC confirmed the configuration of these sugars.

HMBC correlations were observed from *δ*_H_ 3.88 (Glc-H-3) to *δ*_C_ 105.2 (Ara-C-1) and from *δ*_H_ 3.51 (Glc-H-6) to *δ*_C_ 102.2 (Rha-C-1), establishing that Glc is linked to Ara-C-1 and Rha-C-1 through Glc-C-2 and Glc-C-6, respectively. The xylopyranoyl unit was linked to Rha-C-3, as shown by the HMBC correlation from *δ*_H_ 4.39 (Xyl-H-1) to *δ*_C_ 82.4 (Rha-C-3). The sugar chain was positioned at C-3 of the flavone, confirmed by the HMBC correlation from *δ*_H_ 5.58 (Glc-H-1) to *δ*_C_ 134.8 (C-3). Signals for a 1,4-disubstituted benzene ring proton at *δ*_H_ 6.80 (2H, Coumaroyl-H-2 and 6) and 7.45 (2H, Coumaroyl-H-3 and 5), and for *trans*-oriented vinylic protons at *δ*_H_ 6.42 (Coumaroyl-H-8) and 7.68 (Coumaroyl-H-7), were noted. Moreover, HMBC correlations from *δ*_H_ 6.42 (Coumaroyl-H-8) to *δ*_C_ 127.3 (Coumaroyl-C-1), 168.8 (Coumaroyl-C-9), from *δ*_H_ 7.68 (Coumaroyl-H-7) to *δ*_C_ 131.3 (Coumaroyl-C-2 and Coumaroyl-C-6), and 115.1 (Coumaroyl-C-8) suggested a *p*-*E*-coumaroyl unit. The HMBC correlation from *δ*_H_ 5.24 (Glc-H-2) to *δ*_C_ 168.8 (Coumaroyl-C-9) indicated that coumaroyl was attached to Glc-C-2. Therefore, compound **7** was identified as 3,5,7,3′,4′-pentahydroxyflavonol-3-O-α-L-arabinopyranosyl-(1→3)-[β-D-xylopyranosyl-(1→3)-α-L-rhamnopyranosyl-(1→6)]-2-O-*p*-*E*-coumaryl-β-D-glucopyranoside, named flavidoside G.

Compound **8**, isolated as a yellow solid, had its molecular formula determined as C_47_H_54_O_27_ from the ion peak at *m*/*z* 1073.2747 [M + Na]^+^ (calcd 1073.2750, Figure S68) in the HR-ESI-MS. The ^1^H- NMR spectrum (Table 5) showed that **8** contained the same flavone aglucone structure of quercetin as **7**. The difference between **8** and **7** was in the sugar units. The sugar chain in **8** included two β-D-glucopyranosyl units, a α-L-rhamnopyranosyl unit, and a β-D-xylopyranoyl unit, as suggested by the 1D and 2D NMR data (Figures S69–S75) and acid hydrolysis. HMBC correlations (Figure S76) from *δ*_H_ 4.24 (Xyl-H-1) to *δ*_C_ 81.1 (Rha-C-3), from *δ*_H_ 4.38 (Rha-H-1) to *δ*_C_ 67.9 (Glc1-C-6), from *δ*_H_ 4.31 (Glc2-H-1) to *δ*_C_ 83.1 (Glc1-C-3), from *δ*_H_ 5.09 (Glc1-H-2) to *δ*_C_ 165.7 (Coumaroyl-C-9), and from *δ*_H_ 5.57 (Glc1-H-1) to *δ*_C_ 132.8 (C-3) revealed the positions of the sugars and confirmed that the sugar chain was located at C-3 of the aglucone. Based on this evidence, the structure of 8 was identified as 3,5,7,3′,4′-pentahydroxyflavonol-3-O-β-D-glucopyranosyl-(1→3)-[β-D-xylopyranosyl-(1→3)-α-L-rhamnopyranosyl-(1→6)]-2-*p*-*E*-coumaryl-β-D-glucopyranoside (flavidoside H).

Compound **9**, obtained as a yellow powder, had its molecular formula identified as C_47_H_54_O_26_ by HR-ESI-MS (Figure S77, *m*/*z* 1035.3014, [M + H]^+^, calcd 1035.2981). The ^1^H and ^13^C NMR data (Table 5 and Table 6, and Figures S78–S86) were similar to those of compound **8**. The distinction of **9** was its 1,4-disubstituted B-ring, evidenced by its A_2_B_2_-type aromatic proton signals at *δ*_H_ 6.89 and 7.98 (both d, *J* = 8.3 Hz). The flavone aglucone structure of **9** was kaempferol [18]. Thus, compound **9** was designated as 3,5,7,4′-tetrahydroxyflavonol-3-O-β-D-glucopyranosyl-(1→3)-[β-D-xylopyranosyl-(1→3)-α-L-rhamnopyranosyl-(1→6)]-2-*p*-*E*-coumaryl-β-D-glucopyranoside (flavidoside I).

Compound **10** was obtained as a yellow powder. Its molecular formula was determined to be C_46_H_52_O_25_ (Figure S87, *m*/*z* 1005.2885, [M + H]^+^, calcd 1005.2876) based on HR-ESI-MS. Its ^1^H and ^13^C NMR data (Table 5 and Table 6, and Figures S88–S96) were very similar to those of compound **7**. The difference in **10** was the presence of para substitution on the B-ring. Thus, compound **10** was designated as 3,5,7,4′-tetrahydroxyflavonol-3-O-α-L-arabinopyranosyl-(1→3)-[β-D-xylopyranosyl-(1→3)-α-L-rhamnopyranosyl-(1→6)]-2-*p*-*E*-coumaryl-β-D-glucopyranoside (flavidoside J).

The known compounds **11** to **15** were identified as pratensein-7-O-β-D-glucoside (**11**) [19], quercetagetin-7-O-β-D-glucoside (**12**) [20], quercetin-3-O-β-D-glucoside (**13**) [21] apigenin-8-C-α-L-arabinosyl-6-C-β-D-glucoside (**14**) [22], and 3,4′-,5,7-tetrahydroxy-3′-methoxyflavone-3-O-β-D-glucopyranoside (**15**) [23], respectively (Figures S97–S106).

The cytotoxicity of compounds **1**–**10** was evaluated against six human cancer cell lines and two normal human cell lines using the MTT method. Compounds **1**–**3**, representing newly identified triterpenoid saponins, exhibited inhibitory effects against Hela, MCF-7, BEL-7402, Hep G2, and MDA-MB-231 cell lines. Notably, compound **3** demonstrated potent cytotoxic effects against the MCF-7 and BEL-7402 cell lines, surpassing the positive control with IC_50_ values of 1.65 ± 0.39 and 4.94 ± 0.41 μM, respectively. Moreover, its inhibitory effect on MDA-MB-231 cells was comparable to that of cisplatin. As a novel oleanane-type saponin analog, compound **3** fell within a class of triterpenoid saponins renowned for their potent antitumor properties, positioning it as a promising lead candidate for antitumor drug development [24]. Notably, the IC_50_ value of compound **3** against MCF-7 cells (1.65 ± 0.39 µM) was significantly lower than the reported range (6.1~16.0 µM) for oleanane-type saponins in the study by Huyen, L.T. et al. [25], highlighting its enhanced potency. A thorough literature review on the cytotoxicity of *C. flavida* revealed a lack of prior studies investigating the effects of its active components on BEL-7402 cells. Our findings contribute to bridging this knowledge gap and underscore the potential of compound **3** in the treatment of breast and liver cancer.

Compound **2** demonstrated significantly greater cytostatic effects on Hela cells compared to the positive control, with an IC_50_ value of 4.17 ± 0.85 μM. In contrast, compound **3**, along with compound **1**, exhibited less pronounced inhibitory effects. A review of the literature suggested that the n-butanol and water-soluble fractions of the ethanolic extract from *C*. *flavida* were more potent in inhibiting Hela cell proliferation. Quantitative analysis identifies saponins as the primary constituents in these fractions [26]. Consequently, compound **2** from *C*. *flavida* emerged as a promising lead candidate for cervical cancer treatment.

In contrast, all flavonoid saponins (**4** to **10**) exhibited only weak cytotoxicity against the six cancer cell lines. The triterpenoid saponins exhibited significantly stronger cytotoxicity than the flavonoid glycosides across all six cancer cells. Moreover, none of the compounds displayed cytotoxicity towards normal cells (Table 7).

Despite the cytotoxicity results of compounds **4**–**10** being unsatisfactory, the flavonoid glycosides exhibited strong α-glucosidase inhibitory activity. In contrast, none of the triterpenoid saponins exhibited any inhibitory effect on α-glucosidase. The inhibitory activity of compounds 7 and 10 towards α-glucosidase was equivalent to that of acarbose, exhibiting IC_50_ values of 2.38 ± 0.22 mM and 2.26 ± 0.48 mM, respectively. Compound **9**, on the other hand, demonstrated a moderate level of inhibitory activity against α-glucosidase, with an IC_50_ value of 3.33 ± 0.48 mM. Compound **8** demonstrated strong inhibitory activity against α-glucosidase, with an IC_50_ value of 1.17 ± 0.30 mM, which was superior to that of acarbose (2.04 ± 0.27 mM). The initial analysis of SAR (Structure-Activity Relationship) revealed that the presence of a double bond at positions C-2 and C-3 in the parent nucleus diminished the inhibitory effect on α-glycosidase. Additionally, the number of hydroxyl groups on the skeleton and the type of monosaccharide in the sugar chain had minimal impact on this inhibitory effect. Notably, the introduction of the *p*-*E*-coumaroyl group significantly enhanced the inhibitory effect of the compound against α-glycosidase. Accordingly, we could hypothesize that flavonoid glycosides **4**–**10** derived from the water fraction of *C*. *flavida* constitute a significant basis for its hypoglycemic activity (Table 8).

**Figure 8:**
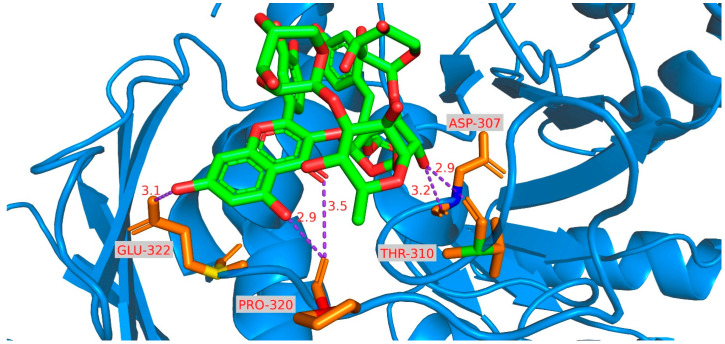
Molecular docking simulations obtained at the lowest energy conformation, highlighting potential hydrogen contacts of **8**.

To explore the reasons behind the inhibitory effects of compounds **1** to **10** on α-glucosidase, molecular analysis docking was conducted to uncover the potential mechanism (Figure 8 and Figures S107–S115). Among these, compound **8** exhibited a binding affinity of −5.48 kcal/mol and effectively occupied the active site pockets, indicating multiple binding interactions. A hydrogen bond was formed between the C-8-OH group and residue A307 at a distance of 2.9 Å. Additionally, two hydrogen bonds were identified between the carbonyl group at C-4 and the C-5-OH group, both with residue A307 at distances of 3.5 Å and 3.1 Å, respectively. The Glc-3-OH group simultaneously formed two hydrogen bonds with residues T310 and A307 at distances of 2.9 Å and 3.2 Å, respectively (Table S1).

## 3. Experimental Procedures

### 3.1. General Experimental Procedures

UV spectra were obtained using a UNICO UV-2802 Spectrometer (Shanghai UNICO Instruments Co., Ltd., Shanghai, China). ECD spectra were measured at room temperature on a Jasco J-815 CD spectrometer (JASCO Corporation, Tokyo, Japan). IR spectra were collected using a Shimadzu IRAffinity-1S FTIR spectrometer (Shimadzu Corporation, Tokyo, Japan). NMR spectra were acquired on Bruker AVANCE III HD 600 MHz spectrometers (Bruker Corporation, Billerica, MA, USA) with Pyr-*d*_5_ (*δ*_H_ 7.20, 7.57, 8.72 and *δ*_C_ 123.44, 135.43, 149.84), CD_3_OD (*δ*_H_ 3.31 and *δ*_C_ 49.00), and DMSO-*d*_6_ (*δ*_H_ 2.50 and *δ*_C_ 39.52) as solvents. HR-ESI-MS data were collected on a Waters G2-XS Q-TOF mass spectrometer (Waters Corporation, Milford, MA, USA) equipped with a BEH-C18 column. Column chromatography was performed using Toyopearl Butyl 650C (Nippon Chemi-Con Corporation, Tokyo, Japan), MCI GEL (CHP20, Mitsubishi Chemical Corporation, Tokyo, Japan), Toyopearl HW-40F (Nippon Chemi-Con Corporation, Tokyo, Japan), macroporous resin of D101-type (Tianjin Berens Biotechnology Co., Ltd., Tianjin, China), and C18 reverse-phase silica gel (SMB 100-20/45, Fuji Silysia Chemical Ltd., Tokyo, Japan). Semipreparative HPLC was conducted with a Welch XB-C18 column (5 μm, 10 × 250 mm, 4 mL/min, Shanghai Welch Technology Co., Ltd., Shanghai, China) on a Laballiance HPLC system (LabAlliance, State College, PA, USA) with a Model 500 UV detector. Analytical HPLC was performed using an SSI HPLC system (Scientific Systems, Inc., State College, PA, USA) with a Welch XB-C18 column (5 μm, 4.6 × 250 mm, 1.0 mL/min) and a Model 201 UV detector. Medium-pressure liquid chromatography was conducted on a Büchi B-688 chromatography pump (BÜCHI Labortechnik Aktiengesellschaft, Flawil, Switzerland) with a C-635 UV photometer.

### 3.2. Plant Material

The flowers of *C. flavida* were authenticated and provided by Guangxi Fangcheng Golden Camellias National Nature Reserve Management Centre in January 2022. A voucher specimen (No. 20220201-8126) of the *C. flavida* plant was deposited in the Medical School of Guangxi University.

### 3.3. Extraction and Isolation

Air-dried flowers of *C. flavida* (2.5 kg) were extracted with 70% EtOH (9 L) for 7 days, yielding a concentrated extract (1 kg). This extract was diluted with water and extracted sequentially with petroleum ether and ethyl acetate. The aqueous layer was filtered and subjected to chromatography on D101 macroporous resin, eluted with an EtOH/H_2_O gradient (0:1, 1:9, 3:7, 5:5, 7:3, and 9:1), resulting in 5 fractions (Fr.1–Fr.5). Fraction Fr.3 (130 g, 50% EtOH) was further separated on MCI resin with a MeOH/H_2_O gradient (0:100 to 95:5), yielding 12 sub-fractions (Fr.3.1–Fr.3.12). Sub-fraction Fr.3.8, eluted with 80% MeOH, was subjected to medium-pressure liquid chromatography on an ODS column, eluted with a MeOH/H_2_O gradient (30% to 100% MeOH), and produced 9 sub-fractions (Fr.3.8.1–Fr.3.8.9). Fr.3.8.9 was further chromatographed on a Butyl 650C column, eluted with a MeOH/H_2_O gradient (10% to 100% MeOH), isolating compound **5** (15.1 mg) and **6** (19.0 mg). Fr.3.8.7 was purified using semi-preparative with CH_3_CN/0.1% TFA-H_2_O (35:65, *v*/*v*) under isocratic conditions, yielding compounds **1** (*t*_R_ = 20.57 min, 60.3 mg), **2** (*t*_R_ = 12.53 min, 11.4 mg), and **3** (*t*_R_ = 18.45 min, 201.4 mg). The 90% EtOH-eluted fraction (Fr.10) was separated using medium-pressure liquid chromatography on a C18 column, eluted with a MeOH/H_2_O gradient (50% to 100% MeOH), producing 7 sub-fractions (Fr.3.10.1–Fr.3.10.7). Fr.3.10.1 was further chromatographed on HW-40F resin with an EtOH/H_2_O gradient (0:1, 1:9, 3:7, 5:5, 7:3, 9:1), yielding compounds **7** (47.2 mg) and **10** (9.4 mg). Fr.3.10.2 was purified using a semi-preparative Welch XB-C18 column with CH_3_CN/0.1% TFA-H_2_O (18:82, *v*/*v*), yielding compounds **8** (*t*_R_ = 22.02 min, 161.7 mg), **9** (*t*_R_ = 41.95 min, 22.8 mg), and **15** (*t*_R_ = 95.82 min, 30.2 mg). Finally, Fr.3.12 (20 g) was separated on MCI resin with a MeOH/H_2_O gradient (from 0:100 to 95:5), affording 7 fractions (Fr.3.12.1–Fr.3.12.7). Fr.3.12.7 (4155 mg) was chromatographed on the Butyl 650C, eluted with a MeOH/H_2_O gradient (10% to 100% MeOH), isolating compounds **11** (427.3 mg) and **14** (52.7 mg). Meanwhile, Fr.3.12.6 (6074 mg) was purified using a semi-preparative Welch XB-C18 column with CH_3_CN/0.1% TFA-H_2_O (56:44, *v*/*v*), yielding compounds **4** (*t*_R_ = 45.37 min, 12.8 mg), **12** (*t*_R_ = 69.08 min, 55.4 mg), and **13** (*t*_R_ = 95.44 min, 333.1 mg).

#### 3.3.1. Compound **1**

Flavidoside A (**1**): Appears as a white amorphous powder; [*α*]^25^_D_ +25.9 (*c* 0.1, CH_3_OH); UV (CH_3_OH) *λ*_max_ (log *ε*) 204 (1.06); IR (ATR) *v*_max_ 3402, 2945, 1750, 1304, 1080 cm^−1^; NMR spectra recorded in Pyr-*d*_5_, refer to Table 1 and Table 2; HR-ESI-MS *m*/*z* 1157.5726 [M + Na]^+^ (calcd for C_55_H_90_O_24_Na^+^, 1157.5720).

#### 3.3.2. Compound **2**

Flavidoside B (**2**): Appears as a white amorphous powder; [α]^25^_D_ +40.1 (*c* 0.1, CH_3_OH); UV (CH_3_OH) *λ*_max_ (log *ε*) 211 (1.35); IR (ATR) *v*_max_ 3390, 2933, 1742, 1295, 1081 cm^−1^; NMR spectra recorded in Pyr-*d*_5_, refer to Table 1 and Table 2; HR-ESI-MS *m*/*z* 1143.5554 [M + Na]^+^ (calcd for C_54_H_88_O_24_Na^+^, 1143.5564).

#### 3.3.3. Compound **3**

Flavidoside C (**3**): Appears as a white powder; [*α*]^25^_D_ +84.6 (*c* 0.1, CH_3_OH); UV (CH_3_OH) *λ*_max_ (log *ε*) 200 (0.92); IR (ATR) *v*_max_ 3382, 2921, 1751, 1294, 1083 cm^−1^; NMR spectra recorded in Pyr-*d*_5_, refer to Table 1 and Table 2; HR-ESI-MS *m*/*z* 1141.5389 [M + Na]^+^ (calcd for C_54_H_86_O_24_Na^+^, 1141.5407).

#### 3.3.4. Compound **4**

Flavidoside D (**4**): Appears as a yellow amorphous powder; [*α*]^25^_D_ −19.5 (*c* 0.1, CH_3_OH); UV (CH_3_OH) *λ*_max_ (log *ε*) 196 (2.03), 236 (0.96), 290 (0.76); IR (ATR) *v*_max_ 3375, 1716, 1625 cm^−1^; ECD (*c* 1.0 mg/mL, CH_3_OH) *λ*_max_ (Δ*ε*) 254 (0.81), 292 (−10.50), 330 (2.09) nm; ^1^H-NMR and ^13^C-NMR in CD_3_OD, refer to Table 3; HR-ESI-MS *m*/*z* 765.2207 [M + Na]^+^ (calcd for C_33_H_42_O_19_Na^+^, 765.2218).

#### 3.3.5. Compound **5**

Flavidoside E (**5**): Appears as a yellow amorphous powder; [*α*]^25^_D_ −28.6 (*c* 0.1, CH_3_OH); UV (CH_3_OH) *λ*_max_ (log *ε*) 190 (1.82), 223 (1.07), 294 (0.66); IR (ATR) *v*_max_ 3380, 1721, 1633 cm^−1^; ECD (*c* 1.0 mg/mL, CH_3_OH) *λ*_max_ (Δ*ε*) 260 (1.07), 300 (−8.71), 350 (2.44) nm; ^1^H-NMR and ^13^C-NMR in CD_3_OD, see Table 4; HR-ESI-MS *m*/*z* 807.2327 [M + Na]^+^ (calcd for C_35_H_44_O_20_Na^+^, 807.2324).

#### 3.3.6. Compound **6**

Flavidoside F (**6**): Appears as a yellow solid; [*α*]^25^_D_ −16.3 (*c* 0.1, CH_3_OH); UV (CH_3_OH) *λ*_max_ (log *ε*) 191 (1.09), 220 (061), 294 (0.28); ECD (*c* 1.0 mg/mL, CH_3_OH) *λ*_max_ (log *ε*) 252 (0.94), 292 (-5.84), 334 (1.96); IR (ATR) *v*_max_ 3385, 1708, 1637 cm^−1^; ^1^H-NMR and ^13^C-NMR in DMSO-*d*_6_, see Table 4; HR-ESI-MS *m*/*z* 807.2311 [M + Na]^+^ (calcd for C_35_H_44_O_20_Na^+^, 807.2324).

#### 3.3.7. Compound **7**

Flavidoside G (**7**): Appears as a yellow powder; [*α*]^25^_D_ −85.9 (*c* 0.1, CH_3_OH); UV (CH_3_OH) *λ*_max_ (log *ε*) 209 (2.06), 251 (1.62), 270 (1.44), 310 (1.73), 358 (1.70); IR (ATR) *v*_max_ 3380, 1702, 1640, 1442, 1165 cm^−1^; ^1^H-NMR and ^13^C-NMR in CD_3_OD, see Table 5 and Table 6; HR-ESI-MS *m*/*z* 1021.2837 [M + H]^+^ (calcd for C_46_H_53_O_26_^+^, 1021.2825).

#### 3.3.8. Compound **8**

Flavidoside H (**8**): Appears as a yellow solid; [*α*]^25^_D_ −111.2 (*c* 0.1, CH_3_OH); UV (CH_3_OH) *λ*_max_ (log *ε*) 204 (1.85), 255 (150), 281 (1.29), 310 (1.42), 350 (1.51); IR (ATR) *v*_max_ 3395, 1711, 1651, 1444, 1160 cm^−1^; ^1^H-NMR and ^13^C-NMR in DMSO-*d*_6_, see Table 5 and Table 6; HR-ESI-MS *m*/*z* 1073.2747 [M + Na]^+^ (calcd for C_47_H_54_O_27_Na^+^, 1073.2750).

#### 3.3.9. Compound **9**

Flavidoside I (**9**): Appears as a yellow powder; [*α*]^25^_D_ −71.4 (*c* 0.1, CH_3_OH); UV (CH_3_OH) *λ*_max_ (log *ε*) 200 (2.52), 244 (2.01), 277 (1.85), 304 (2.09), 366 (1.97); IR (ATR) *v*_max_ 3383, 1708, 1643, 1450, 1162 cm^−1^; ^1^H-NMR and ^13^C-NMR in CD_3_OD, see Table 5 and Table 6; HR-ESI-MS *m*/*z* 1035.3014 [M + H]^+^ (calcd for C_47_H_55_O_26_^+^, 1035.2981).

#### 3.3.10. Compound **10**

Flavidoside J (**10**): Appears as a yellow powder; [*α*]^25^_D_ −42.6 (*c* 0.1, CH_3_OH); UV (CH_3_OH) *λ*_max_ (log *ε*) 201 (1.47), 262 (1.08), 282 (0.96), 305 (0.82), 364 (0.94); IR (ATR) *v*_max_ 3395, 1713, 1640, 1436, 1159 cm^−1^; ^1^H-NMR and ^13^C-NMR in CD_3_OD, see Table 5 and Table 6; HR-ESI-MS *m*/*z* 1005.2885 [M + H]^+^ (calcd for C_46_H_53_O_25_^+^, 1005.2876).

#### 3.3.11. Purity Statement

The purity of all compounds was verified to be ≥95% by HPLC analysis.

### 3.4. Acid Hydrolysis

Acid hydrolysis and the identification of monosaccharides followed the method described in the previous literature [27].

### 3.5. ECD Calculation

ECD calculations were performed using the method described in a previous paper from our laboratory [7].

### 3.6. Cytotoxicity Assays

#### 3.6.1. Cell Culture and Treatment

The human cervical cancer cell line Hela, the human lung adenocarcinoma cell line A549, the human breast cancer cell lines MCF-7 and MDA-MB-231, the human hepatoma cell lines BEL-7402 and Hep G2, the human embryonic lung fibroblast cell line MRC-5, and the human nasal epithelial cell line HNEpC were all sourced from the China Center for Type Culture Collection (CCTCC, Wuhan University, Hubei, China). Hela, A549, MCF-7, MDA-MB-231, BEL-7402, MRC-5, and Hep G2 cells were maintained in DMEM media, while HNEpC cells were cultured in RPMI 1640. All media were supplemented with 10% FBS (*v*/*v*) and 1% penicillin-streptomycin, and cells were grown in a humidified atmosphere containing 5% CO_2_ at 37 °C.

#### 3.6.2. Cell Viability Assay

The MTT assay was conducted to assess cell viability, as previously described [28]. Cells were seeded onto 96-well plates at a density of 1 × 10^4^ cells per well, with each experiment replicated eight times to ensure reproducibility. Following incubation at 37 °C for 24 h, cells were exposed to various concentrations (0.25, 0.5, 1.0, 2.0, 4.0, 8.0, 16.0, 32.0 µM) of compounds **1**–**10** for 24 h. Subsequently, 10 μL MTT reagent was added to each well, and the plates were incubated for a further 4 h at 37 °C, adhering strictly to the manufacturer’s instructions. The optical density (OD) values were then measured at 570 nm using a microplate reader. The cell survival rate for each compound was calculated employing the following formula:

$$\left(\frac{OD\;value\;of\;the\;experimental\;group-OD\;value\;of\;the\;blank\;group}{OD\;value\;of\;the\;control\;group-OD\;value\;of\;Blank\;group}\right)\times 100\%$$

### 3.7. α-Glycosidase Inhibitory Assays

The anti-α-glycosidase effects of compound **1**–**10** were screened using a previously reported method. Briefly, 40 µL of each target compound **1**–**10** at various concentrations and 20 µL of α-glycosidase solution (0.25 U/mL) were added to individual wells of a 96-well plate, constituting the experimental group (EG). These were incubated for 10 min at 37 °C. For the sample background control group (SBCG), 40 μL of solutions containing compounds **1**–**10** and 20 µL of phosphate buffer solution (50 mM) were added. In the blank group (BG), 20 µL of α-glucosidase solution (0.25 U/mL) and 40 µL of 50 mmol/L phosphate buffer solution (PBS) were combined. Additionally, 60 µL of PBS (50 mM) were added to wells designated as the blank control group (BCG). Subsequently, 50 µL of *p*-nitrophenyl-α-D-glucopyranoside solution (1 mM), serving as the substrate, were added to each well of the EG and incubated for 30 min. After incubation, the reaction was terminated by adding 50 µL of sodium carbonate solution (0.2 mM). The absorbance of each well was measured at 405 nm and the values were recorded as A_1_ for EG, A_2_ for SBCG, B_1_ for BG, and B_2_ for BCG.

The inhibition rate for each compound was calculated using the following formula:

$$(1-\frac{A_{1}-A_{2}}{B_{1}-B_{2}})\times 100\%$$

### 3.8. Molecular Docking Investigation

Autodock Vina (Version 1.1.2), a protein-ligand docking software, was used to evaluate the binding affinity and interaction modes of compounds **1**–**10** with their target. The 3D coordinates of α-glucosidase (PDB ID: 3A4A, resolution 1.6 Å) were obtained from the Protein Data Bank (RCSB PDB. Available online: https://www.rcsb.org, Accessed on 8 September 2024). All water molecules were removed, and polar hydrogen atoms were added. Grid boxes were designed to encompass the structural domains of each protein, permitting the ligands to move freely. The docking pocket was defined as a cubic region measuring 126 Å × 126 Å × 126 Å, with grid spacing set at 0.375 Å. Optimal molecular interactions were determined by the binding characteristics of α-glucosidase residues and their corresponding binding affinity scores.

### 3.9. Statistical Analysis

Statistical analysis data from the cytotoxicity and α-glycosidase inhibitory assays were analyzed using the mean and standard deviation. The 50% cytotoxic and inhibitory concentrations were calculated and compared to the control via non-linear regression. These analyses were performed using SPSS^®^ Statistics (Version 18.0, IBM Software, Armonk, NY, USA).

## 4. Conclusions

This study examined the bioactive constituents and activities of *Camellia flavida* as sources for tea and functional foods. Ten new glycoside compounds (flavidosides A–J), comprising three oleanane-type triterpenoid saponins and seven flavonoid glycosides, were isolated from the flowers of *C*. *flavida*. Tests were conducted on the cytotoxic effects of the compounds against cancer cells and their α-glucosidase inhibitory activity. The IC_50_ values recorded for flavidoside C against MCF-7 and BEL-7402 cells were 1.65 ± 0.39 and 4.94 ± 0.41 μM, respectively. The semi-inhibitory concentration of flavidoside H on α-glucosidase was 1.17 ± 0.30 mM. These findings suggest that the bioactive constituents in *C. flavida* could potentially slow tumor growth and inhibit α-glucosidase activity, highlighting their promise for further research in anti-tumor treatments and metabolic regulation. We are now planning to undertake more comprehensive research to gain a deeper understanding of the underlying mechanisms, with the goal of identifying superior lead compounds.

## Figures and Tables

**Table 1:** ^1^H-NMR (600 MHz) data of compounds **1**–**3** in Pyr-*d*_5_ (*δ*_H_, mult; *J* in Hz).

No.	1	2	3
1	0.83 m 1.42 m	0.81 m 1.37 m	0.76 m 1.35 m
2	1.68 m 1.97 m	1.71 m 2.06 m	1.72 m 2.05 m
3	3.17 dd (11.7, 4.1)	3.20 dd (11.3, 3.9)	3.17 dd (11.5, 3.8)
5	0.77 br d (12.3)	0.77 br d (11.9)	0.72 br d (11.6)
6	1.33 m 1.53 m	1.34 m 1.54 m	1.31 m 1.50 m
7	2.03 m 2.15 m	2.03 m 2.15 m	1.85–1.97 m
9	1.68 m	1.68 m	1.49, m
11	1.82, m	1.84, m	1.85, m
12	5.45 t (3.4)	5.44 t (3.4)	5.46 t (3.4)
15	4.48 overlapped	4.48 overlapped	5.09 s
16	4.55 d (3.8)	4.54 d (3.7)	
18	2.45 dd (13.5, 3.8)	2.44 dd (13.5, 3.8)	2.71 dd, 13.9, 3.4)
19	1.30 overlapped 2.70 dd (13.5, 12.9)	1.29 overlapped 2.70 dd (13.5, 12.9)	1.21 overlapped 1.56 t-like (13.9)
21	1.40 m 2.39 m	1.39 m 2.39 m	1.29 m 1.76 m
22	2.17 m 2.27 m	2.16 m 2.27 m	1.53 overlapped 2.88 br d (13.7)
23	1.12 s	1.13, s	1.10, s
24	1.02 s	1.04, s	1.04, s
25	0.82 s	0.81, s	0.79, s
26	1.05 s	1.04, s	1.13, s
27	1.81 s	1.82, s	1.34, s
28	3.60 d (10.5) 3.79 d (10.5)	3.60, d (10.5) 3.79, d (10.5)	3.81, d (10.8) 4.42, overlapped
29	1.05 s	1.04, s	0.85, s
30	1.10 s	1.10, s	0.89, s
GlcA-1	4.86 overlapped	4.86, overlapped	4.82, d (7.1)
2	4.68 m	4.73, m	4.71, m
3	4.67 m	4.74, m	4.74, m
4	4.52 m	4.64, m	4.63, dd (9.5, 9.0)
5	4.45 overlapped	4.57, m	4.57, d (9.5)
6-OCH_3_	3.69 s		
Glc-1	5.87 d (7.4)	5.90 d (7.4)	5.90 d (7.4)
2	4.10 dd (9.0, 7.4)	4.12 dd (9.3, 7.4)	4.10 dd (9.0, 7.4)
3	4.40 m	4.41 m	4.39 m
4	4.07 t-like (9.1)	4.07 t-like (9.0)	4.04 t-like (9.1)
5	4.40 m	4.41 m	4.39 m
6	4.31 m 4.66 m	4.31 m 4.66 m	4.28 m 4.66 m
Gal-1	6.10 d (7.7)	6.18 d (7.6)	6.21 overlapped
2	4.69 m	4.71 m	4.71 m
3	4.45 m	4.48 m	4.49 br d (9.2)
4	4.45 m	4.46 m	4.44 br s
5	4.23 m	4.26 m	4.27 m
6	4.31 m	4.32 m	4.33 m
Rha-1	6.23 br s	6.23 br s	6.21 br s
2	4.77 br s	4.78 br s	4.76 br s
3	4.71 m	4.72 m	4.69 m
4	4.20 t-like (9.2)	4.21 t-like (9.1)	4.20 t-like (9.1)
5	4.84 m	4.88 m	4.89 m
6	1.41 d (6.1)	1.42 d (5.8)	1.40 d (5.8)

**Table 2:** ^13^C-NMR (151 MHz) data of compounds **1**–**3** in Pyr-*d*_5_ (*δ*_C_).

No.	1	2	3
1	39.0	39.0	39.0
2	26.4	26.5	26.6
3	89.7	89.8	89.8
4	39.6	39.7	39.7
5	55.6	55.6	55.6
6	18.8	18.9	18.7
7	36.8	36.8	36.1
8	41.4	41.5	41.8
9	47.4	47.3	47.1
10	37.0	37.1	37.0
11	24.0	24.0	24.1
12	123.9	123.9	126.0
13	146.0	146.0	142.0
14	48.0	48.0	54.3
15	67.4	67.4	73.8
16	78.7	78.7	216.1
17	41.1	41.1	53.2
18	43.3	43.3	48.1
19	48.0	48.0	48.0
20	31.3	31.3	31.0
21	37.1	37.1	35.8
22	31.0	31.0	27.1
23	28.1	28.0	28.0
24	16.8	16.8	16.9
25	15.9	15.9	15.8
26	17.6	17.6	17.7
27	21.0	21.0	21.7
28	69.6	69.6	70.9
29	33.5	33.5	33.1
30	24.6	24.6	23.6
GlcA-1	105.2	105.4	105.4
2	79.5	79.5	79.6
3	82.8	82.8	82.7
4	70.9	71.3	71.3
5	76.6	77.4	77.4
6	170.3	172.9	172.2
6-OCH_3_	52.1		
Glc-1	102.9	102.7	102.8
2	76.4	76.4	76.5
3	78.5	78.5	78.5
4	72.6	72.6	72.7
5	78.2	78.1	78.2
6	63.5	63.6	63.7
Gal-1	101.6	101.4	101.4
2	76.2	76.4	76.5
3	76.1	76.0	76.1
4	71.1	71.2	71.3
5	77.0	77.0	77.1
6	62.0	62.0	62.1
Rha-1	102.3	102.4	102.4
2	72.6	72.6	72.7
3	72.6	72.6	72.7
4	73.9	74.0	74.0
5	69.8	69.8	69.9
6	18.3	18.3	18.3

**Table 3:** ^1^H (600 MHz) and ^13^C (151 MHz) NMR data of compound **4** in CD_3_OD.

No.	*δ*_H_ (*J* in Hz)	*δ*_C_	No.	*δ*_H_ (*J* in Hz)	*δ*_C_
2	5.19 d (10.4)	83.5	4	3.29 overlapped	73.9
3	4.55 d (10.4)	81.2	5	4.18 m	70.9
4		195.7	6	1.20 d (6.2)	17.9
5		165.5	Glc-1	4.02 d (7.6)	105.3
6	5.92 d (1.9)	97.4	2	3.30 overlapped	78.9
7		168.6	3	3.41 t-like (9.1)	78.3
8	5.90 d (1.9)	96.3	4	3.33 overlapped	70.9
9		164.0	5	3.10 dt (9.5, 3.1)	77.2
10		102.5	6	3.68 d (3.1)	62.1
1′		128.7	Rha2-1	5.21 d (1.5)	101.8
2′, 6′	7.36 d (8.4)	130.1	2	3.88 dd (3.3, 1.5)	71.9
3′, 5′	6.85 d (8.4)	116.5	3	3.79 dd (9.5, 3.3)	71.8
4′		159.2	4	3.33 overlapped	74.2
Rha1-1	4.71 d (1.4)	102.1	5	4.02 m	70.3
2	3.46 dd (3.2, 1.4)	82.4	6	1.26 d (6.2)	17.9
3	3.72 dd (9.8, 3.2)	71.6			

**Table 4:** ^1^H (600 MHz) and ^13^C (151 MHz) NMR data of compounds **5** and **6**.

No.	5 ^1^		6 ^2^	
	*δ*_H_ (*J* in Hz)	*δ*_C_	*δ*_H_ (*J* in Hz)	*δ*_C_
2	5.48 d (2.3)	82.1	5.42 d (8.4)	80.9
3	4.17 d (2.3)	75.8	4.71 d (8.4)	76.9
4		194.4		193.8
5		166.1		163.4
6	5.93 d (1.7)	97.5	5.90 s	96.0
7		168.9		167.1
8	5.97 d (1.7)	96.3	5.90 s	95.1
9		164.6		161.9
10		101.9		101.0
1′		128.0		126.2
2′, 6′	7.31 d (8.5)	129.0	7.25 d (8.4)	128.8
3′, 5′	6.82 d (8.5)	116.3	6.78 d (8.4)	115.3
4′		158.7		157.7
5-OH			11.71 br s	
7-OH			10.96 br s	
4′-OH			9.62 br s	
Rha1-1	5.36 d (1.2)	99.6	4.77 br s	99.7
2	3.73 br s	81.8	3.49 br s	77.9
3	3.53 dd (9.7 3.4)	71.6	3.09 m	70.1
4	3.18 t-like (9.7)	73.4	3.15 m	72.1
5	2.38 dq (9.7 6.1)	70.3	3.60 m	69.0
6	0.88 d (6.1)	17.9	1.07 d (6.0)	17.6
Glc-1	4.47 d (7.7)	105.5	4.31 d (7.8)	102.5
2	3.41 overlapped	78.4	3.17 m	75.6
3	3.46 t-like (9.0)	78.7	3.28 m	77.5
4	3.39 overlapped	71.1	3.46 m	70.0
5	3.20 m	77.4	3.07 m	76.6
6	3.74 m 3.85 m	62.4	3.48 m 3.64 m	60.8
Rha2-1	5.27 d (1.8)	101.3	5.18 br s	99.5
2	4.00 dd (3.2 1.8)	69.8	3.70 br s	70.3
3	5.03 dd (9.8 3.2)	75.5	3.75 m	67.7
4	3.50 t-like (9.8)	71.6	4.72 t-like (9.8)	74.1
5	4.19 m	70.1	4.07 m	65.7
6	1.24 d (6.2)	17.9	0.94 d (6.1)	17.4
Acetyl-1		172.8		170.2
2	2.08 s	21.1	2.00 s	21.0

^1^ Measured in CD_3_OD. ^2^ Measured in DMSO-*d*_6_.

**Table 5:** ^1^H-NMR (600 MHz) data (*J* in Hz) of compounds **7**–**10**.

No.	7 ^1^	8 ^2^	9 ^1^	10 ^1^
6	6.13 br s	6.15 br s	6.17 br s	6.15 br s
8	6.32 br s	6.33 br s	6.35 br s	6.35 br s
2′	7.60 br s	7.51 overlapped	7.98 d (8.3)	7.98 d (8.6)
3′			6.89 d (8.3)	6.90 d (8.6)
5′	6.88 d (7.8)	6.84 d (8.8)	6.89 d (8.3)	6.90 d (8.6)
6′	7.55 br d (7.8)	7.51 overlapped	7.98 d (8.3)	7.98 d (8.6)
5-OH		12.58 br s		
Glc1-1	5.58 d (7.8)	5.57 d (7.9)	5.53 d (8.0)	5.57 d (8.0)
2	5.24 dd (9.5, 7.8)	5.09 dd (9.5, 7.9)	5.20 dd (9.5, 8.0)	5.19 dd (9.5, 8.0)
3	3.88 m	3.82 t-like (9.0)	3.90 t-like (9.2)	3.87 t-like (9.0)
4	3.48 m	3.29 m	3.43 m	3.42 t-like (9.2)
5	3.58 m	3.52 m	3.52 m	3.54 m
6	3.51 m 3.88 m	3.41 overlapped 3.73 br d (11.3)	3.47 m 3.86 m	3.47 m 3.87 m
Ara-1	4.34 d (6.8)			4.34 d (7.0)
2	3.54 m			3.53 m
3	3.49 m			3.47 m
4	3.77 br s			3.77 br s
5	3.56 m 3.85 m			3.56 m 3.87 m
Glc2-1		4.31 d (7.9)	4.42 d (7.7)	
2		2.95 m	3.18 dd (9.0, 7.7)	
3		3.20 m	3.28 m	
4		3.26 m	3.27 m	
5		3.10 m	3.32 m	
6		3.41 overlapped 3.68 m	3.62 dd (11.6, 6.3) 3.86 m	
Rha-1	4.58 br s	4.38 br s	4.54 br s	4.54 d (1.5)
2	3.85 br s	3.57 br s	3.80 br s	3.81 dd (3.1, 1.5)
3	3.60 dd (9.2, 3.0)	3.32 m	3.55 dd (9.3, 3.1)	3.56 m
4	3.44 t-like (9.4)	3.26 m	3.42 t-like (9.4)	3.43 t-like (9.4)
5	3.53 m	3.32 m	3.49 m	3.51 m
6	1.13 d (6.0)	0.96 d (6.0)	1.11 d (6.1)	1.12 d (6.1)
Xyl-1	4.39 d (7.4)	4.24 d (7.5)	4.35 d (7.5)	4.36 d (7.5)
2	3.26 dd (9.1, 7.4)	3.02 m	3.26 dd (9.0, 7.5)	3.25 dd (9.1, 7.5)
3	3.37 t-like, (9.0)	3.12 m	3.35 t-like (9.0)	3.35 t-like (9.0)
4	3.49 m	3.03 m	3.49 m	3.49 m
5	3.17 t-like (10.5) 3.80 m	3.00 m 3.60 dd (11.1, 5.0)	3.15 t-like (10.7) 3.79 m	3.15 t-like (10.8) 3.79 m
Coumaroyl-2, 6	7.45 d (8.0)	7.51 d (8.4)	7.47 d (8.2)	7.46 d (8.2)
3, 5	6.80 d (8.0)	6.79 d (8.4)	6.81 d (8.2)	6.80 d (8.2)
7	7.68 d (15.9)	7.54 d (15.9)	7.70 d (15.9)	7.69 d (15.9)
8	6.42 d (15.9)	6.36 d (15.9)	6.39 d (15.9)	6.39 d (15.9)

^1^ Measured in CD_3_OD. ^2^ Measured in DMSO-*d*_6_.

**Table 6:** ^13^C-NMR (151 MHz) data of compounds **7**–**10** (*δ*_C_).

No.	7 ^1^	8 ^2^	9 ^1^	10 ^1^
2	158.9	156.8	159.1	159.1
3	134.8	132.8	134.6	134.7
4	178.9	177.0	179.0	179.0
5	162.9	161.2	163.0	163.0
6	99.9	98.6	99.9	99.9
7	165.5	164.1	165.6	165.6
8	94.8	93.6	94.9	94.9
9	158.3	156.4	158.4	158.5
10	105.9	104.0	105.8	105.9
1′	123.3	120.9	122.8	122.9
2′	117.5	116.3	132.3	132.3
3′	145.8	144.8	116.2	116.2
4′	149.6	148.6	161.3	161.3
5′	116.2	115.2	116.2	116.2
6′	123.5	121.7	132.3	132.3
Glc1-1	100.7	98.8	100.7	100.7
2	74.5	72.2	74.4	74.5
3	84.5	83.1	84.6	84.3
4	70.3	69.2	70.4	70.4
5	76.8	75.3	76.9	76.9
6	68.4	67.9	68.6	68.5
Ara-1	105.2			105.3
2	72.2			72.2
3	73.9			73.9
4	69.5			69.5
5	67.2			67.2
Glc2-1		103.3	104.8	
2		73.2	74.7	
3		76.8	77.6	
4		69.5	71.3	
5		76.4	78.0	
6		61.0	62.5	
Rha-1	102.2	101.3	102.2	102.2
2	71.6	69.8	71.7	71.7
3	82.4	81.1	82.4	82.4
4	72.7	70.7	72.6	72.7
5	69.5	68.0	69.5	69.5
6	17.9	17.6	17.9	17.9
Xyl-1	106.2	105.4	106.3	106.3
2	75.3	73.9	75.2	75.3
3	77.5	76.0	77.5	77.5
4	71.1	69.9	71.0	71.1
5	66.8	65.5	66.8	66.8
Coumaroyl-1	127.3	125.3	127.3	127.4
2, 6	131.3	130.2	131.3	131.3
3, 5	116.8	115.8	116.8	116.8
4	161.1	159.7	161.2	161.2
7	147.3	144.6	147.2	147.3
8	115.1	114.4	115.2	115.2
9	168.8	165.7	168.6	168.6

^1^ Measured in CD_3_OD. ^2^ Measured in DMSO-*d*_6_.

**Table 7:** Cytotoxicity (IC_50_, μM ± SD, *n* = 8) of compounds **1**–**10** against cell lines.

	Hela	MCF-7	BEL-7402	A549	Hep G2	MB-231	MRC-5	HNEpC
**1**	15.07 ± 1.06	9.06 ± 0.45	7.62 ± 1.06	>30	25.60 ± 3.46	>30	>30	>30
**2**	4.17 ± 0.85	19.24 ± 3.71	>30	>30	14.04 ± 3.29	26.53 ± 3.07	>30	>30
**3**	>30	1.65 ± 0.39	4.94 ± 0.41	23.42 ± 7.26	8.77 ± 1.56	10.08 ± 2.28	>30	>30
**4**	>30	>30	>30	>30	>30	>30	>30	>30
**5**	>30	>30	>30	>30	20.76 ± 3.79	>30	>30	>30
**6**	>30	>30	11.40 ± 3.11	>30	>30	>30	>30	>30
**7**	>30	>30	>30	>30	>30	>30	>30	>30
**8**	>30	>30	>30	>30	>30	>30	>30	>30
**9**	24.72 ± 4.39	>30	>30	>30	28.80 ± 5.55	>30	>30	>30
**10**	>30	>30	>30	17.38 ± 2.81	>30	>30	>30	>30
PC	11.06 ± 0.72 ^1^	3.79 ± 0.26 ^2^	9.94 ± 0.80 ^1^	4.20 ± 0.37 ^1^	6.31 ± 0.18 ^1^	9.06 ± 1.01 ^1^	ND	ND

Positive control (PC): ^1^ cisplatin and ^2^ doxorubicin.

**Table 8:** α-Glucosidase inhibition activity (IC_50_, mM ± SD, *n* = 3) of compounds **1**–**10**.

	IC_50_		IC_50_
**1**	>20	**7**	2.38 ± 0.22
**2**	>20	**8**	1.17 ± 0.30
**3**	>20	**9**	3.33 ± 0.48
**4**	6.15 ± 0.43	**10**	2.26 ± 0.48
**5**	12.73 ± 1.08	acarbose	2.04 ± 0.27
**6**	8.09 ± 2.61		

## Data Availability

The data presented in this study are available in the Supplementary Materials.

## Supplementary Materials

The following supporting information can be downloaded at https://www.mdpi.com/article/10.3390/ijms252010977/s1.
