# Supplementary material for: Triterpenoid Saponins and Flavonoid Glycosides from the Flower of Camellia flavida and Their Cytotoxic and α-Glycosidase Inhibitory Activities
Source: Int J Mol Sci. 2024 Oct 12;25(20):10977. doi: 10.3390/ijms252010977 (PMC11506924; doi:10.3390/ijms252010977)
Supplement: Supplementary file 1 [file ijms-25-10977-s001.zip › ijms-3224414-supplementary.pdf]

Supplementary Materials

Triterpenoid Saponins and Flavonoid Glycosides from the Flower of *Camellia flavida* and Their Cytotoxic and α-Glycosidase Inhibitory Activities

Siyuan Ma [1,†], Yuxin Wu [1,†], Hanfeng Min [1], Li Ge [1,*], and Kedi Yang [1,*]

[1] Medical College of Guangxi University, Nanning 530004, China

* Correspondence: geli_2009@163.com (L.G.); kdyang@163.com (K.Y.); Tel.: +86-771-3949953 (K.Y.)

† These authors contributed equally to this work.

CONTENTS

1. Spectroscopic data for compound **1** 1

2. Spectroscopic data for compound **2** 6

3. Spectroscopic data for compound **3** 11

4. Spectroscopic data for compound **4** 17

5. Spectroscopic data for compound **5** 22

6. Spectroscopic data for compound **6** 27

7. Spectroscopic data for compound **7** 32

8. Spectroscopic data for compound **8** 36

9. Spectroscopic data for compound **9** 41

10. Spectroscopic data for compound **10** 46

11. ^{1}H and ^{13}C NMR spectroscopic data for compound **11−15** 51

12. Molecular docking results of compounds **1−7** and **9−10** with *α*-Glycosidase....... 56

1. Spectroscopic data for compound **1**

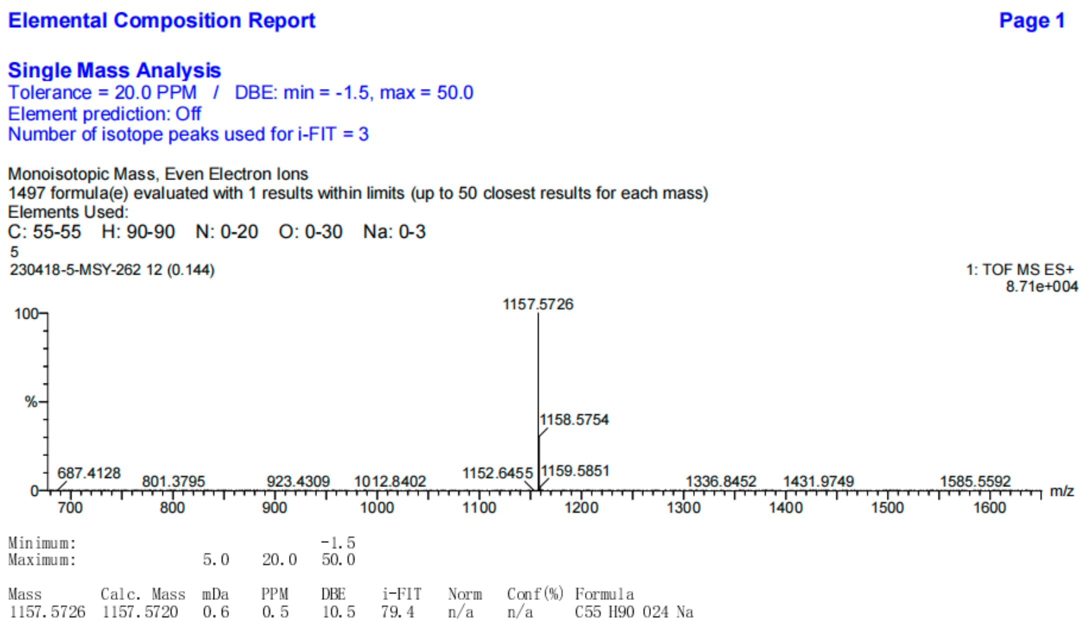

Figure S1. The HR-ESI-MS of compound **1**.

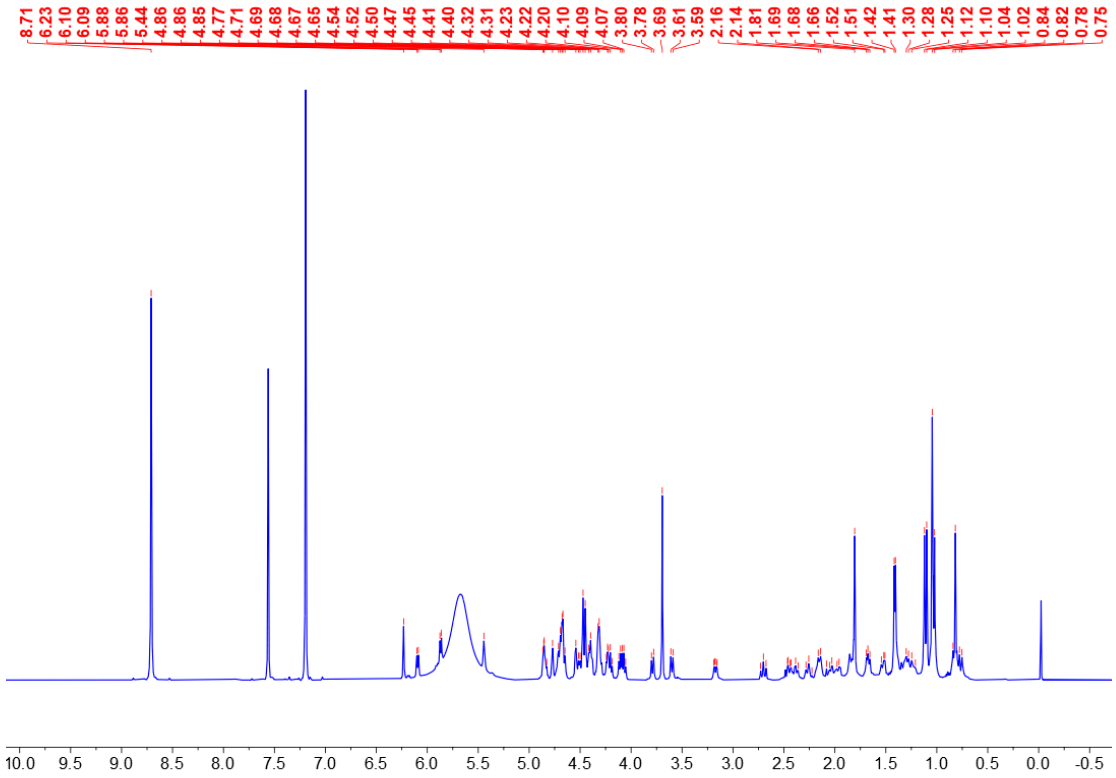

Figure S2. The ^{1}H NMR spectrum of compound **1** (600 MHz, Pyr-d_5).

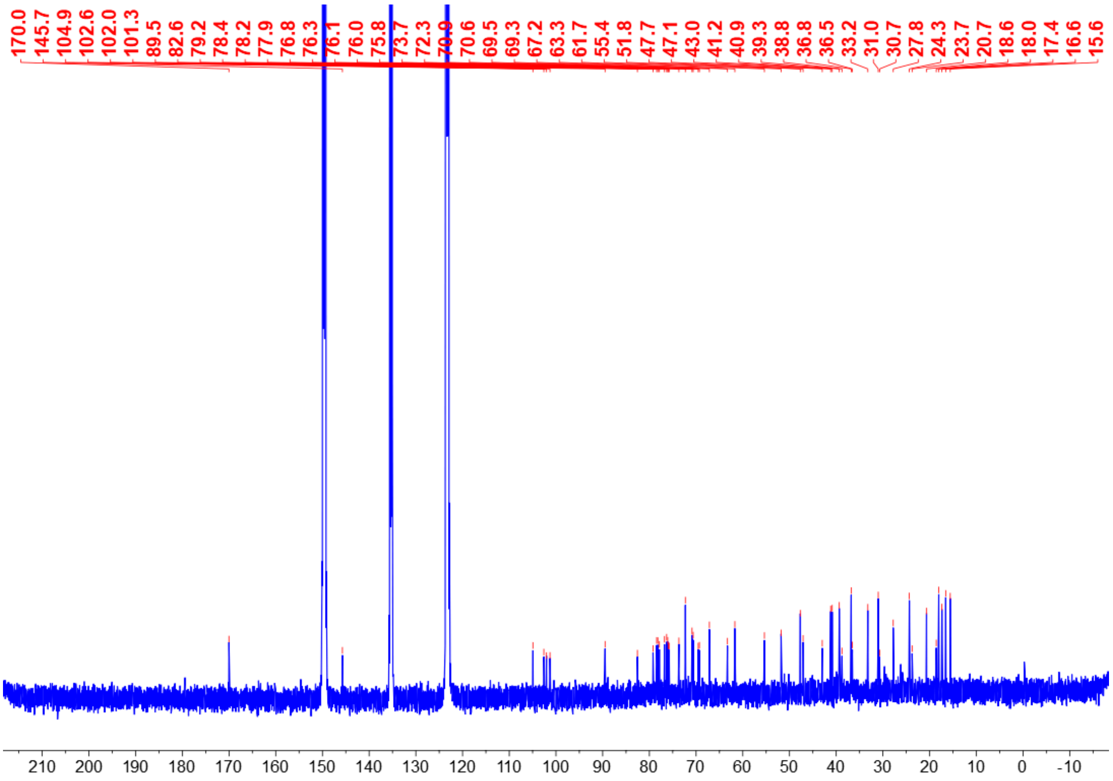

Figure S3. The ^{13}C NMR spectrum of compound **1** (151 MHz, Pyr-d_5).

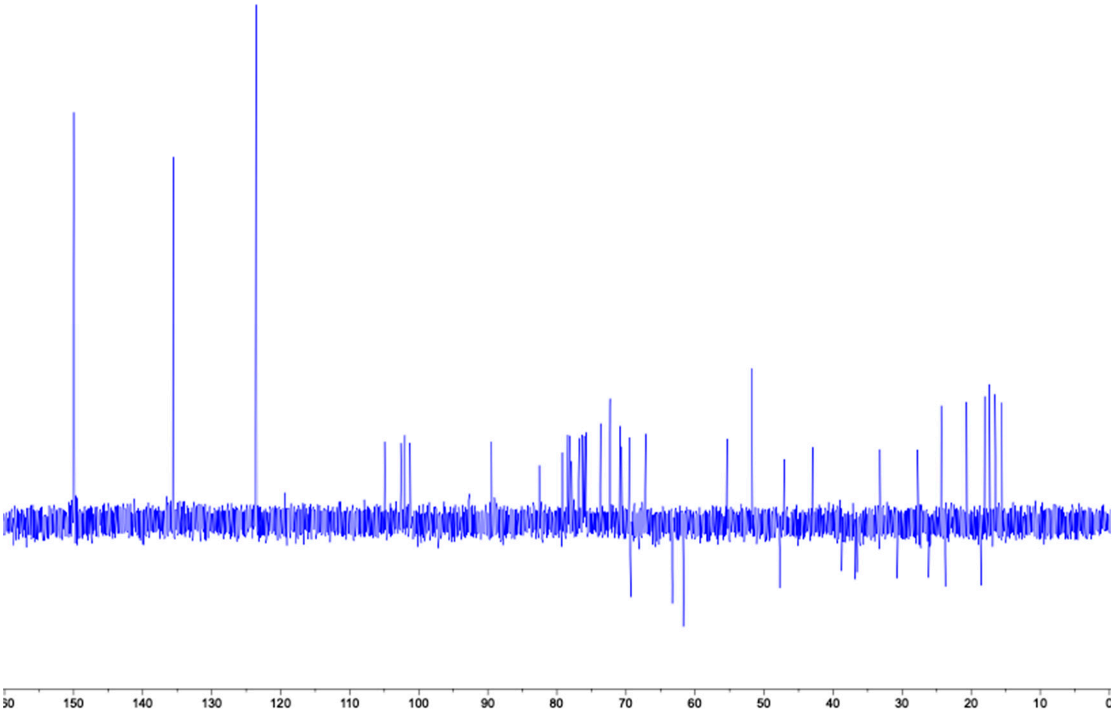

Figure S4. The DEPT 135 spectrum of compound **1** (151 MHz, Pyr-d_5).

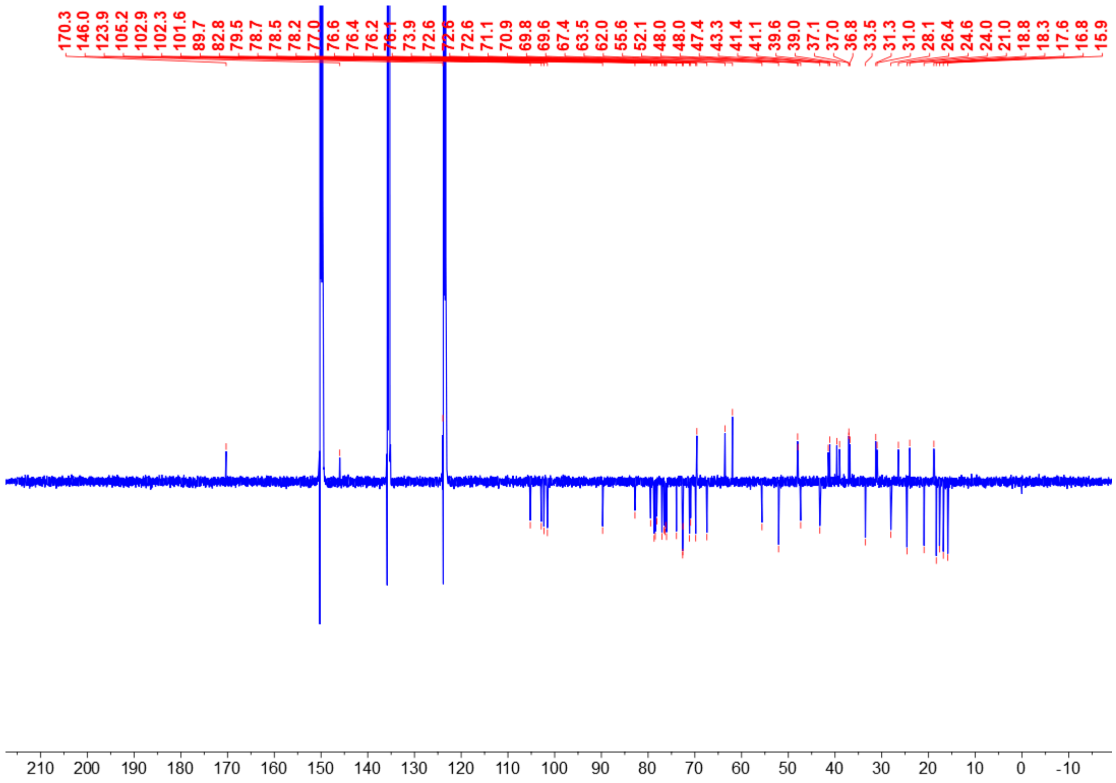

Figure S5. The DEPTQ spectrum of compound **1** (151 MHz, Pyr-d_5).

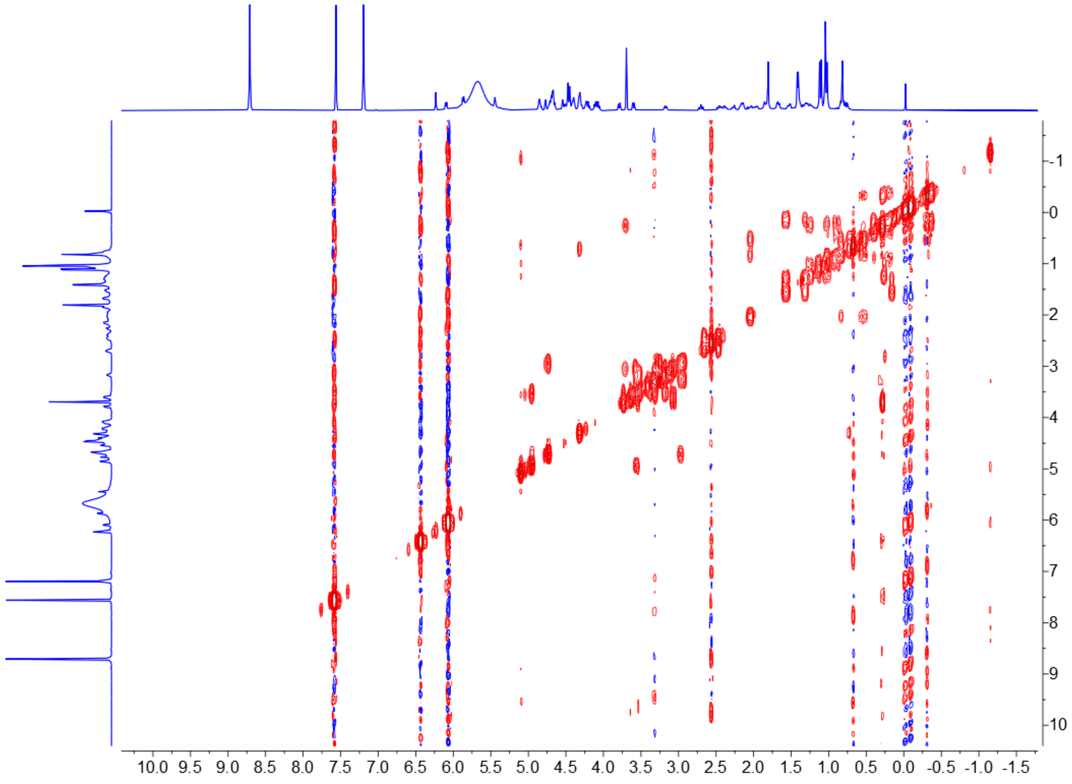

Figure S6. The ^{1}H-^{1}H COSY spectrum of compound **1** (600 MHz, Pyr-d_5).

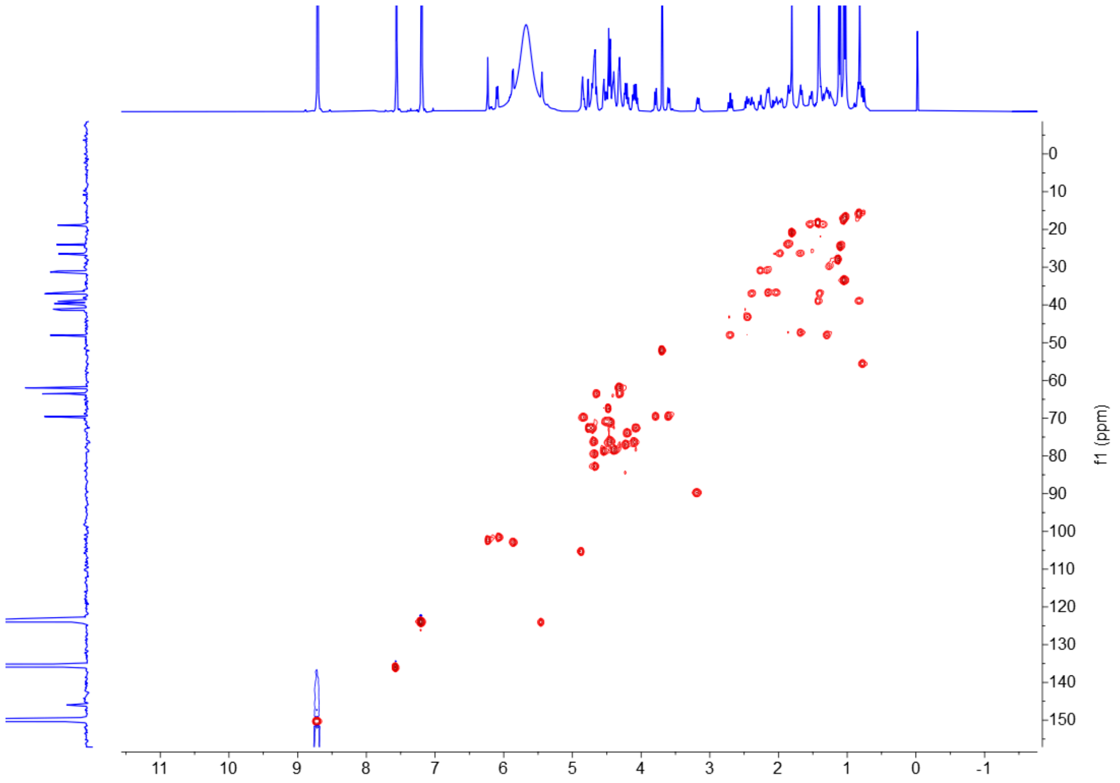

Figure S7. The HSQC spectrum of compound **1** (600/151 MHz, Pyr-d_5).

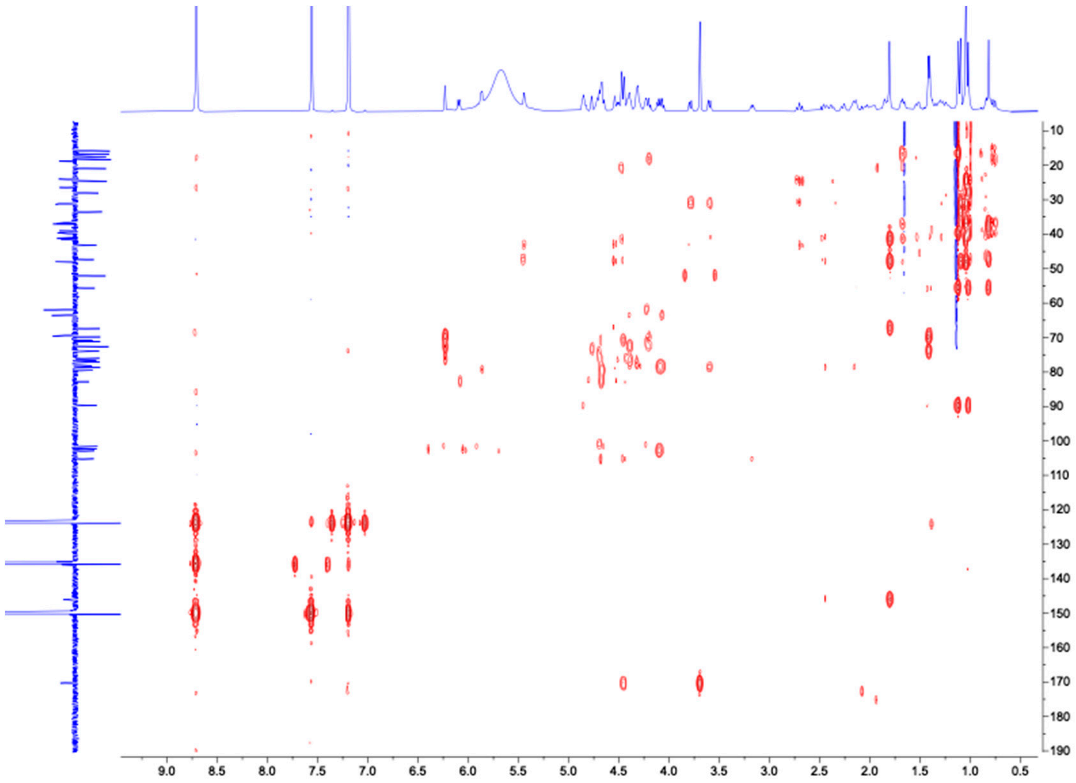

Figure S8. The HMBC spectrum of compound **1** (600/151 MHz, Pyr-d_5).

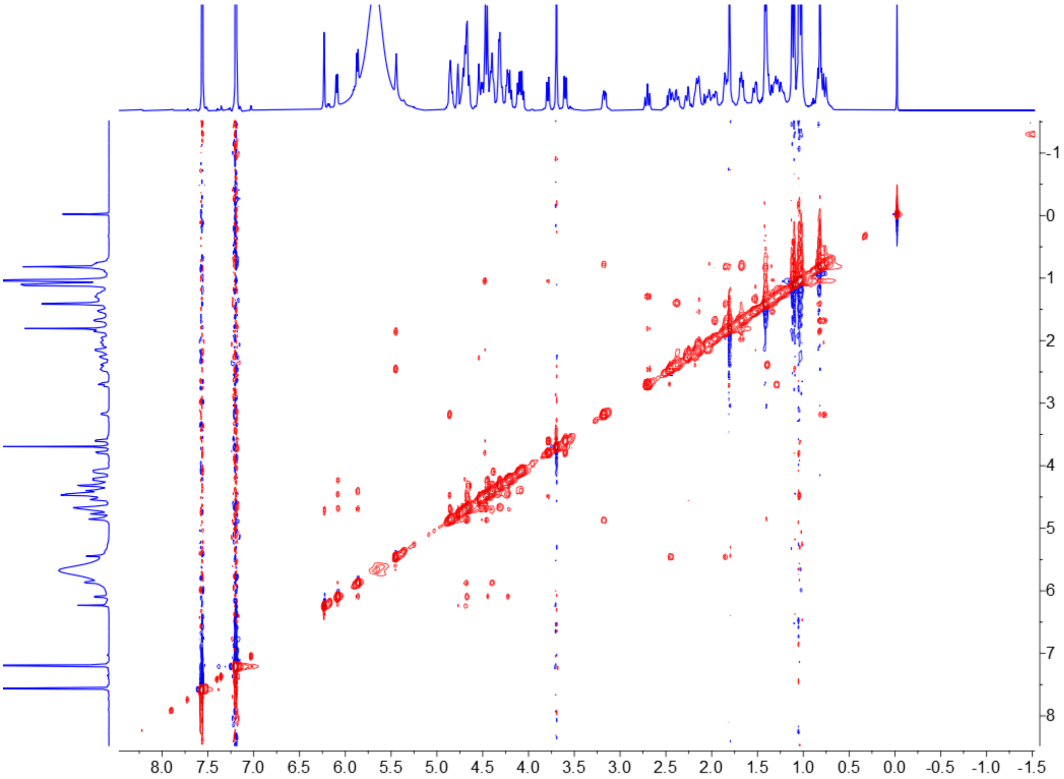

Figure S9. The NOESY spectrum of compound **1** (600 MHz, Pyr-d_5).

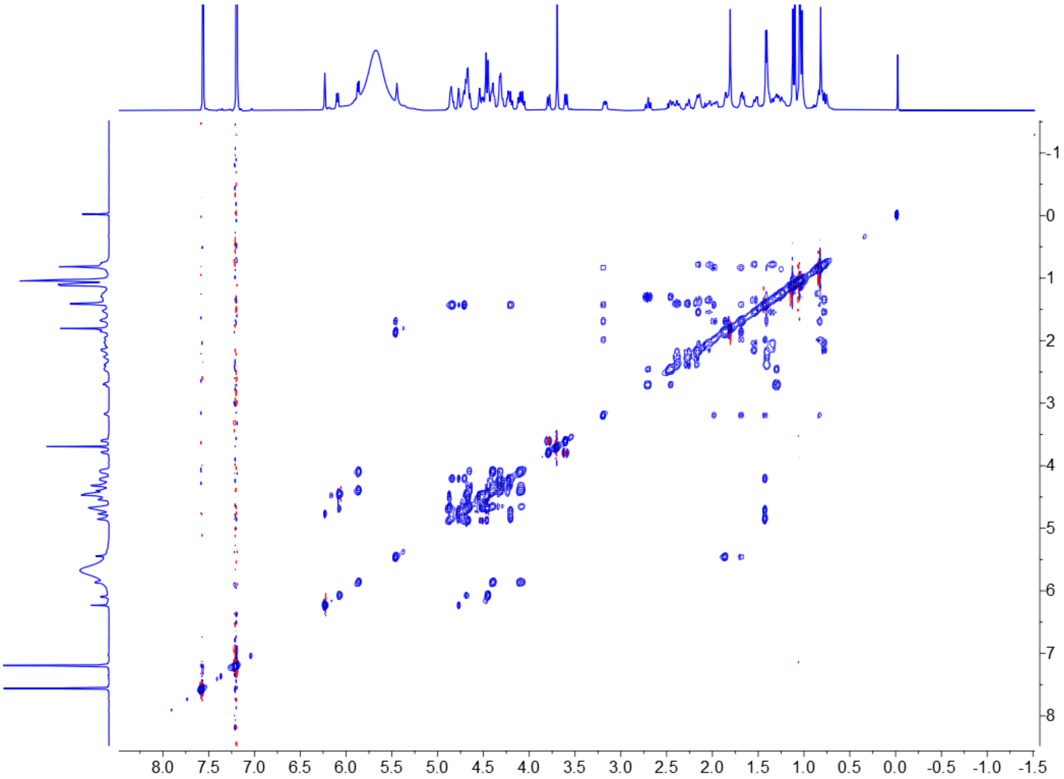

Figure S10. The TOCSY spectrum of compound **1** (600 MHz, Pyr-d_5).

2. Spectroscopic data for compound **2**

Elemental Composition Report **Page 1**

Single Mass Analysis
Tolerance = 20.0 PPM / DBE: min = -1.5, max = 50.0
Element prediction: Off
Number of isotope peaks used for i-FIT = 3

Monoisotopic Mass, Even Electron Ions
2880 formula(e) evaluated with 1 results within limits (up to 50 closest results for each mass)
Elements Used:
C: 54-54 H: 88-88 N: 0-30 O: 0-100 Na: 0-1
10--P--N
230515-6-MSY-271 5 (0.076) 1: TOF MS ES+ 1.93e+006

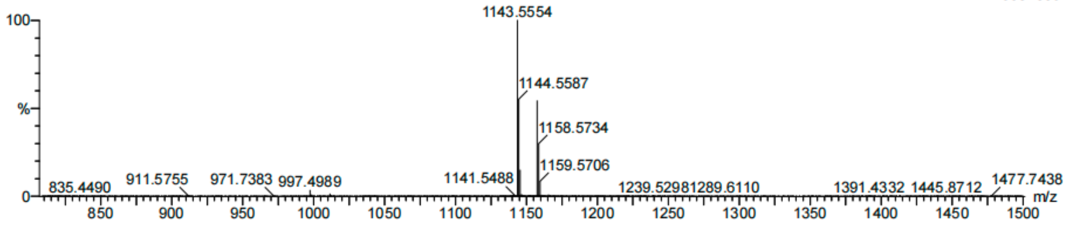

Minimum:				-1.5				
Maximum:		5.0	20.0	50.0				
Mass	Calc. Mass	mDa	PPM	DBE	i-FIT	Norm	Conf(%)	Formula
1143.5554	1143.5563	-0.9	-0.8	10.5	225.8	n/a	n/a	C54 H88 O24 Na

Figure S11. The HR-ESI-MS of compound **2**.

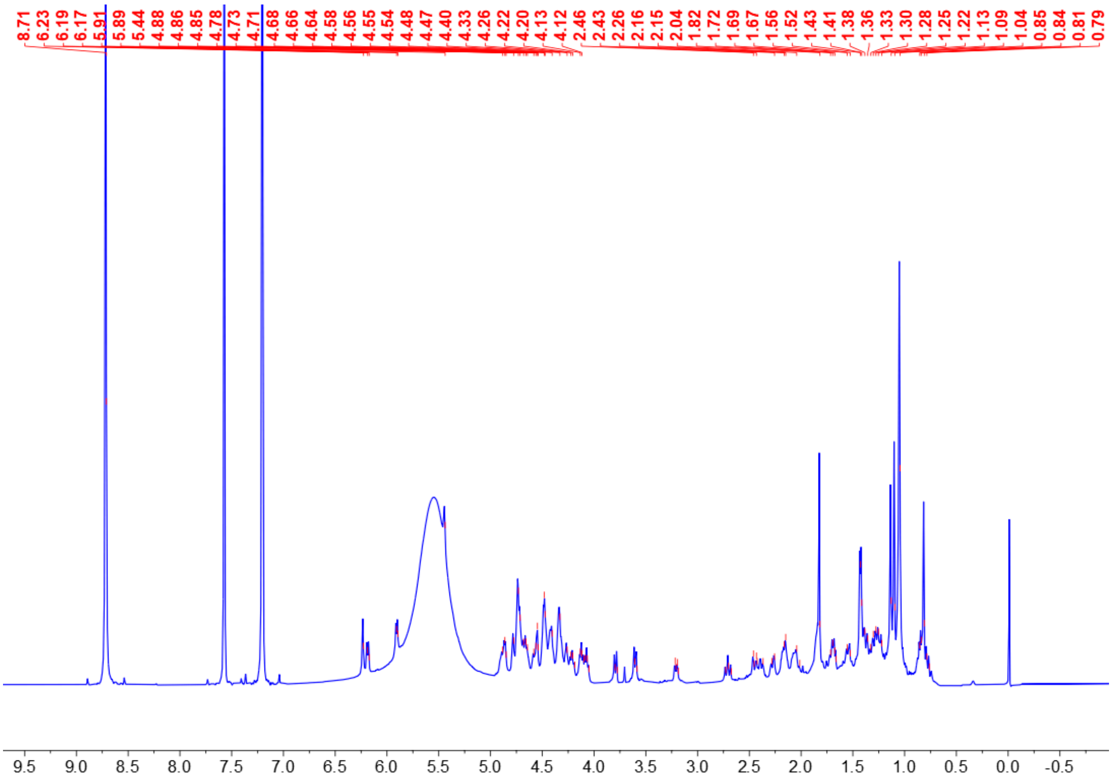

Figure S12. The ^{1}H NMR spectrum of compound **2** (600 MHz, Pyr-d_5).

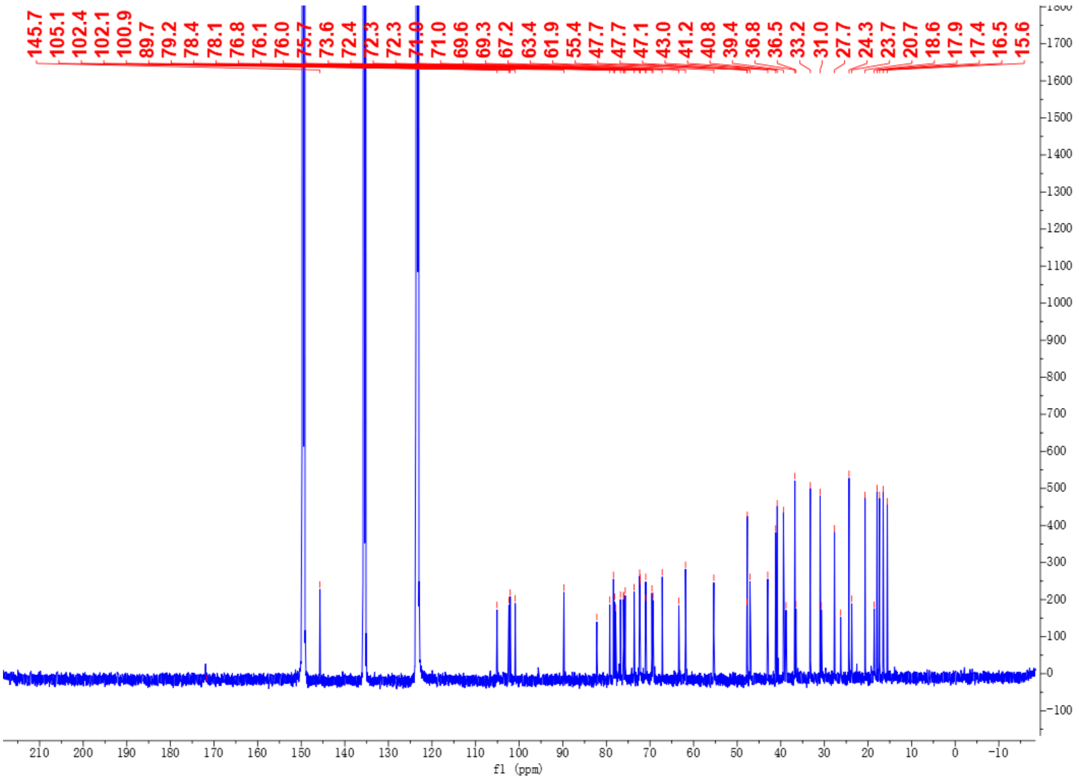

Figure S13. The ^{13}C NMR spectrum of compound **2** (151 MHz, Pyr-d_5).

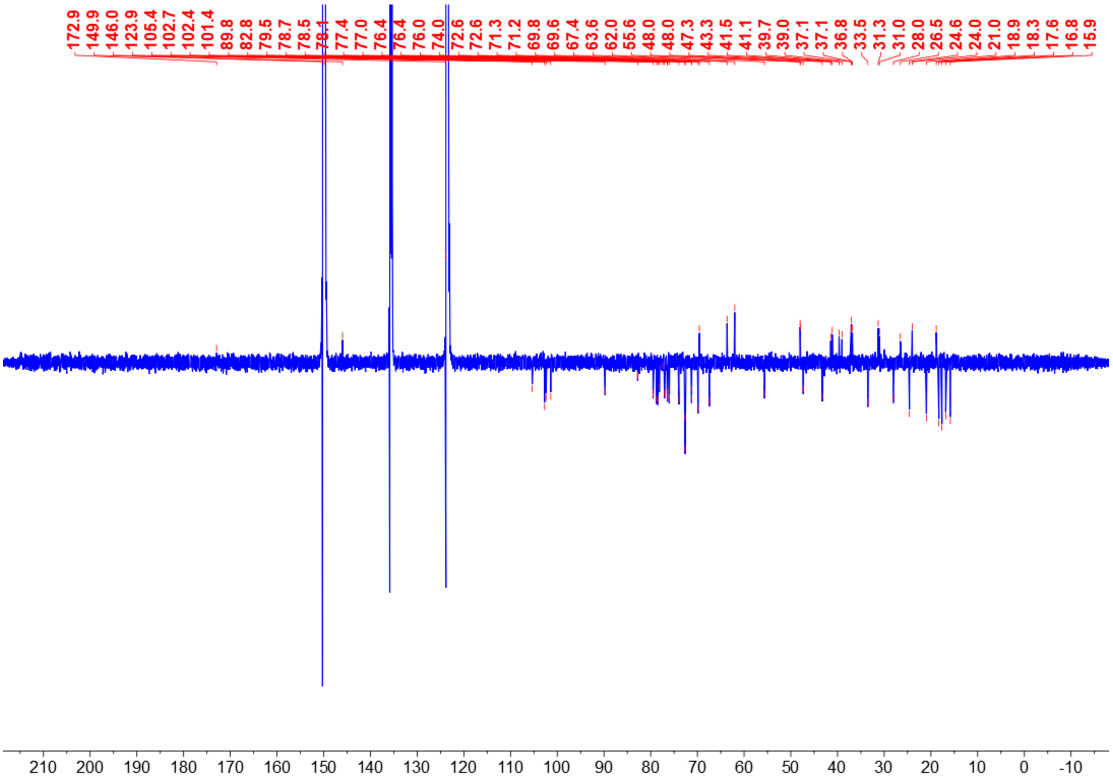

Figure S14. The DEPTQ spectrum of compound **2** (151 MHz, Pyr-d_5).

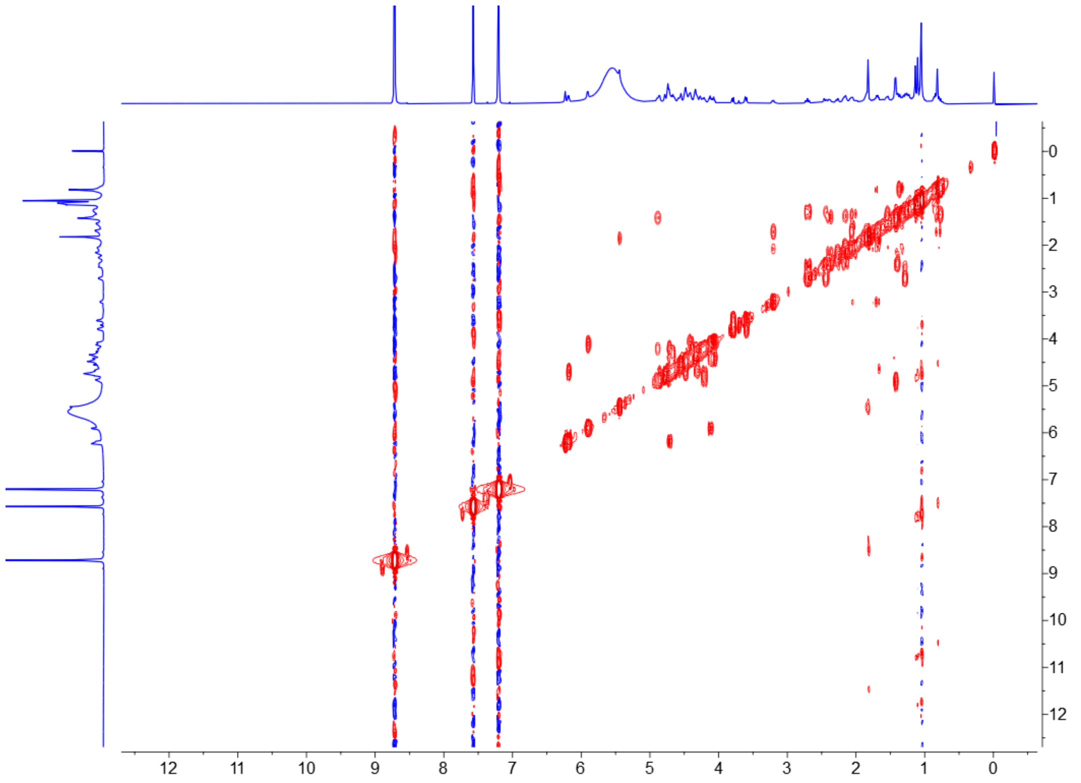

Figure S15. The ^{1}H-^{1}H COSY spectrum of compound **2** (600 MHz, Pyr-d_5).

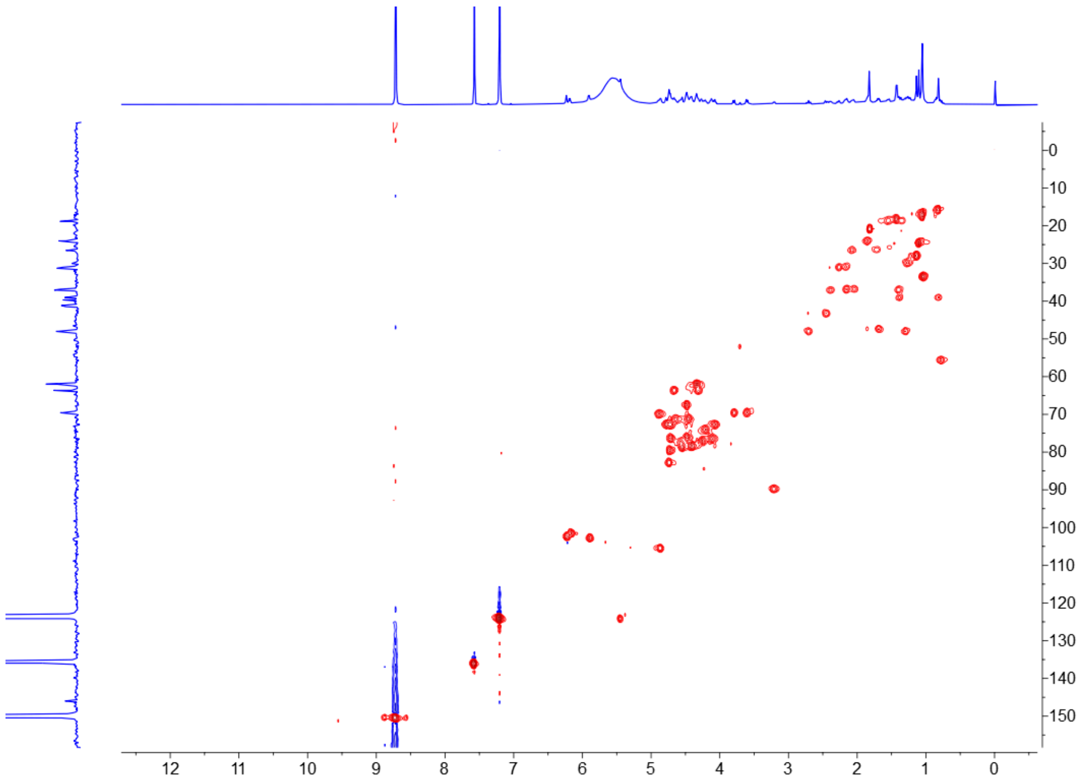

Figure S16. The HSQC spectrum of compound **2** (600/151 MHz, Pyr-d_5).

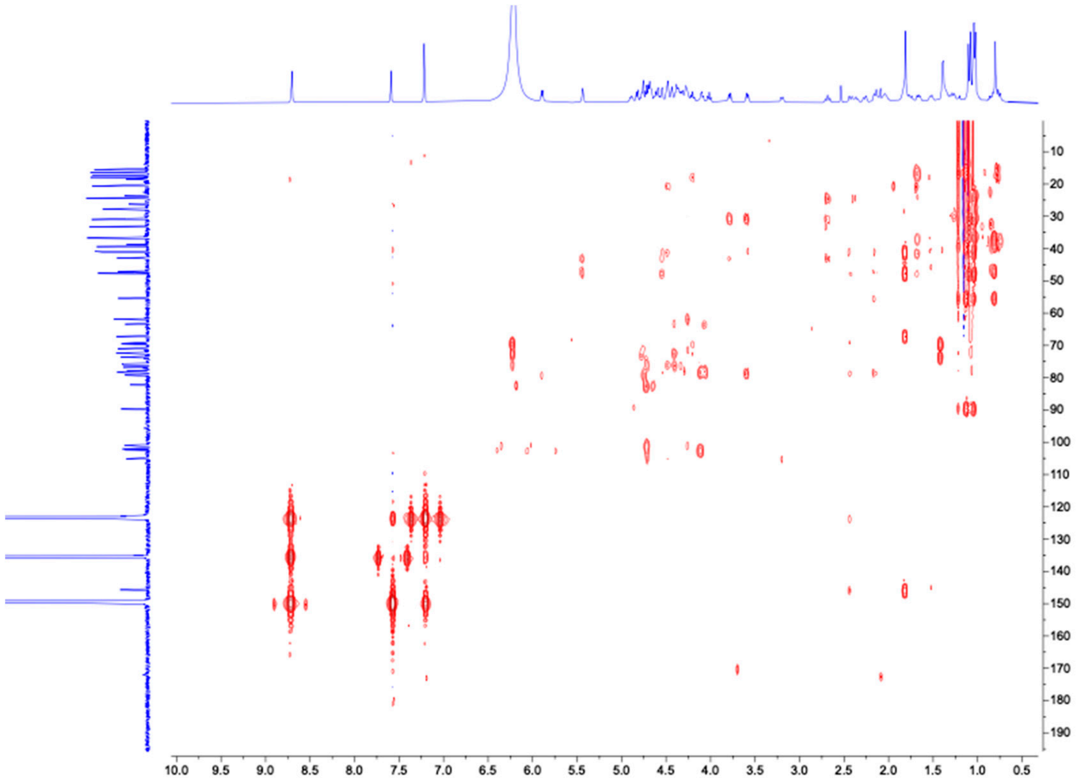

Figure S17. The HMBC spectrum of compound **2** (600/151 MHz, Pyr-d_5).

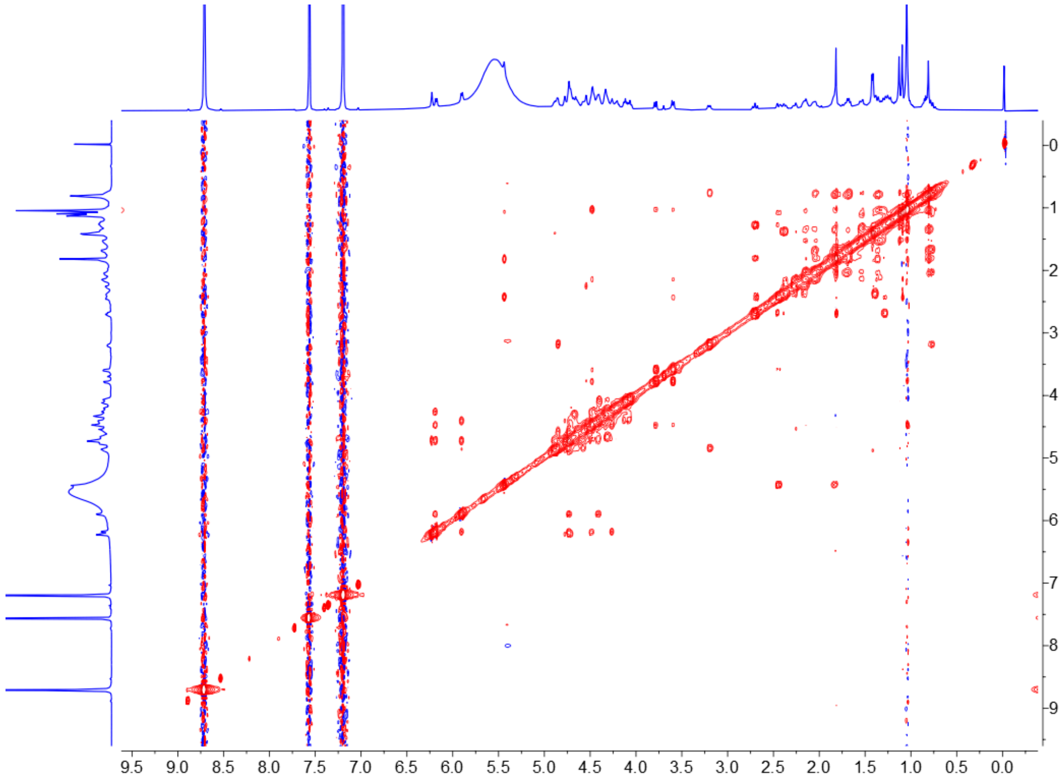

Figure S18. The NOESY spectrum of compound **2** (600 MHz, Pyr-d_5).

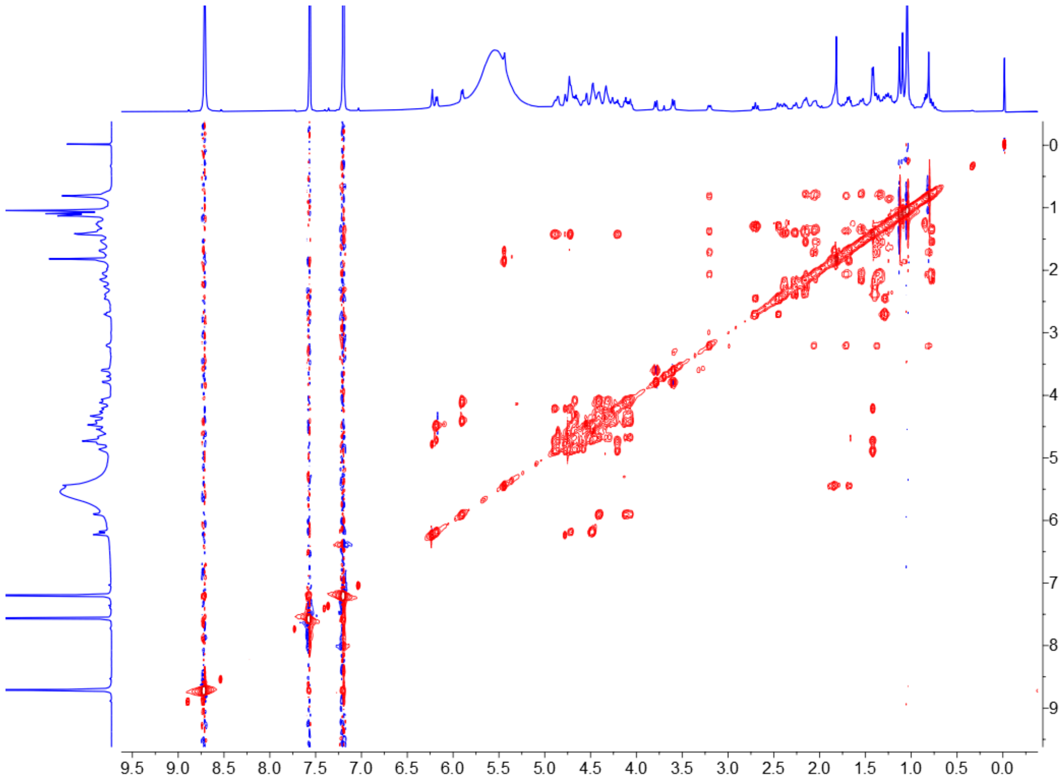

Figure S19. The TOCSY spectrum of compound **2** (600 MHz, Pyr-d_5).

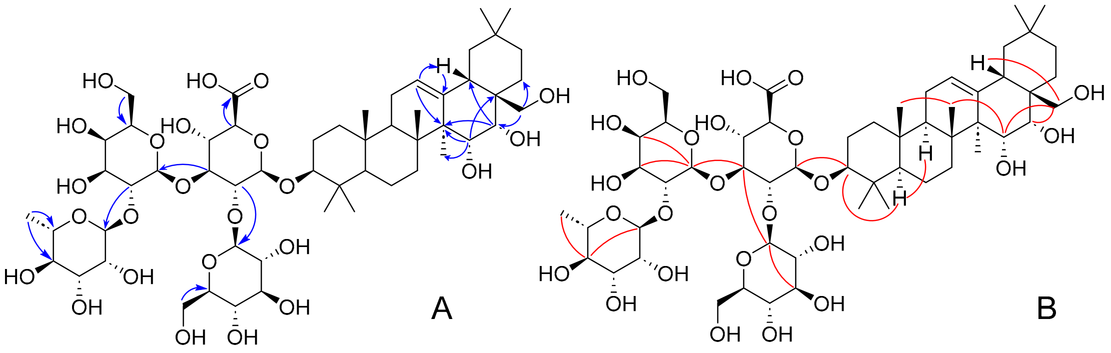

Figure S20. Key HMBC (A) and NOESY (B) correlations of compound **2**.

3. Spectroscopic data for compound **3**

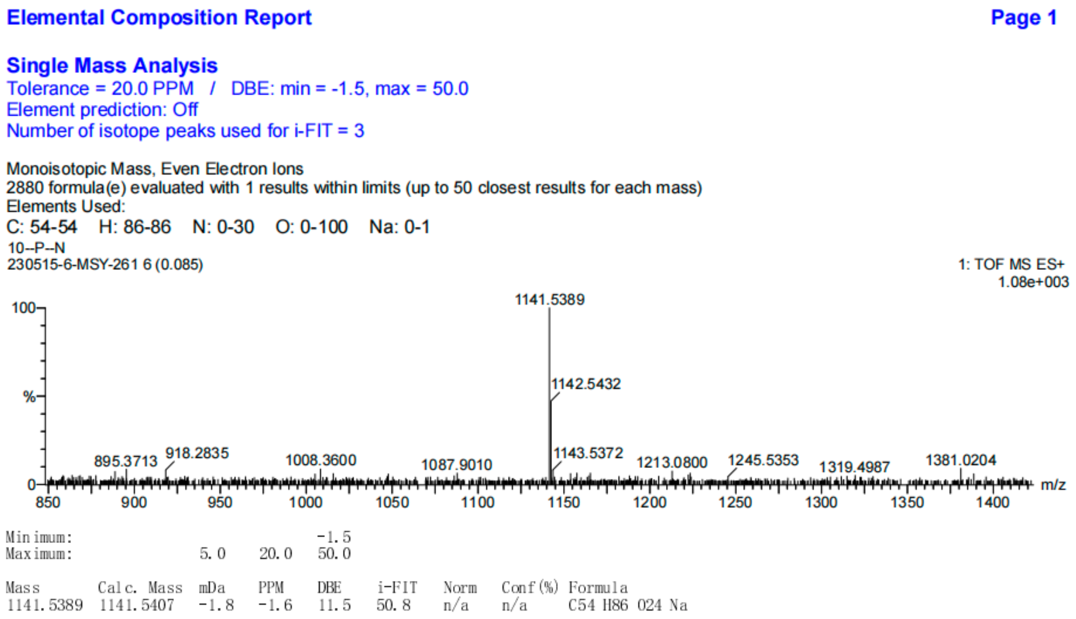

Figure S21. The HR-ESI-MS of compound **3**.

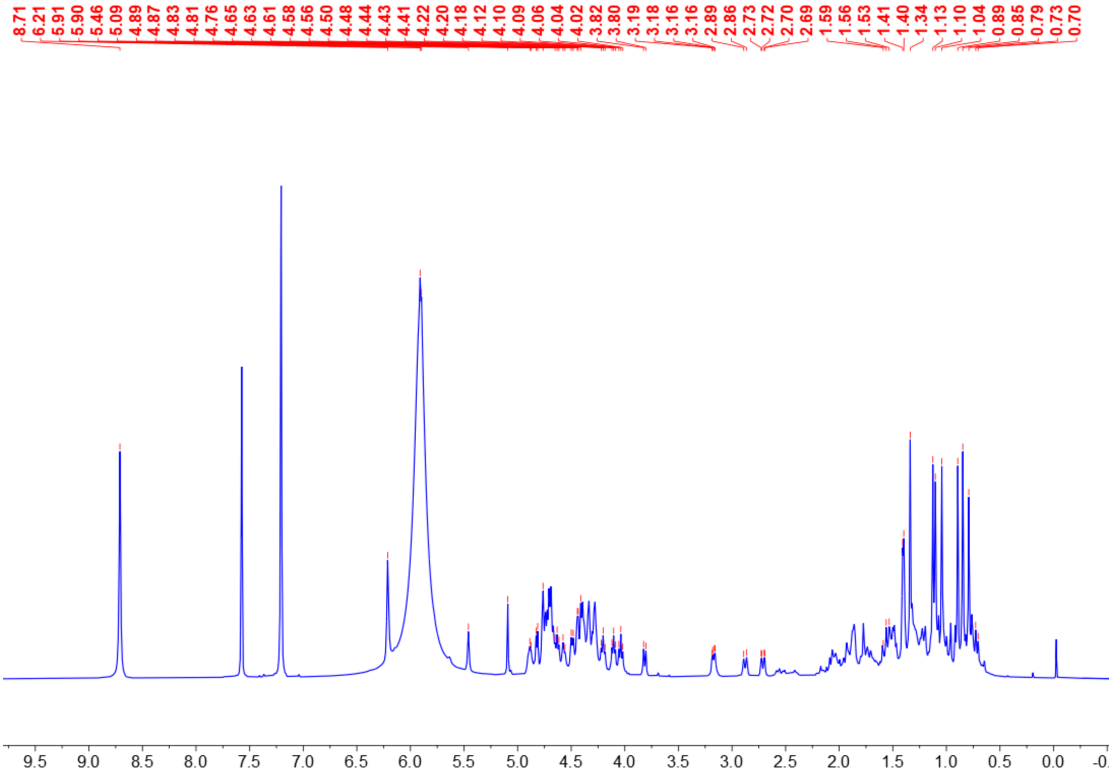

Figure S22. The ^{1}H NMR spectrum of compound **3** (600 MHz, Pyr-d_5).

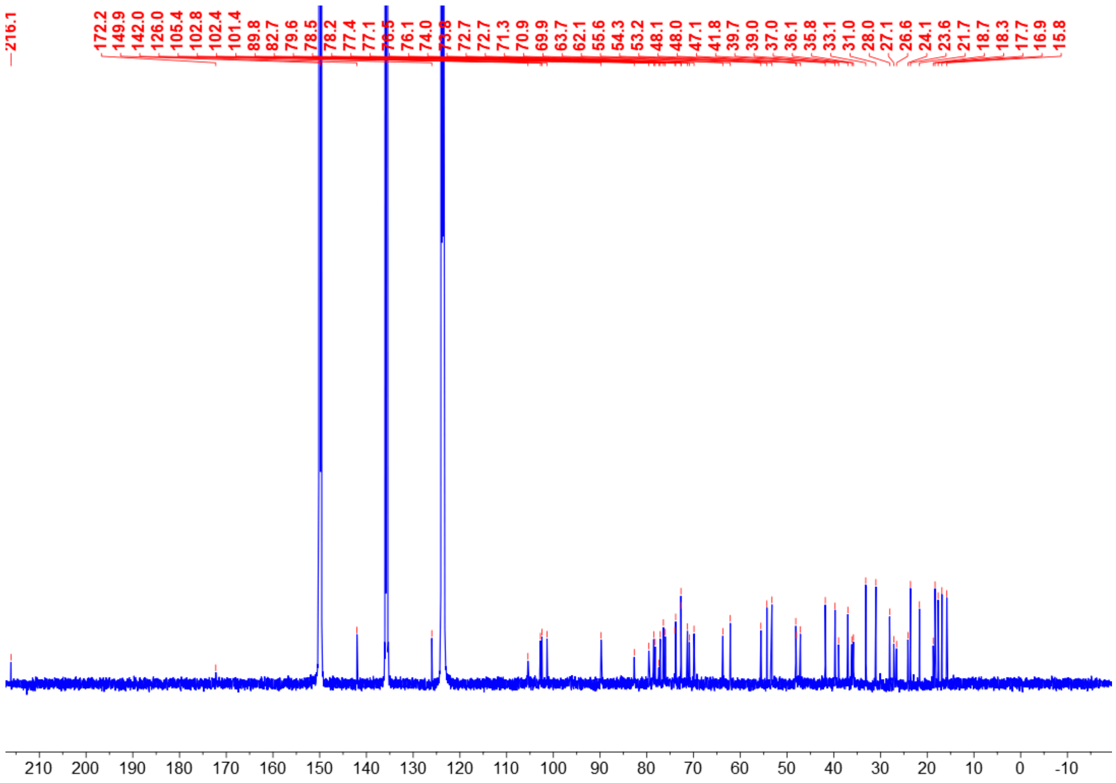

Figure S23. The ^{13}C NMR spectrum of compound **3** (151 MHz, Pyr-d_5).

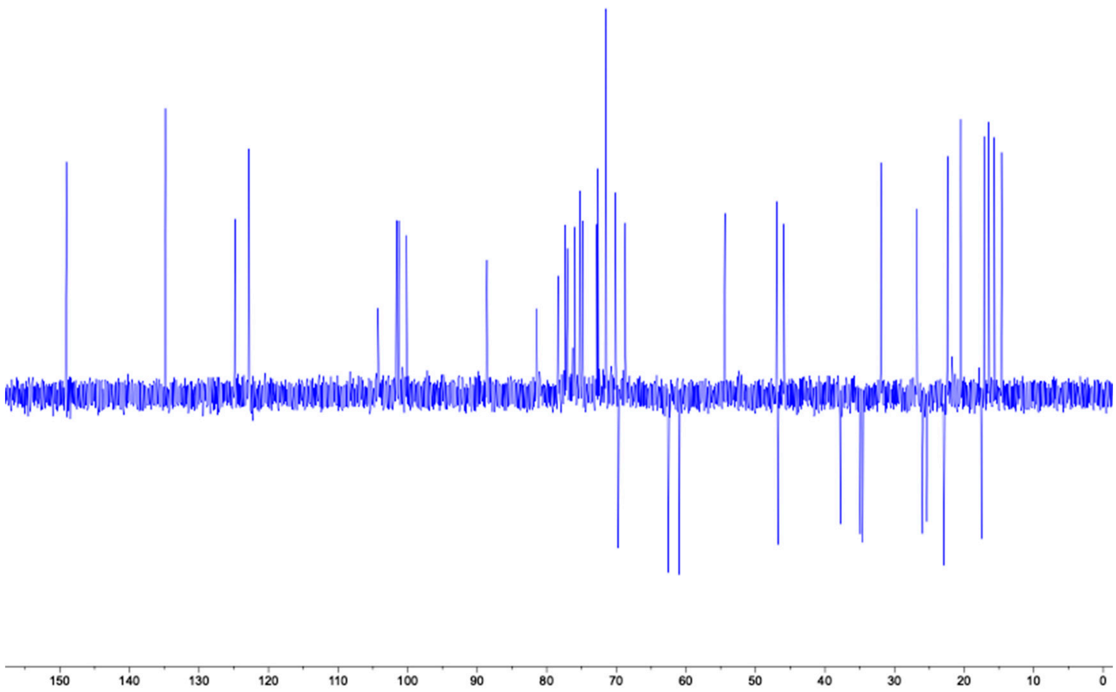

Figure S24. The DEPT 135 spectrum of compound **3** (151 MHz, Pyr-d_5).

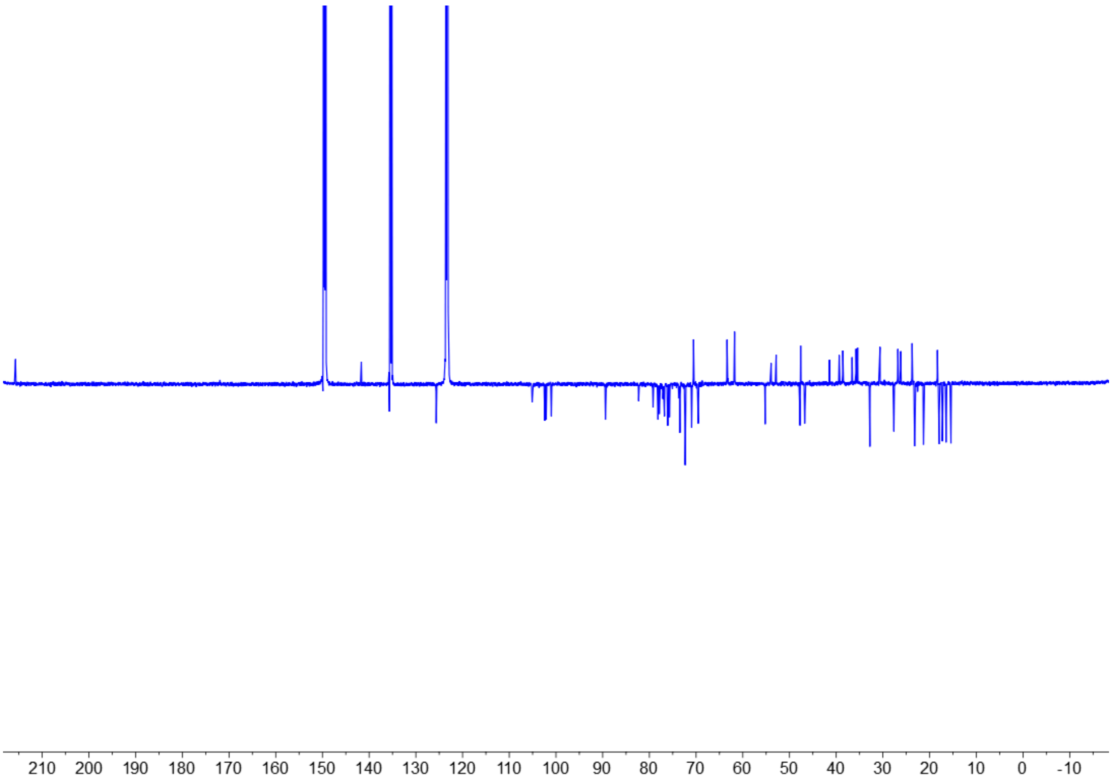

Figure S25. The DEPTQ spectrum of compound **3** (151 MHz, Pyr-d_5).

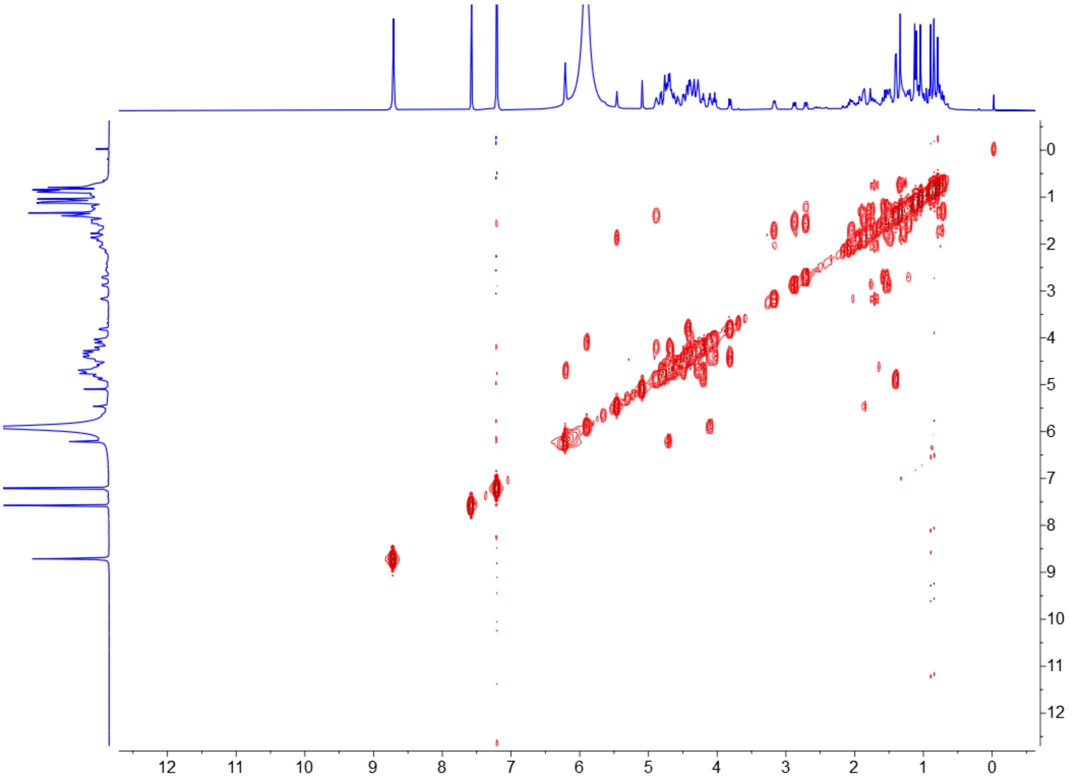

Figure S26. The ^1H-^1H COSY spectrum of compound **3** (600 MHz, Pyr-d_5).

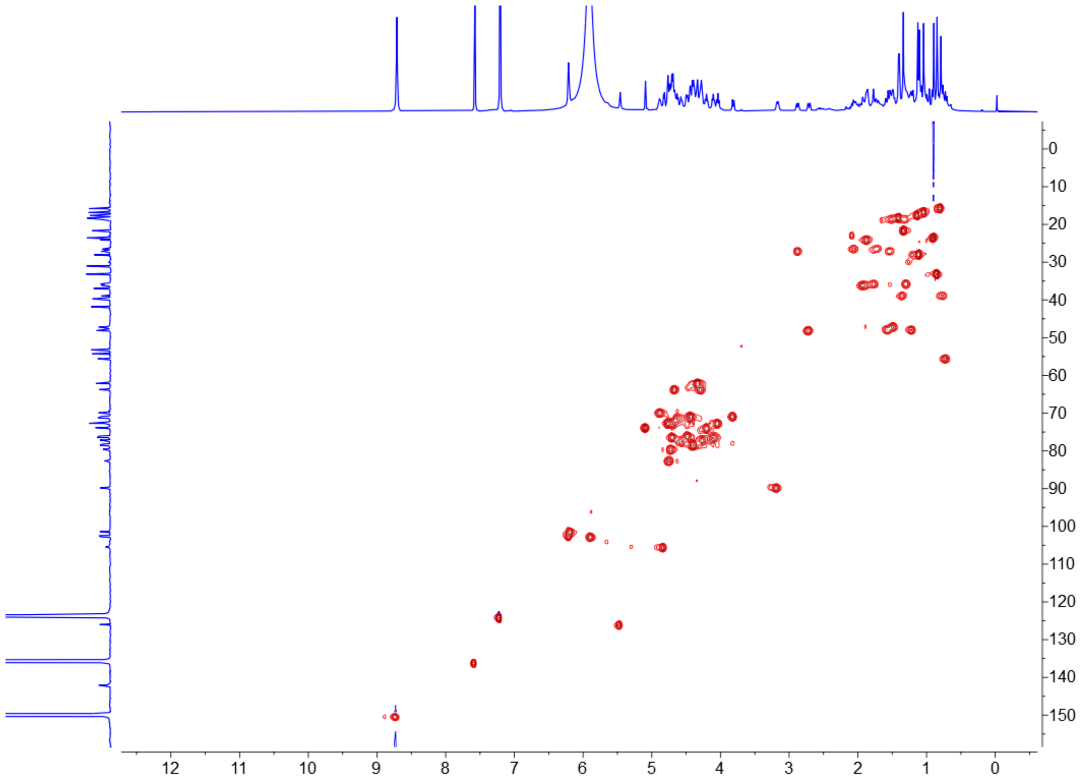

Figure S27. The HSQC spectrum of compound **3** (600/151 MHz, Pyr-d_5).

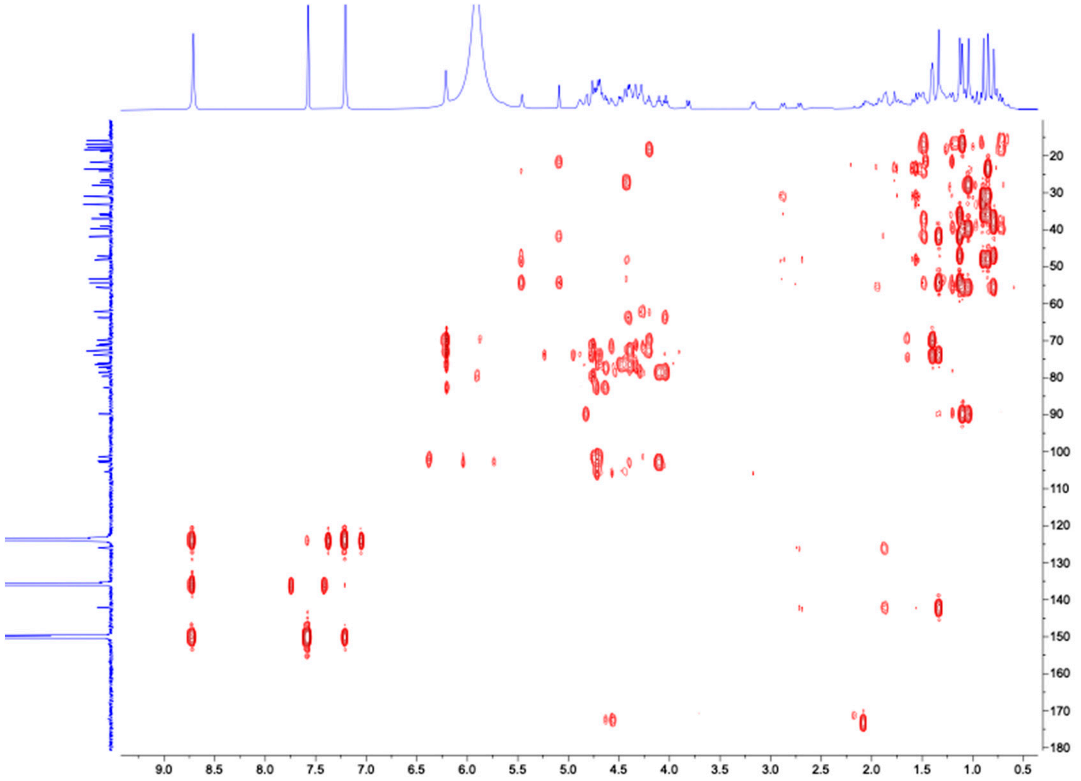

Figure S28. The HMBC spectrum of compound **3** (600/151 MHz, Pyr-d_5).

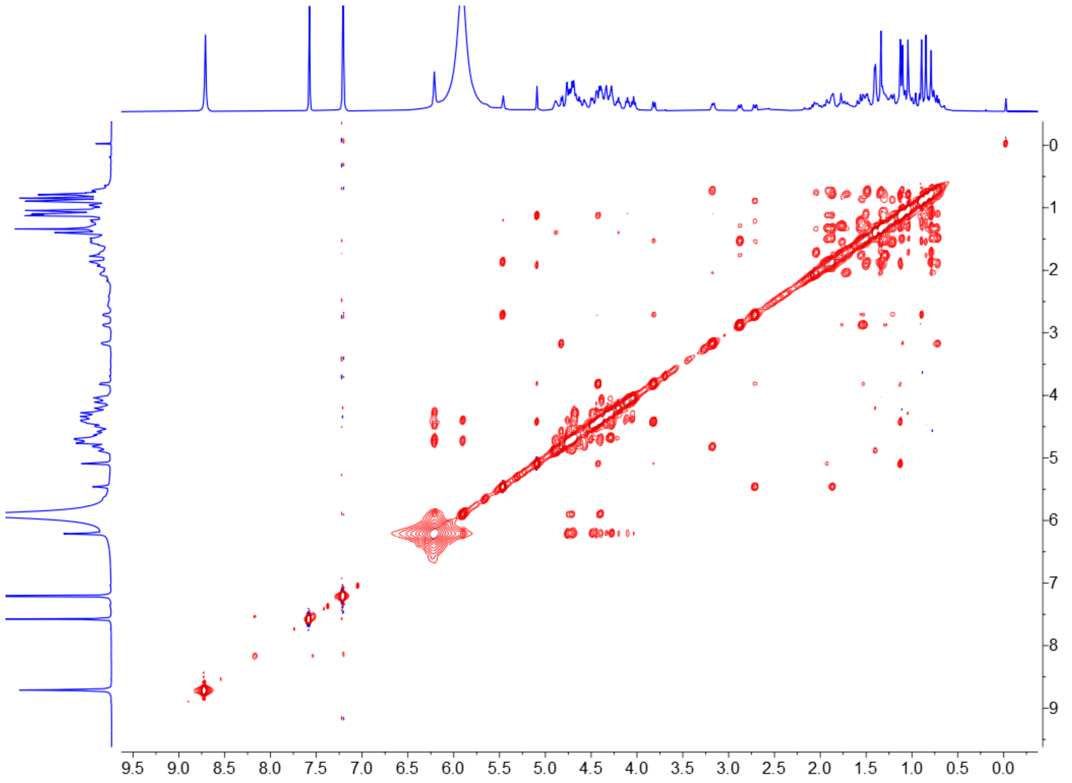

Figure S29. The NOESY spectrum of compound **3** (600 MHz, Pyr-d_5).

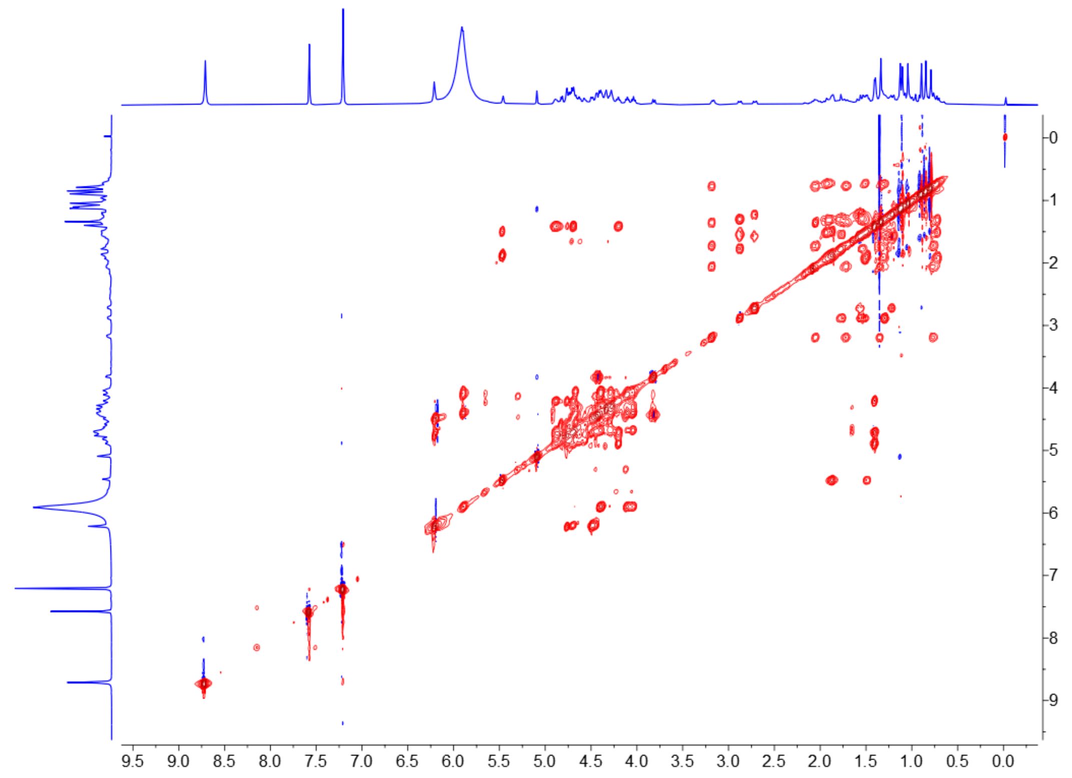

Figure S30. The TOCSY spectrum of compound **3** (600 MHz, Pyr-d_5).

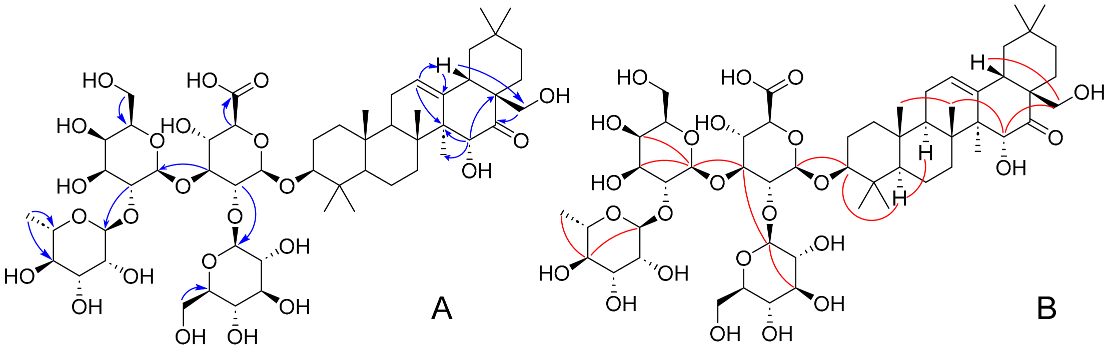

Figure S31. Key HMBC (A) and NOESY (B) correlations of compound **3**.

4. Spectroscopic data for compound **4**

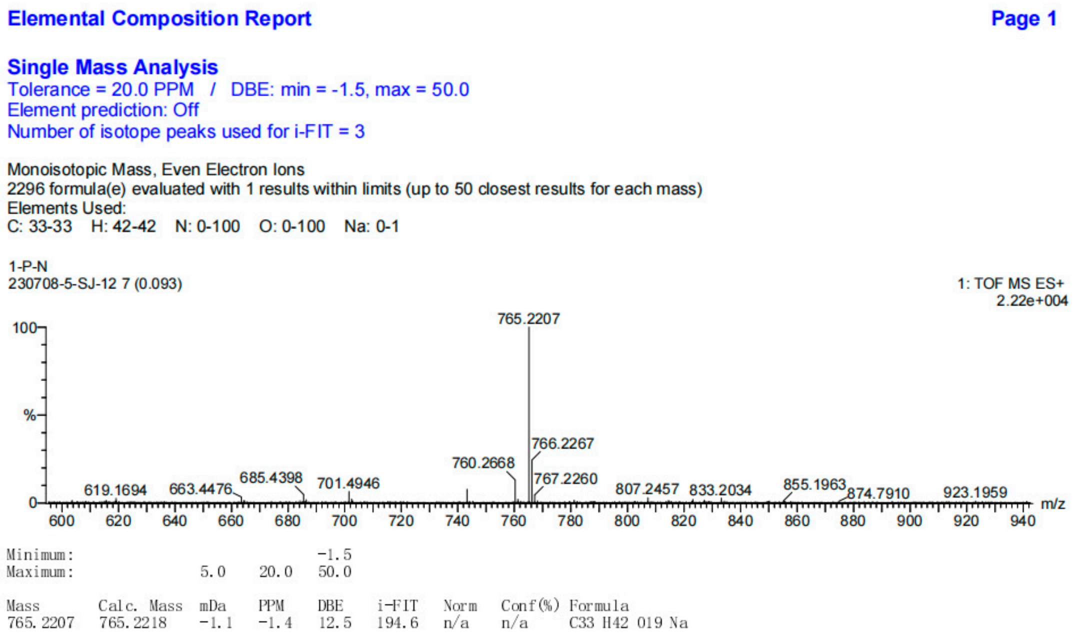

Figure S32. The HR-ESI-MS of compound **4**.

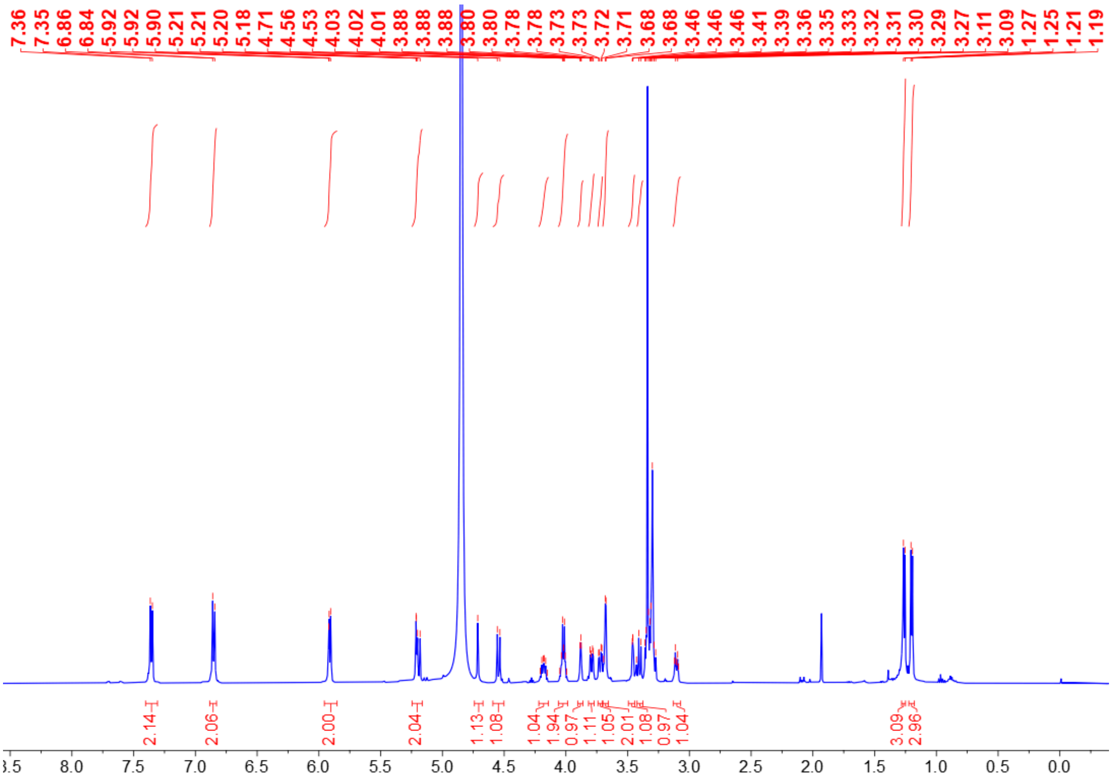

Figure S33. The ^{1}H NMR spectrum of compound **4** (600 MHz, CD_3OD).

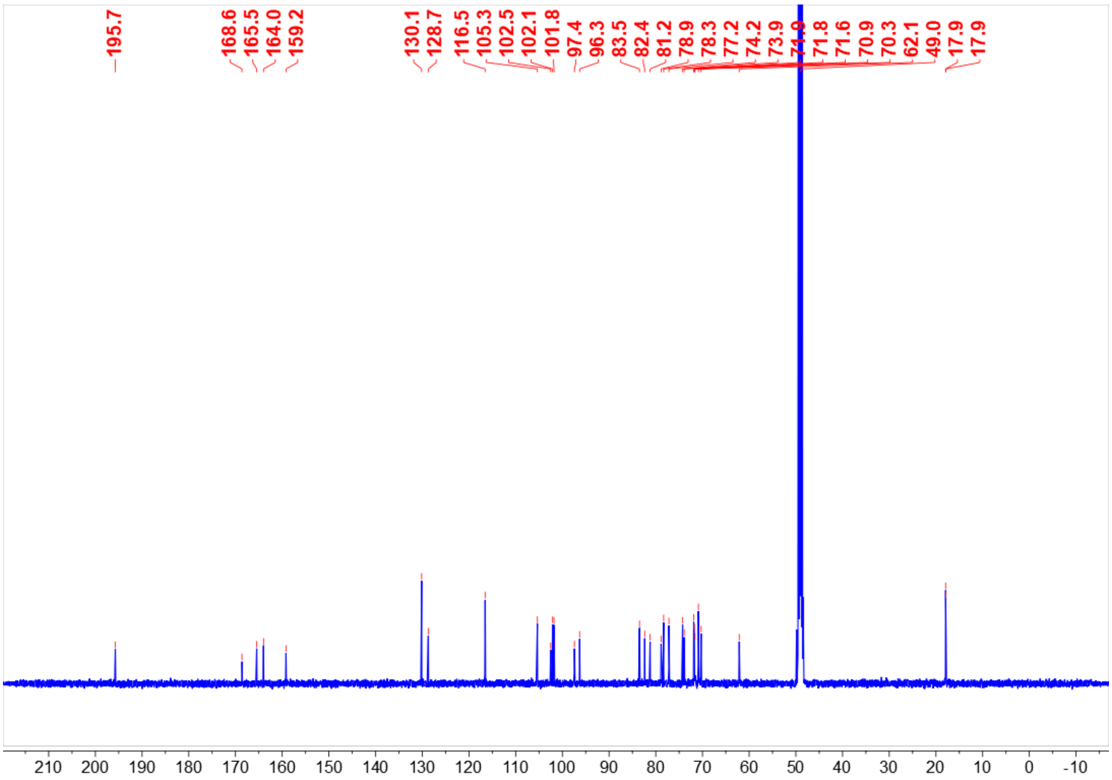

Figure S34. The ^{13}C NMR spectrum of compound **4** (151 MHz, CD_3OD).

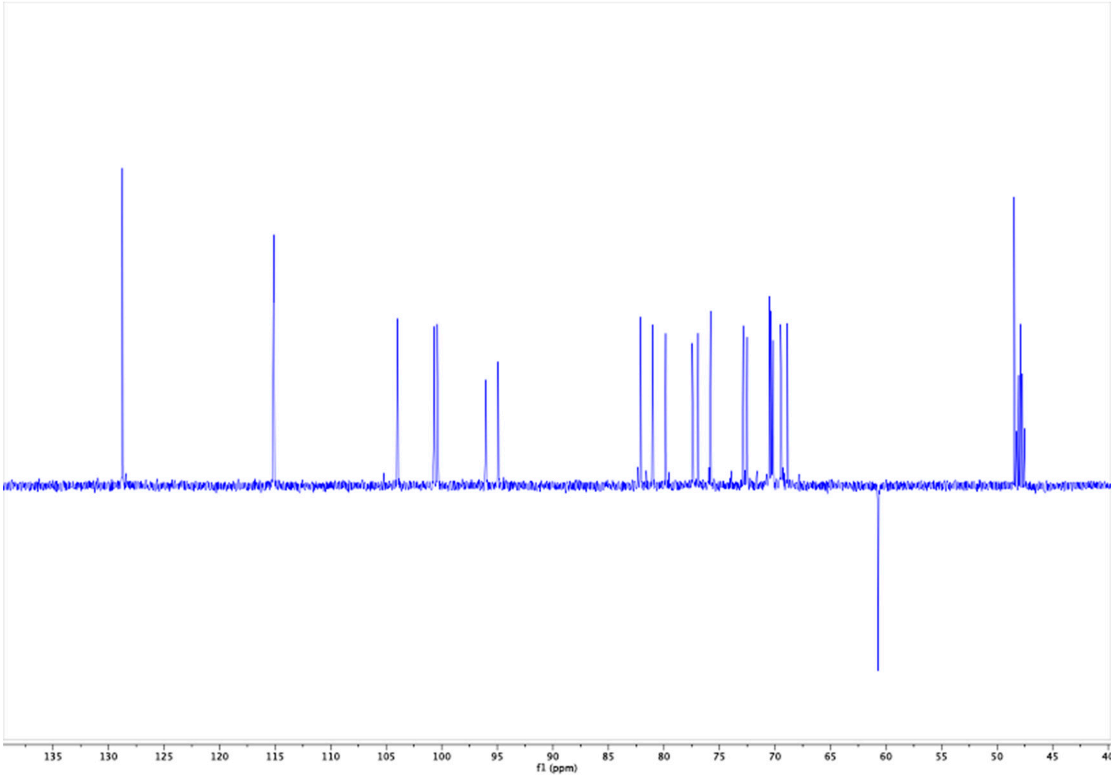

Figure S35. The DEPT 135 spectrum of compound **4** (151 MHz, CD_3OD).

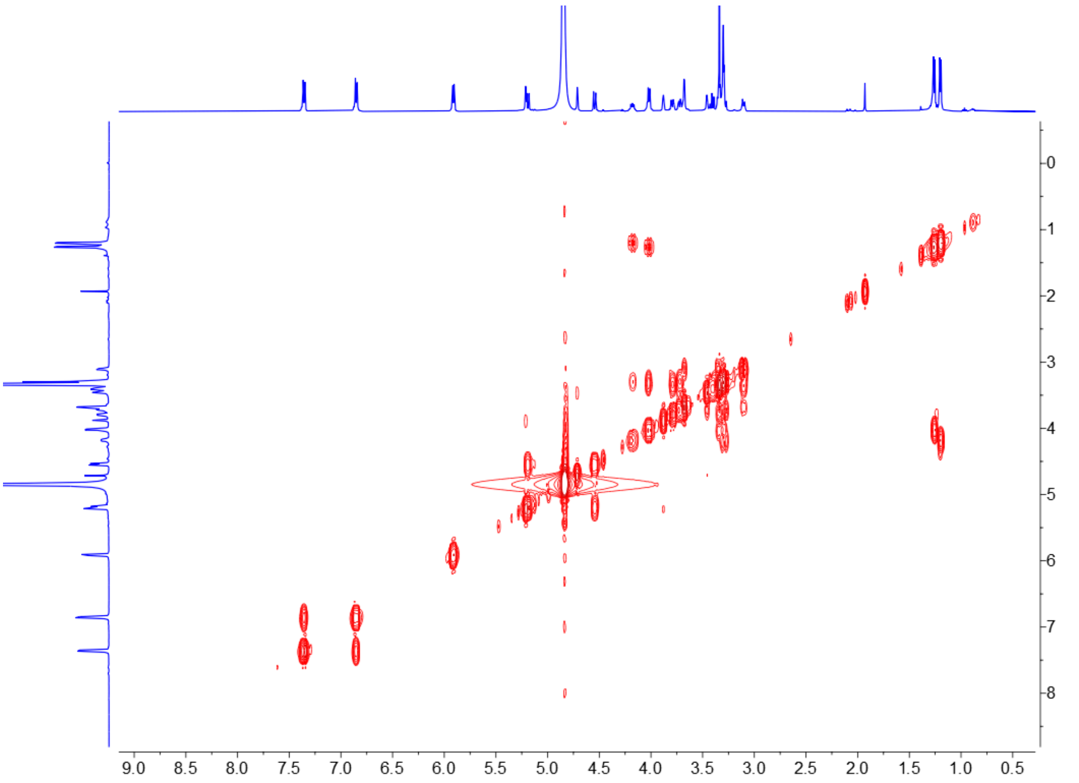

Figure S36. The ^{1}H-^{1}H COSY spectrum of compound **4** (600 MHz, CD_3OD).

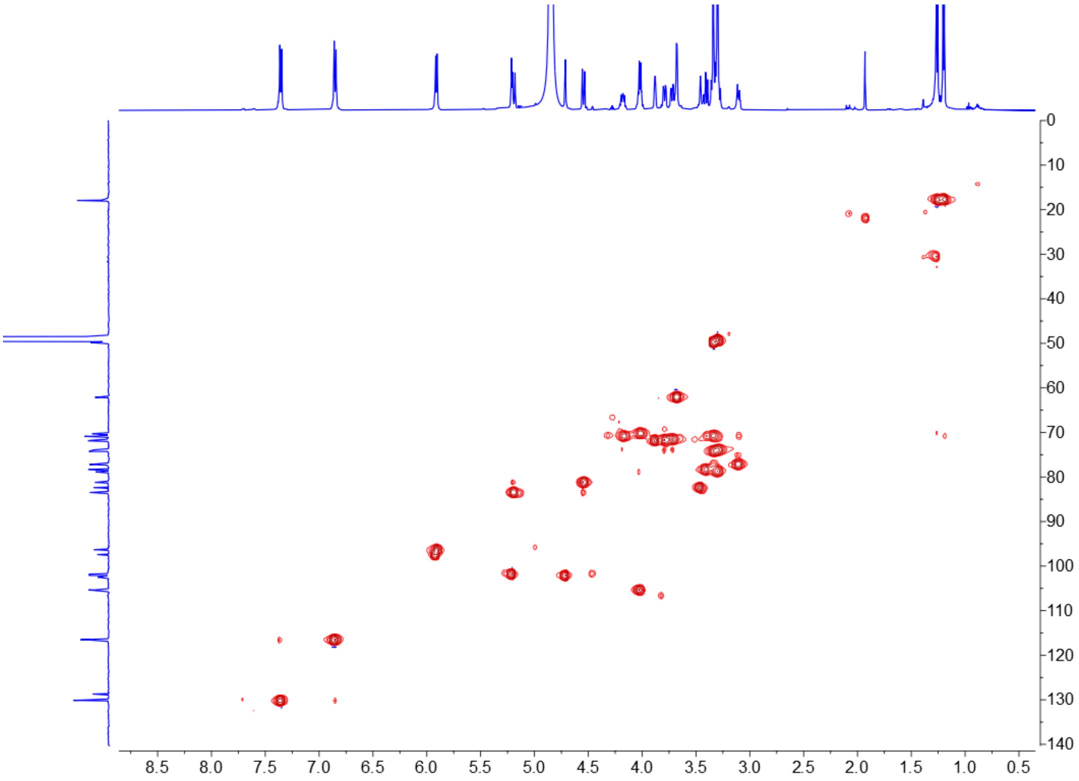

Figure S37. The HSQC spectrum of compound **4** (600/151 MHz, CD_3OD).

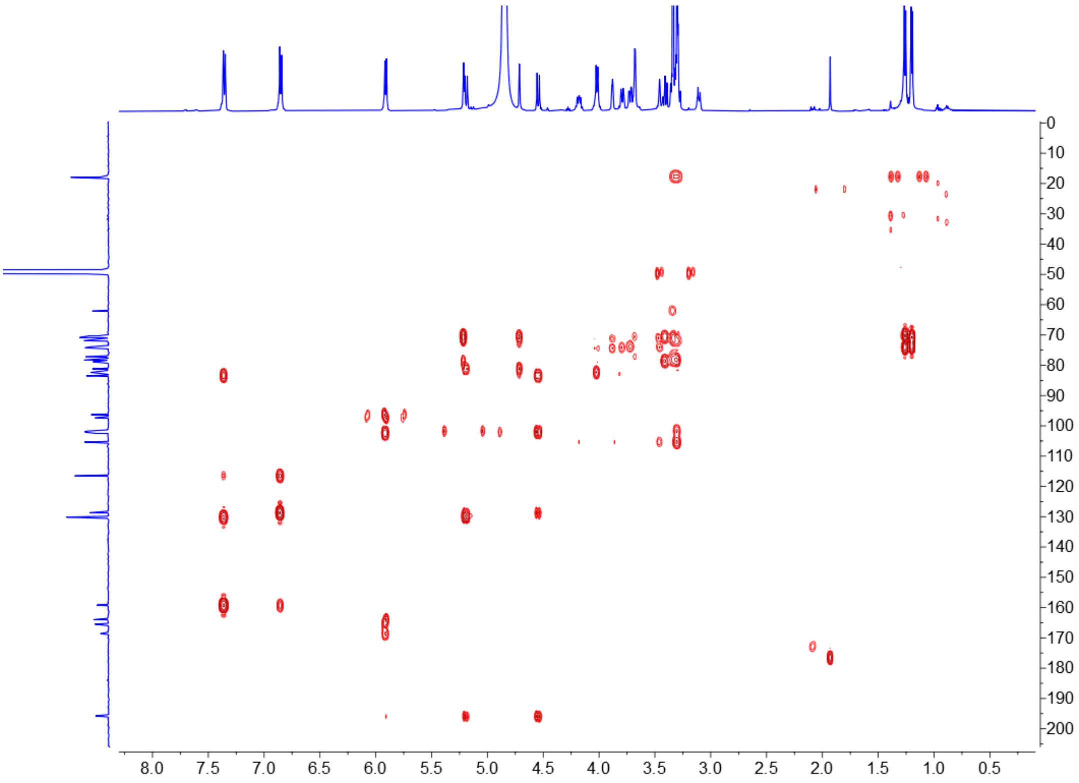

Figure S38. The HMBC spectrum of compound **4** (600/151 MHz, CD_3OD).

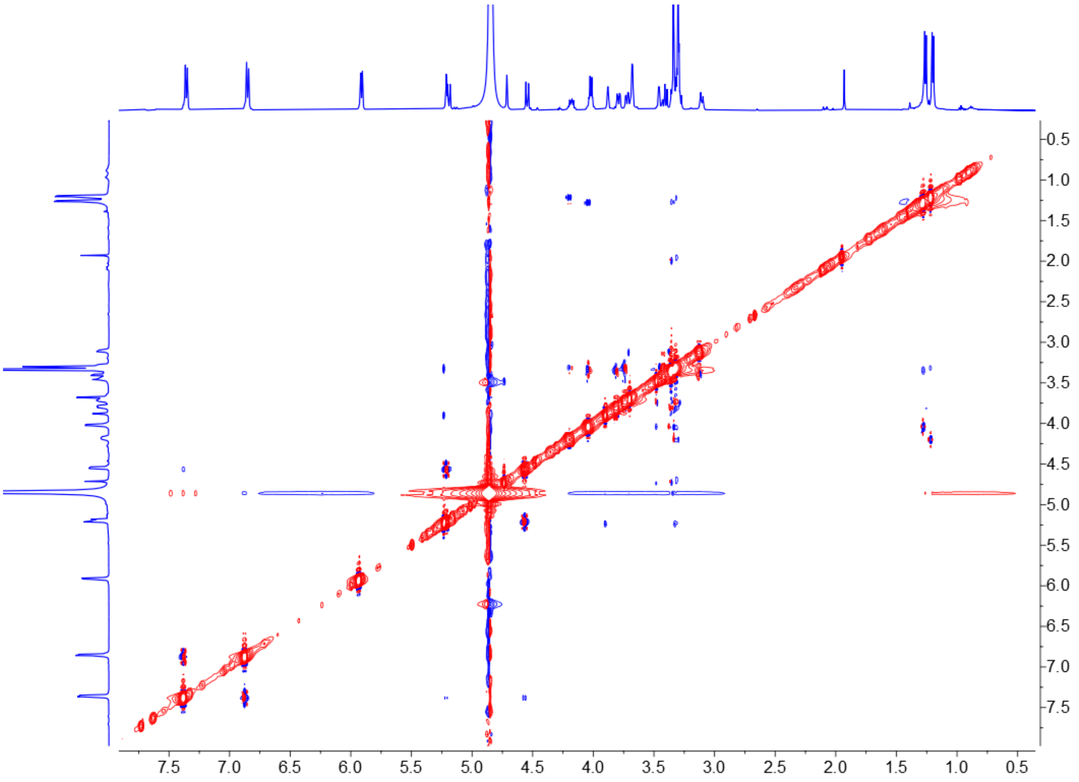

Figure S39. The NOESY spectrum of compound **4** (600 MHz, CD_3OD).

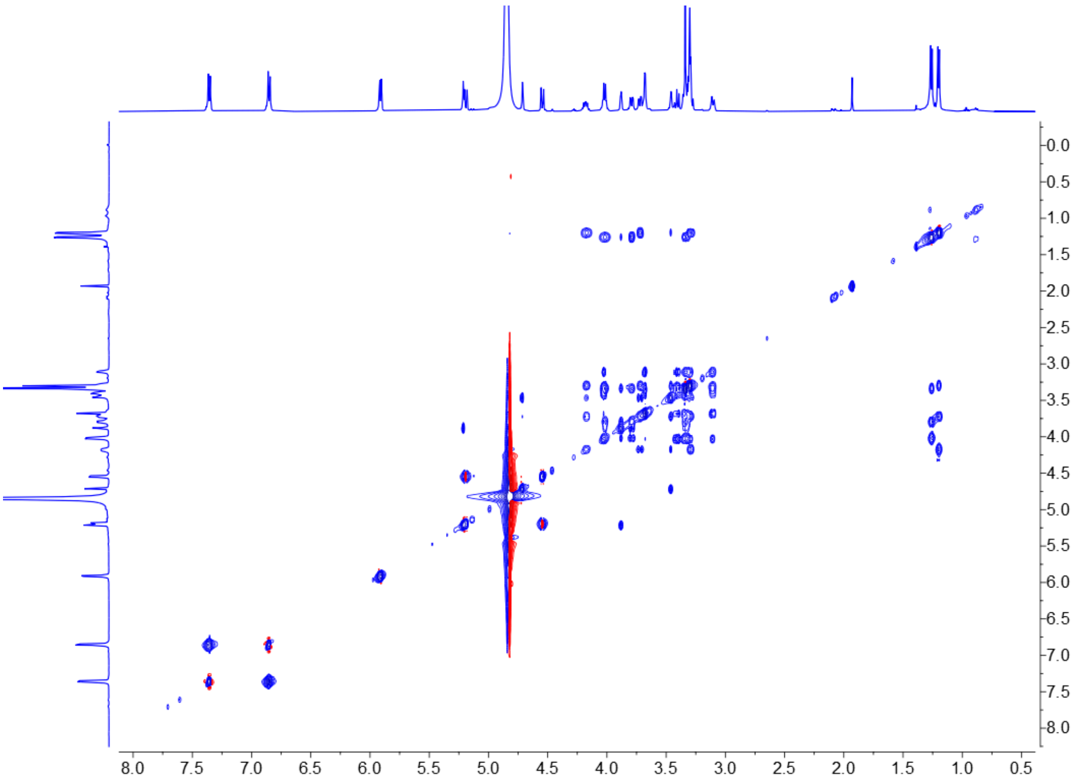

Figure S40. The TOCSY spectrum of compound **4** (600 MHz, CD_3OD).

5. Spectroscopic data for compound **5**

Elemental Composition Report **Page 1**

Single Mass Analysis
Tolerance = 20.0 PPM / DBE: min = -1.5, max = 50.0
Element prediction: Off
Number of isotope peaks used for i-FIT = 3

Monoisotopic Mass, Even Electron Ions
2559 formula(e) evaluated with 1 results within limits (up to 50 closest results for each mass)
Elements Used:
C: 35-35 H: 44-44 N: 0-100 O: 0-100 Na: 0-1
8–P-N
230705-9-MSY-282-3 5 (0.076) 1: TOF MS ES+ 7.26e+003

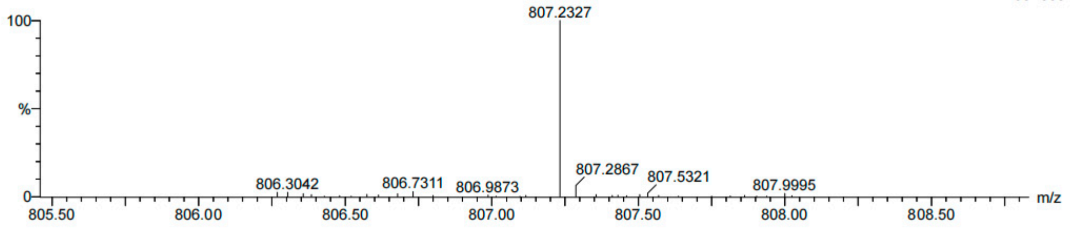

Minimum:				-1.5				
Maximum:		5.0	20.0	50.0				
Mass	Calc. Mass	mDa	PPM	DBE	i-FIT	Norm	Conf(%)	Formula
807.2327	807.2324	0.3	0.4	13.5	157.0	n/a	n/a	C35 H44 O20 Na

Figure S41. The HR-ESI-MS of compound **5**.

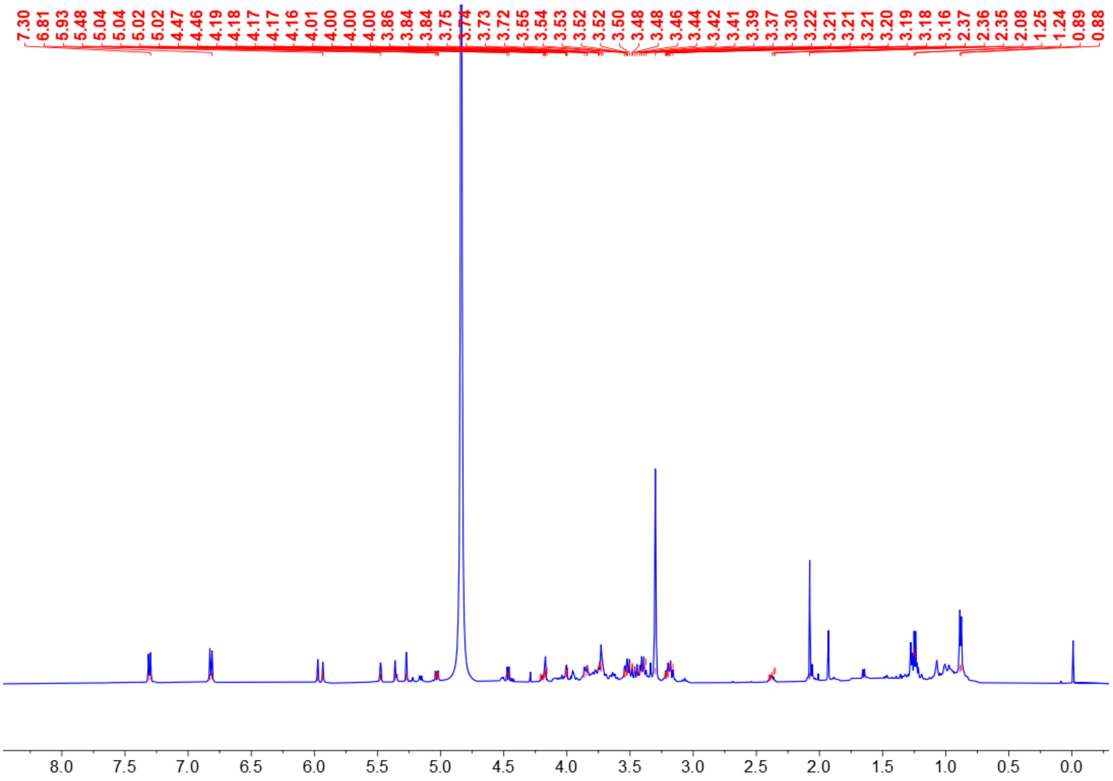

Figure S42. The ^{1}H NMR spectrum of compound **5** (600 MHz, CD_3OD).

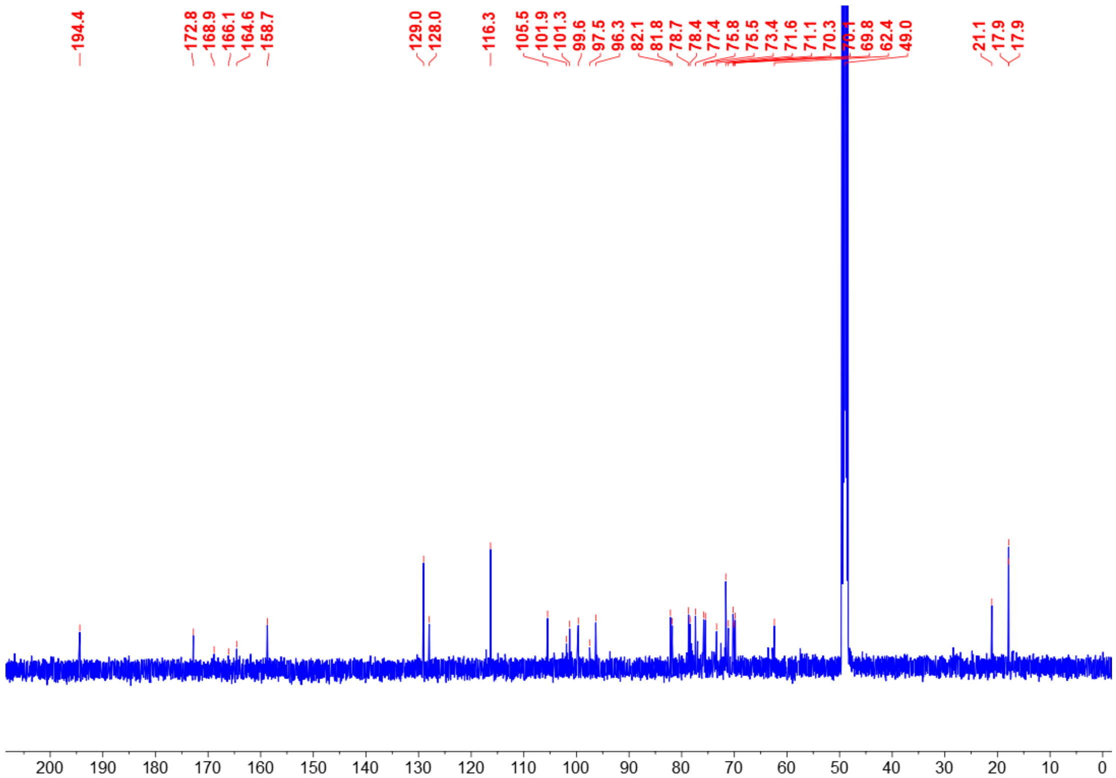

Figure S43. The ^{13}C NMR spectrum of compound **5** (151 MHz, CD_3OD).

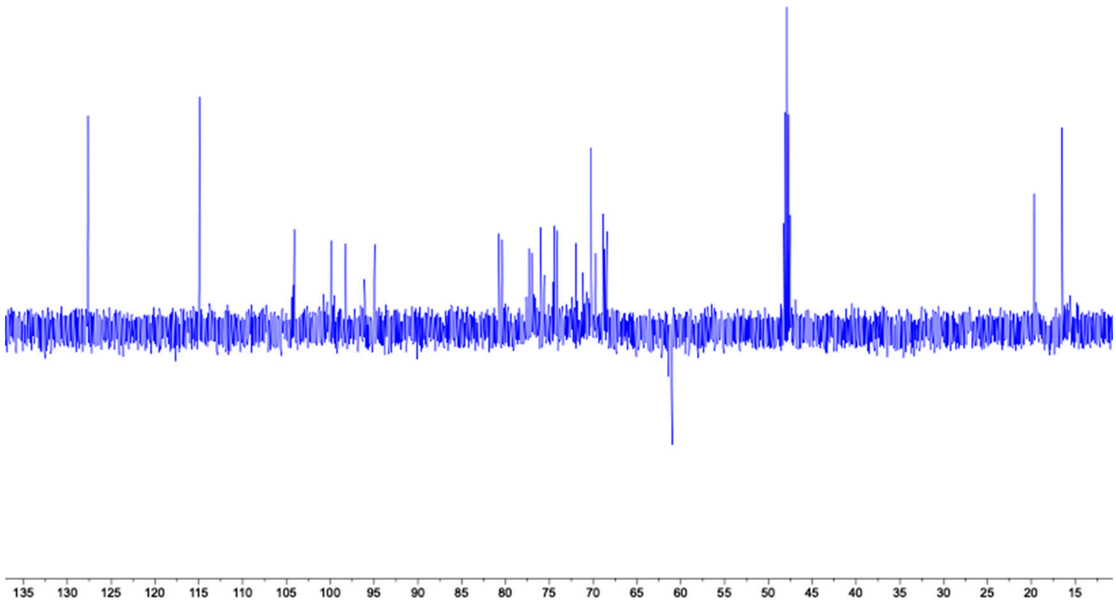

Figure S44. The DEPT 135 spectrum of compound **5** (151 MHz, CD_3OD).

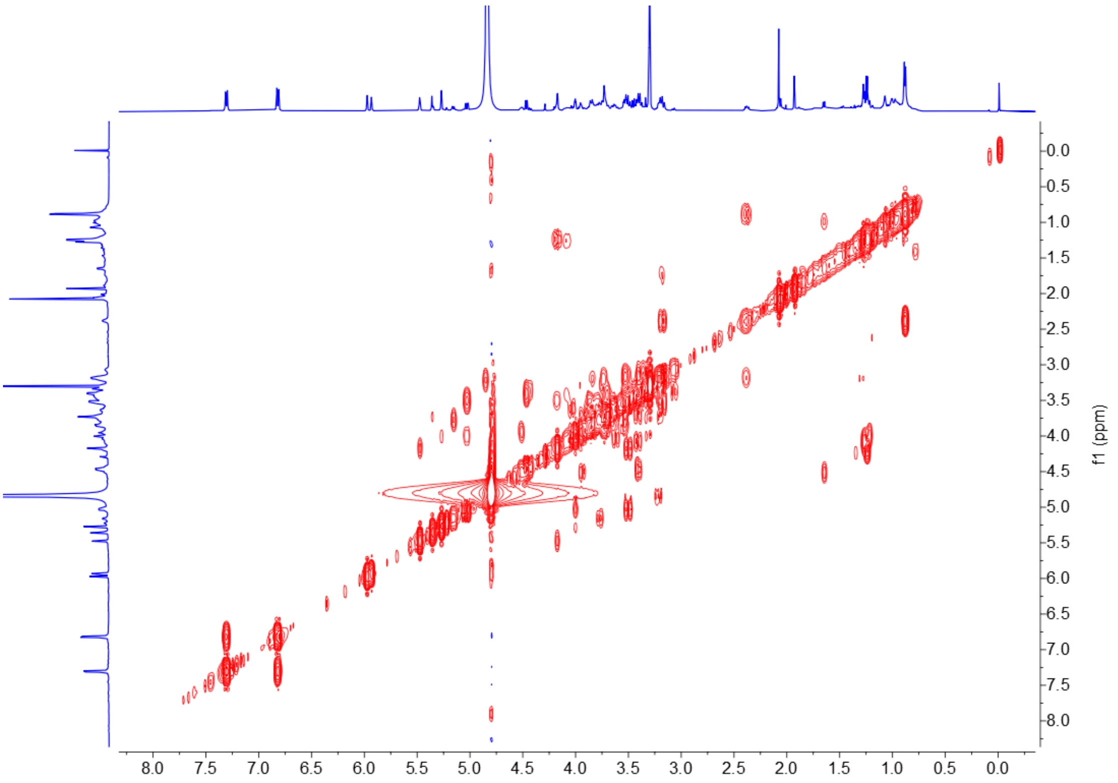

Figure S45. The ^{1}H-^{1}H COSY spectrum of compound **5** (600 MHz, CD_3OD).

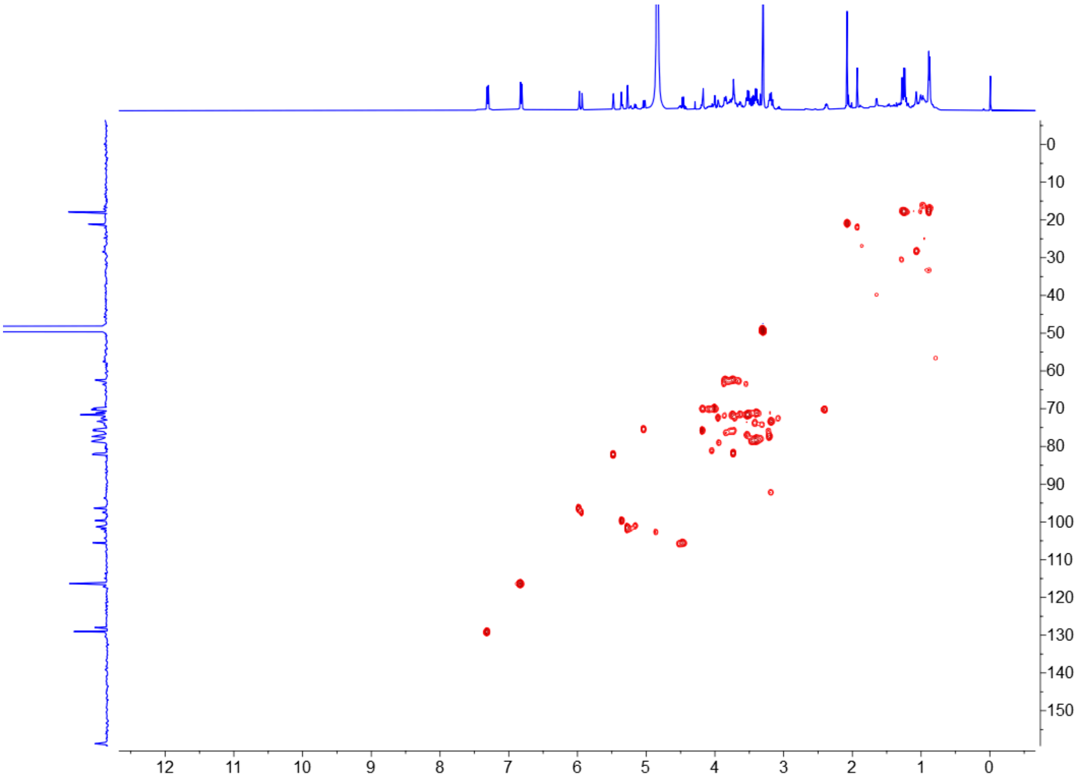

Figure S46. The HSQC spectrum of compound **5** (600/151 MHz, CD_3OD).

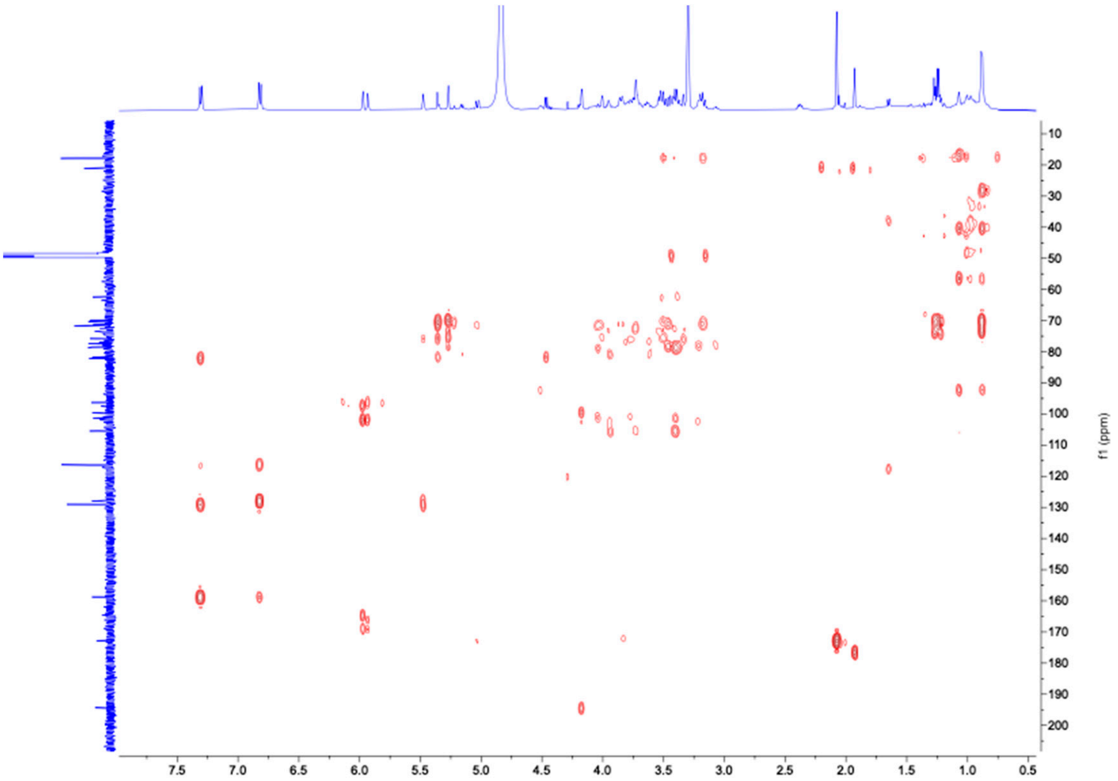

Figure S47. The HMBC spectrum of compound **5** (600/151 MHz, CD_3OD).

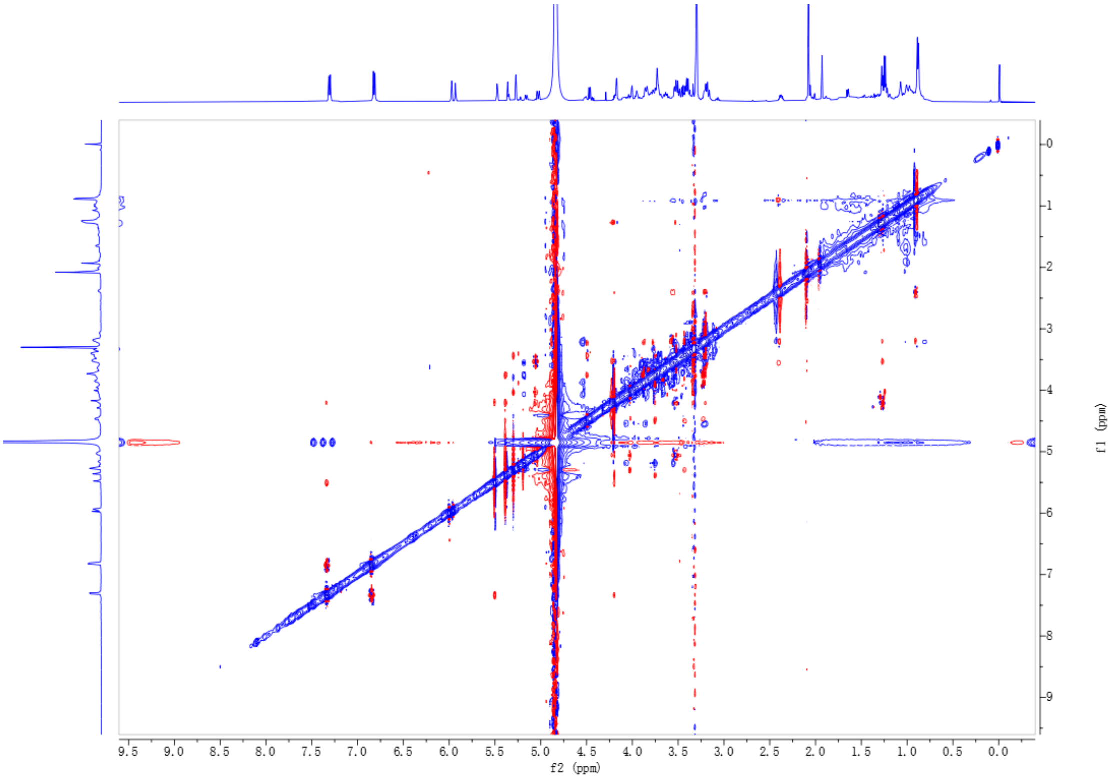

Figure S48. The NOESY spectrum of compound **5** (600 MHz, CD_3OD).

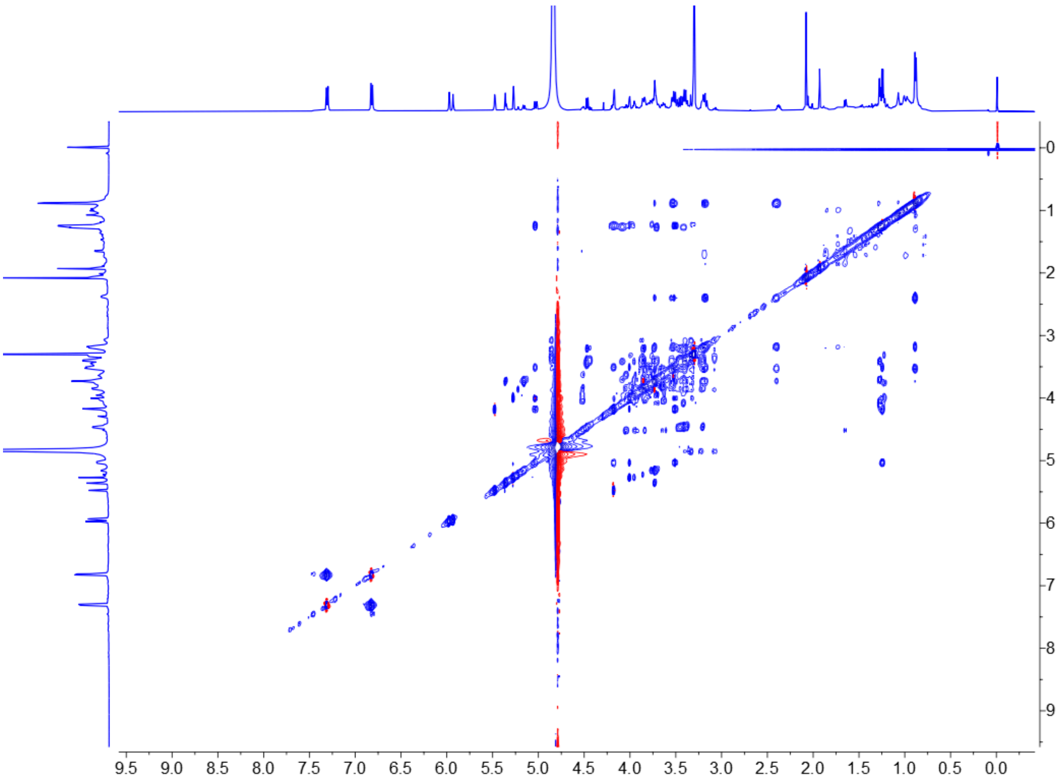

Figure S49. The TOCSY spectrum of compound **5** (600 MHz, CD_3OD).

6. Spectroscopic data for compound **6**

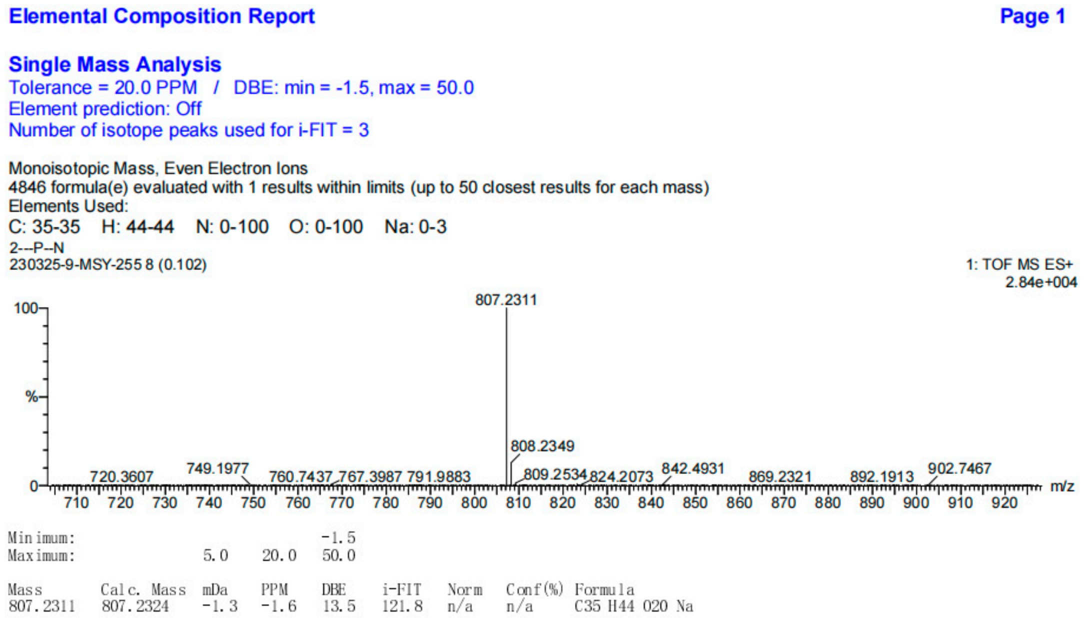

Figure S50. The HR-ESI-MS of compound **6**.

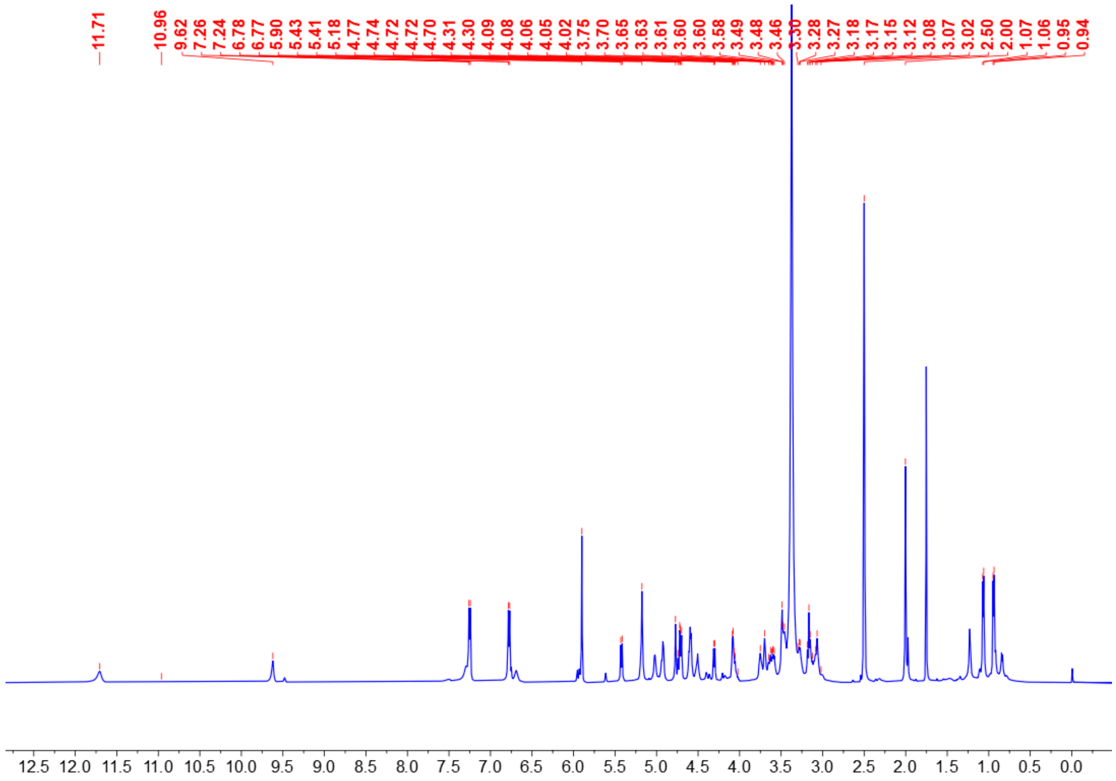

Figure S51. The ^{1}H NMR spectrum of compound **6** (600 MHz, DMSO-d_6).

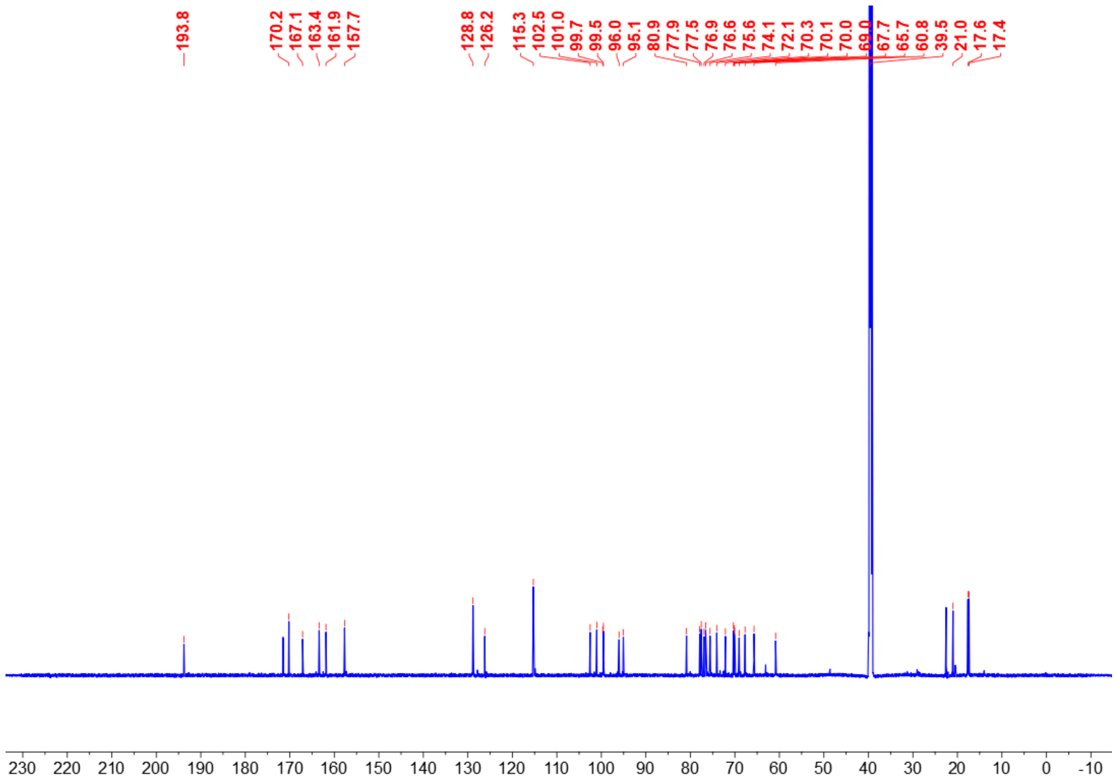

Figure S52. The ^{13}C NMR spectrum of compound **6** (151 MHz, DMSO-d_6).

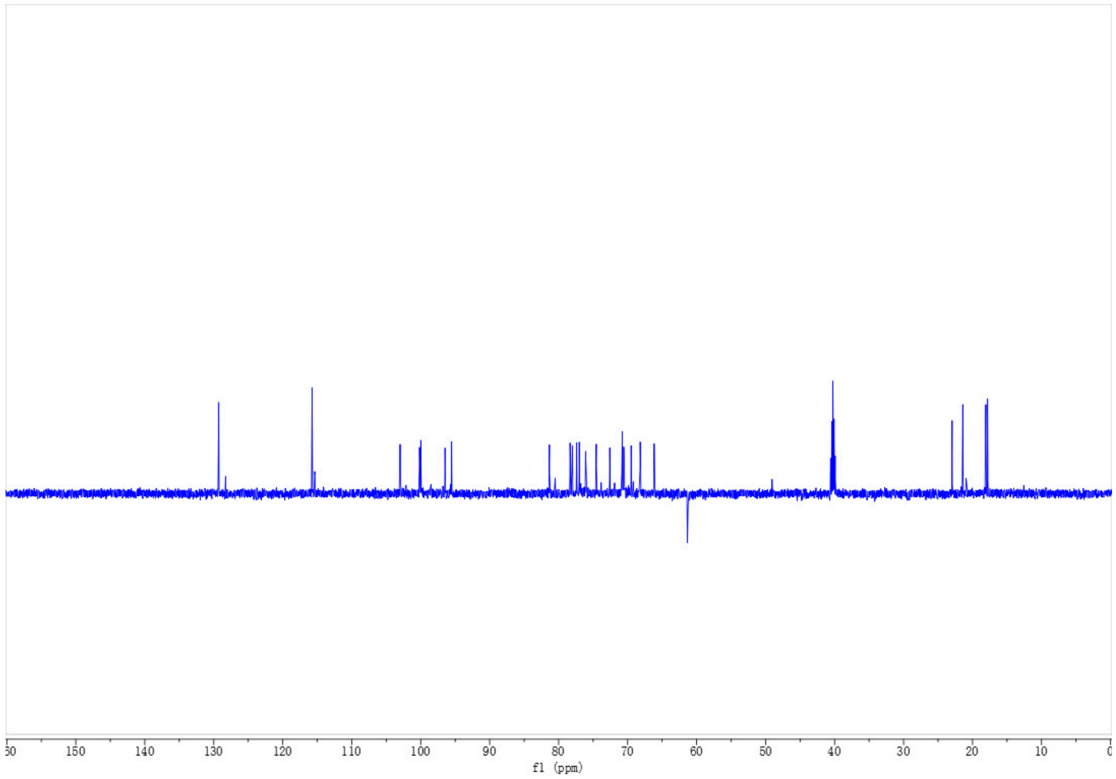

Figure S53. The DEPT 135 spectrum of compound **6** (151 MHz, DMSO-d_6).

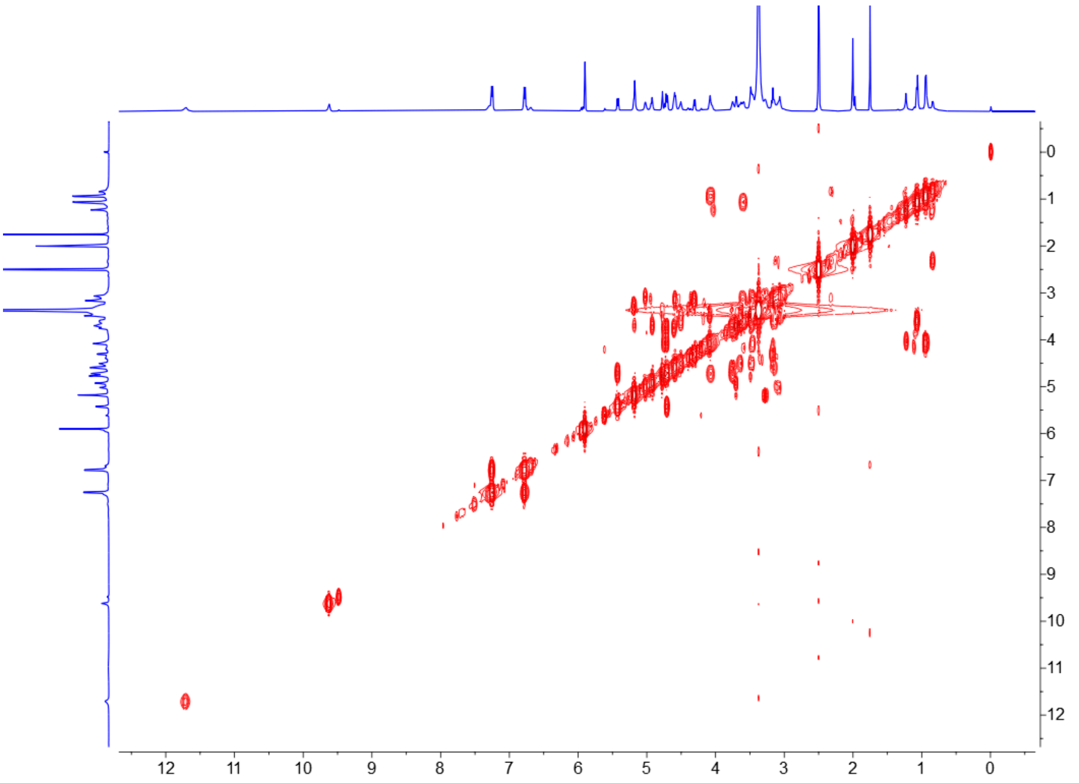

Figure S54. The ^{1}H-^{1}H COSY spectrum of compound **6** (600 MHz, DMSO-d_6).

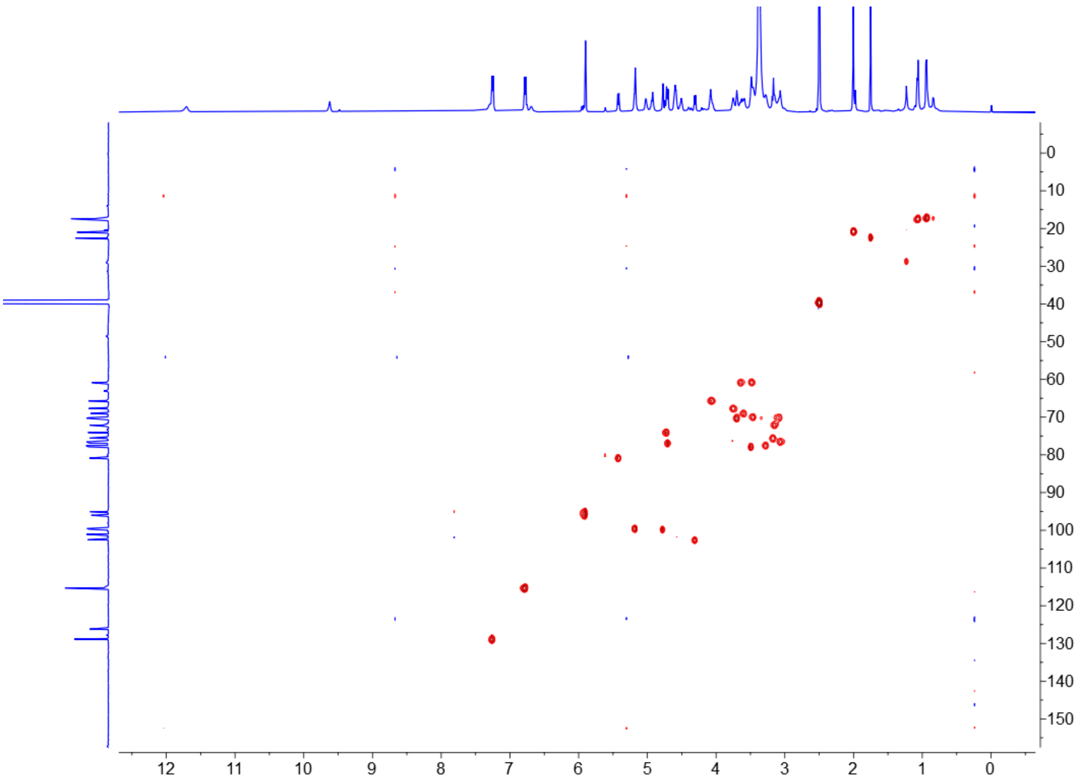

Figure S55. The HSQC spectrum of compound **6** (600/151 MHz, DMSO-d_6).

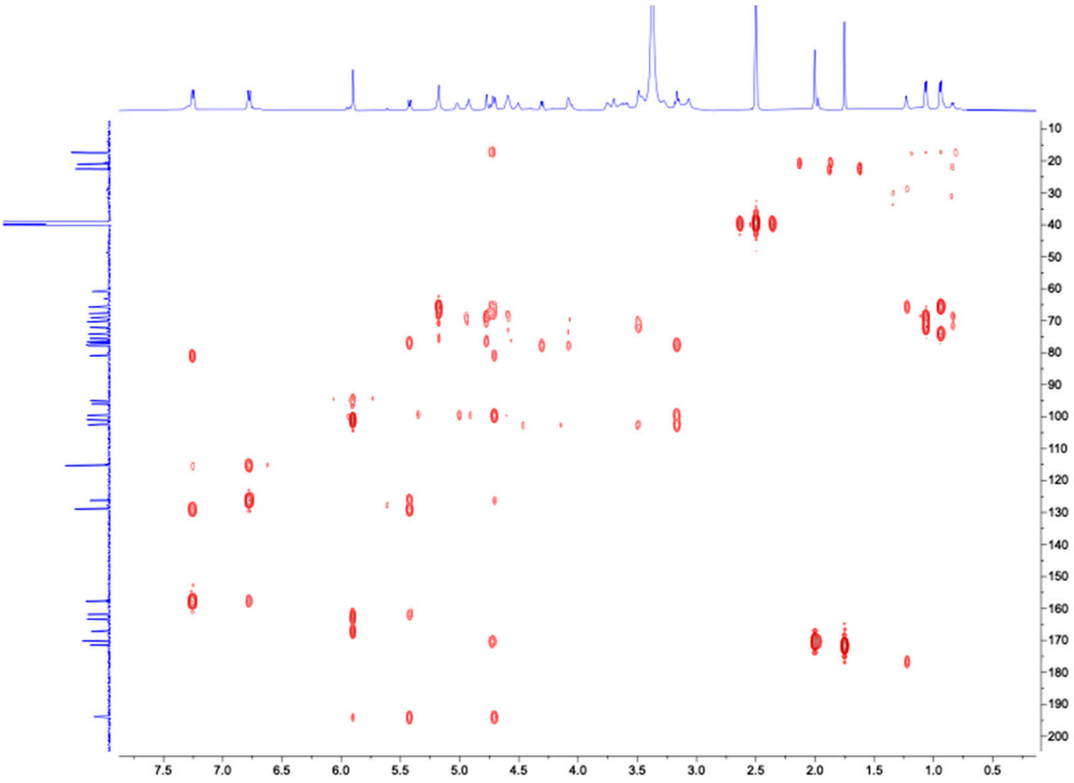

Figure S56. The HMBC spectrum of compound **6** (600/151 MHz, DMSO-*d*$_6$).

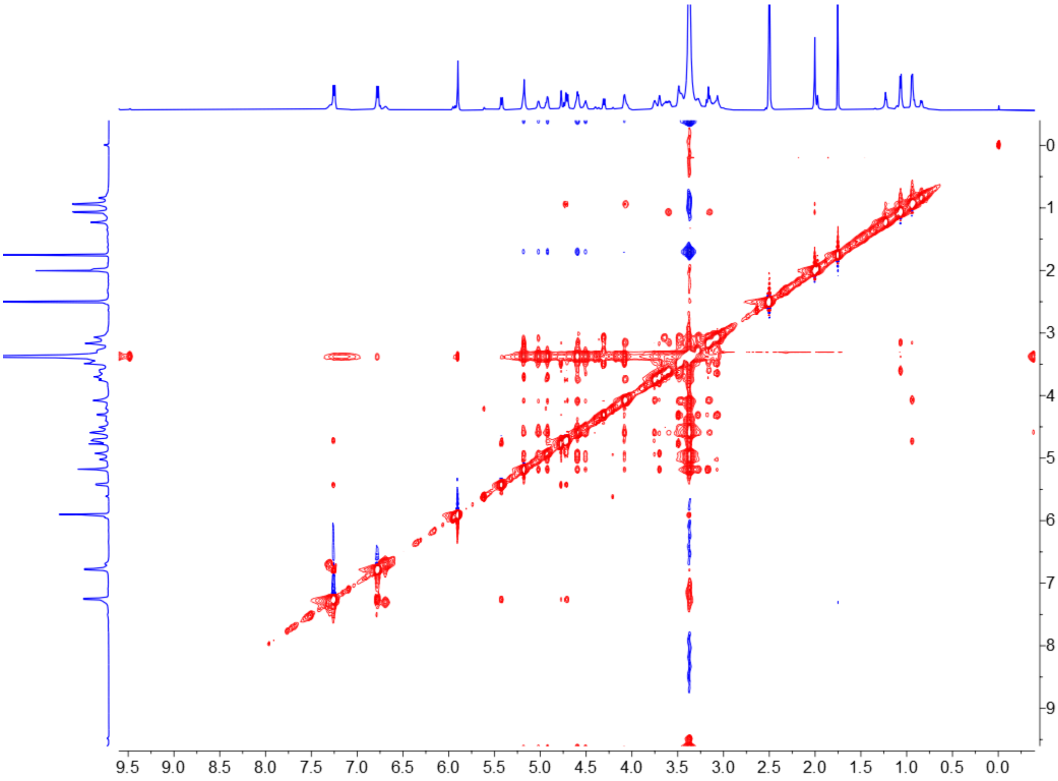

Figure S57. The NOESY spectrum of compound **6** (600 MHz, DMSO-*d*$_6$).

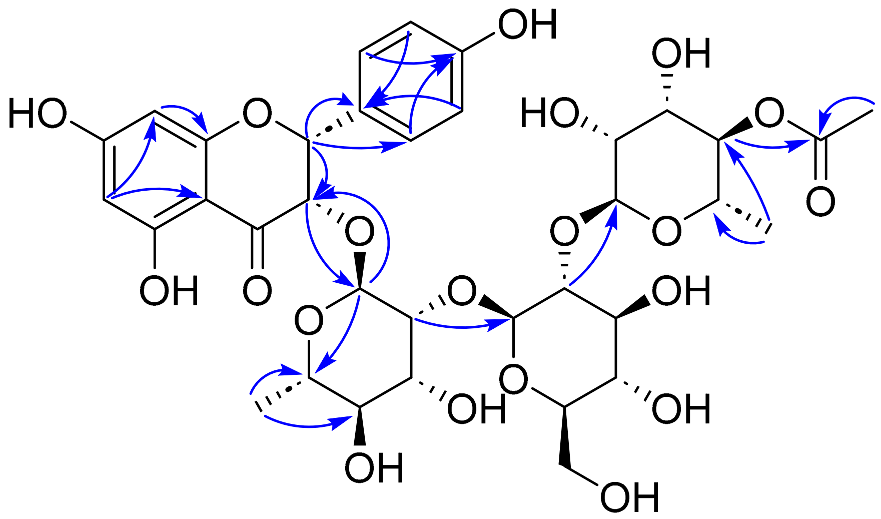

Figure S58. Key HMBC correlations of compound **6**.

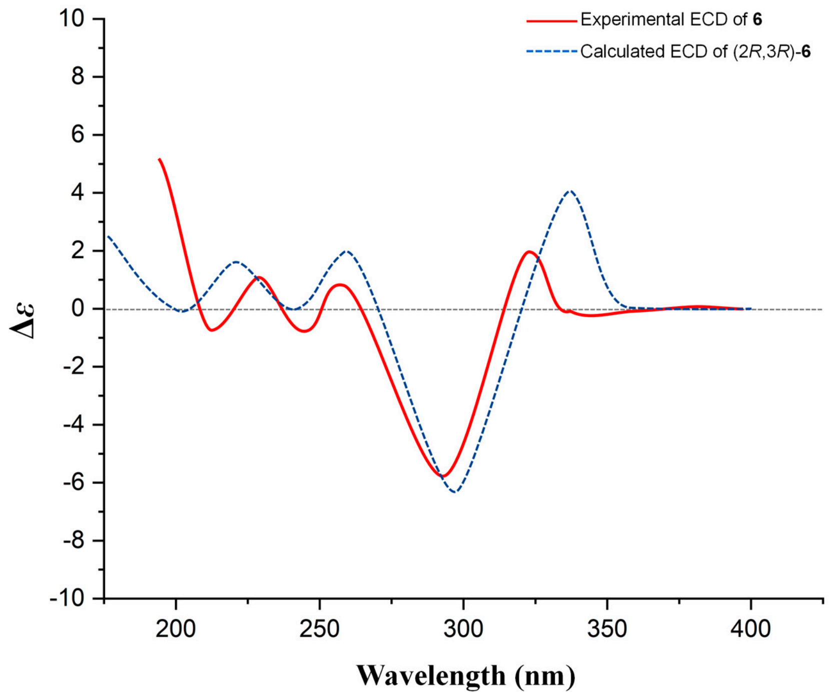

Figure S59. Experimental and calculated ECD spectra of compound **6**.

7. Spectroscopic data for compound **7**

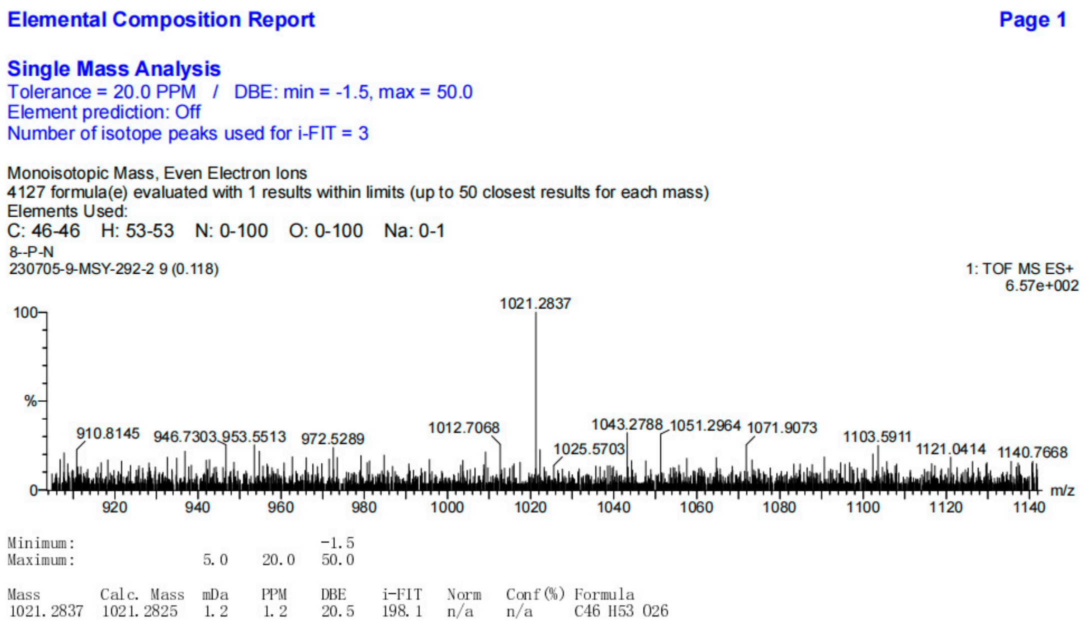

Figure S60. The HR-ESI-MS of compound **7**.

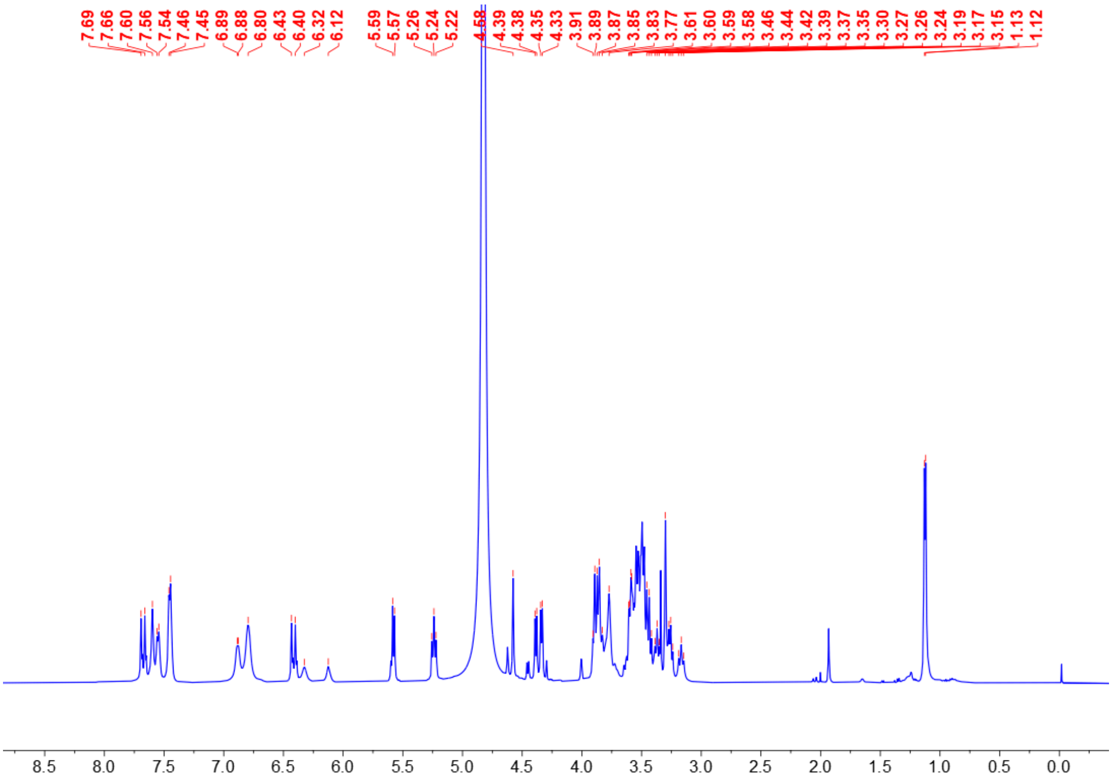

Figure S61. The ^{1}H NMR spectrum of compound **7** (600 MHz, CD_3OD).

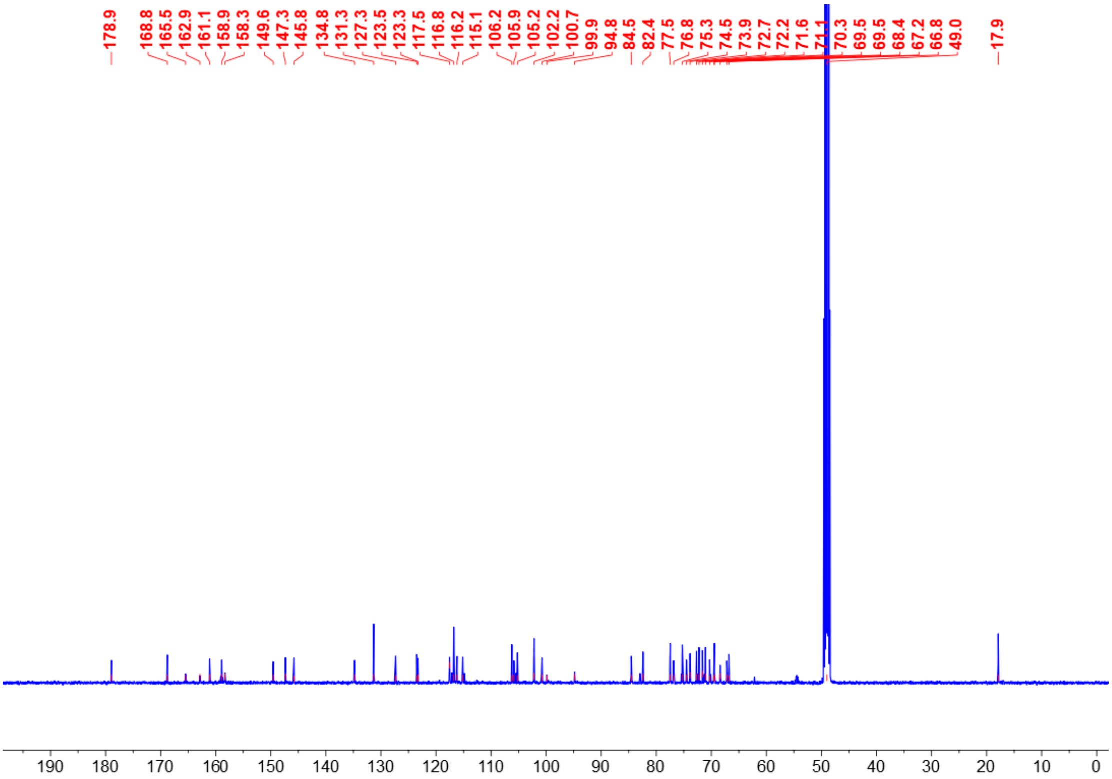

Figure S62. The ^{13}C NMR spectrum of compound **7** (151 MHz, CD_3OD).

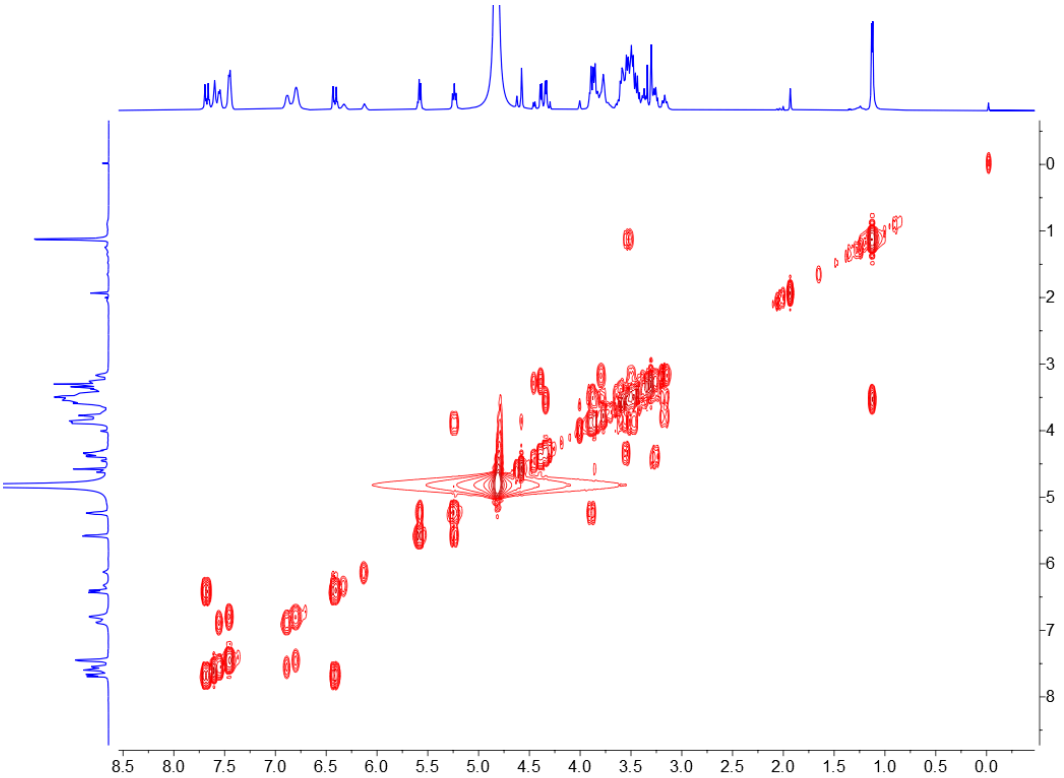

Figure S63. The ^{1}H-^{1}H COSY spectrum of compound **7** (600 MHz, CD_3OD).

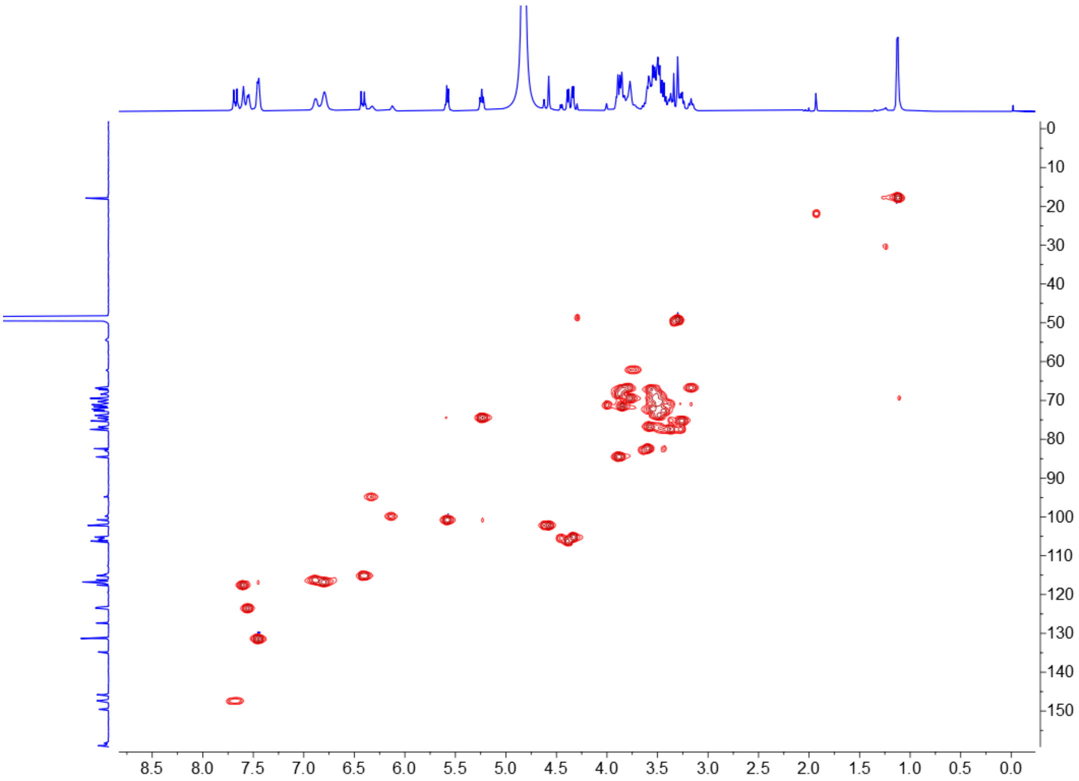

Figure S64. The HSQC spectrum of compound **7** (600/151 MHz, CD_3OD).

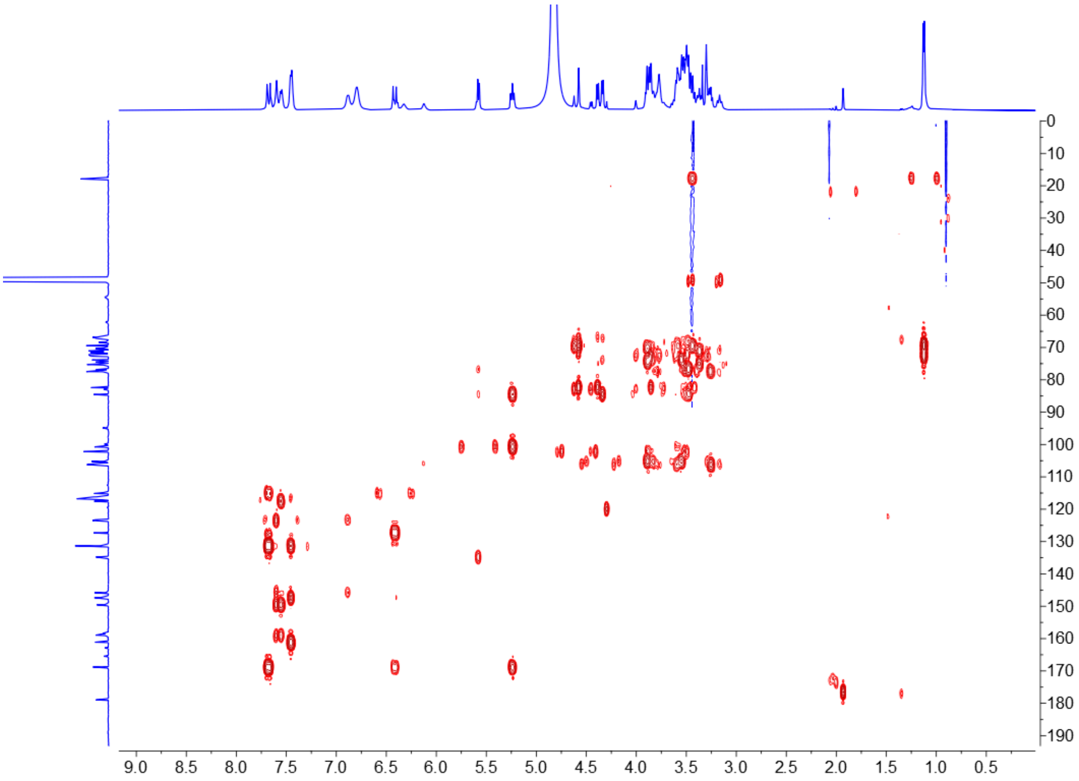

Figure S65. The HMBC spectrum of compound **7** (600/151 MHz, CD_3OD).

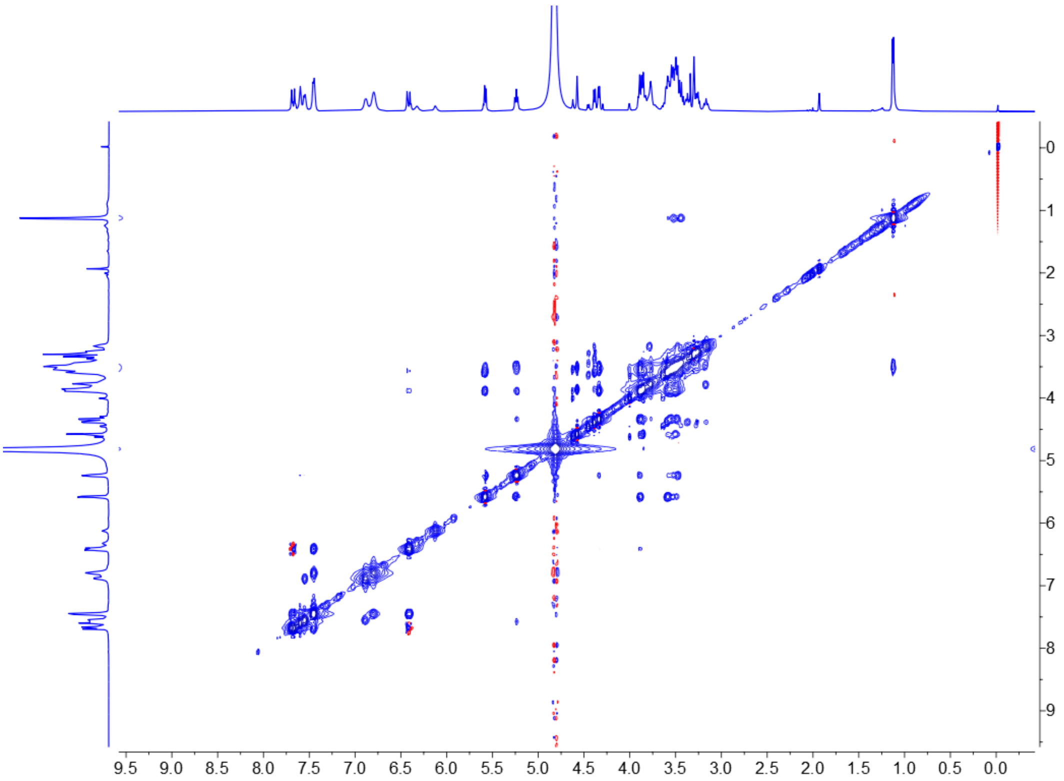

Figure S66. The NOESY spectrum of compound **7** (600 MHz, CD_3OD).

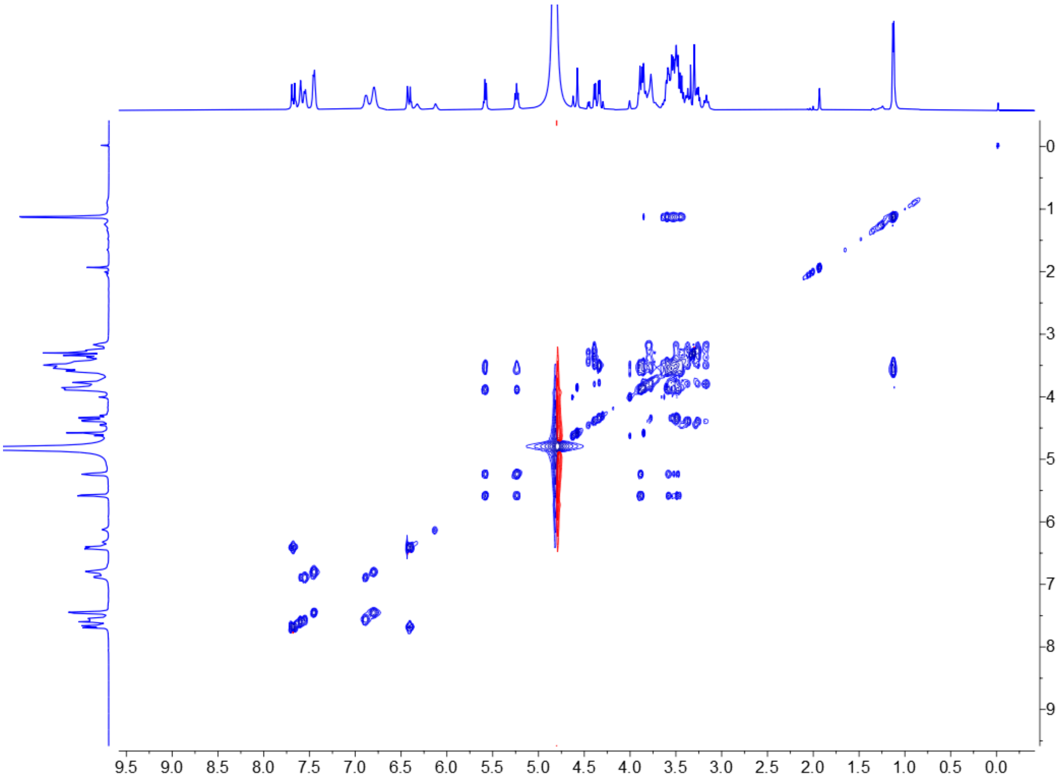

Figure S67. TheTOCSY spectrum of compound **7** (600 MHz, CD_3OD).

8. Spectroscopic data for compound **8**

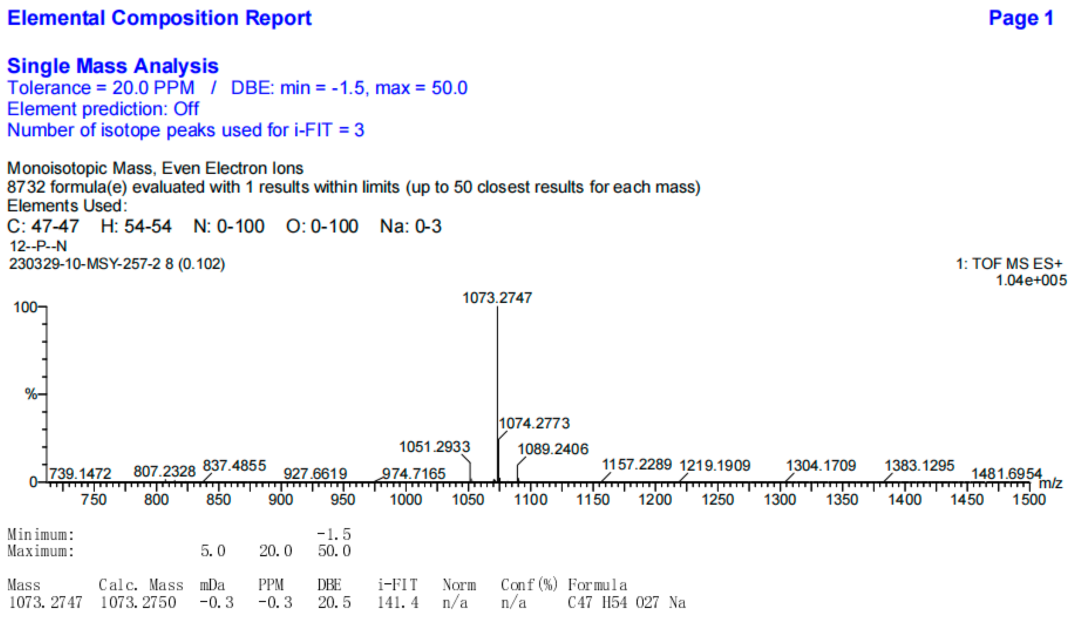

Figure S68. The HR-ESI-MS of compound **8**.

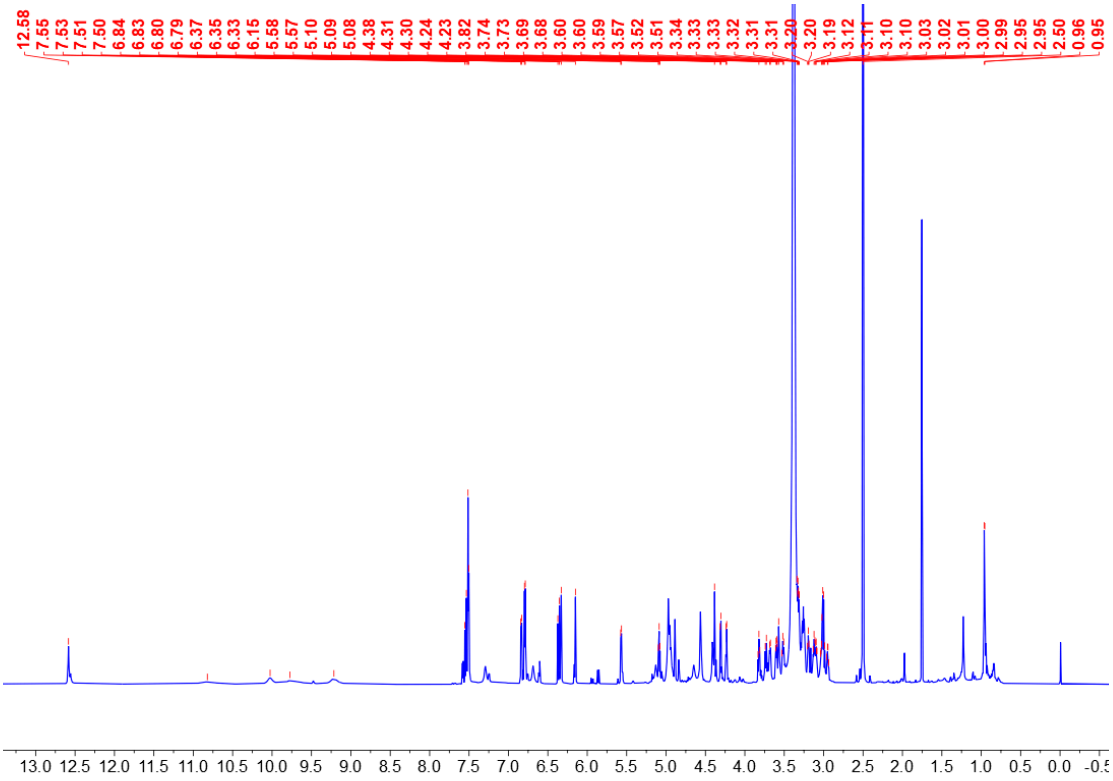

Figure S69. The ^{1}H NMR spectrum of compound **8** (600 MHz, DMSO-d_6).

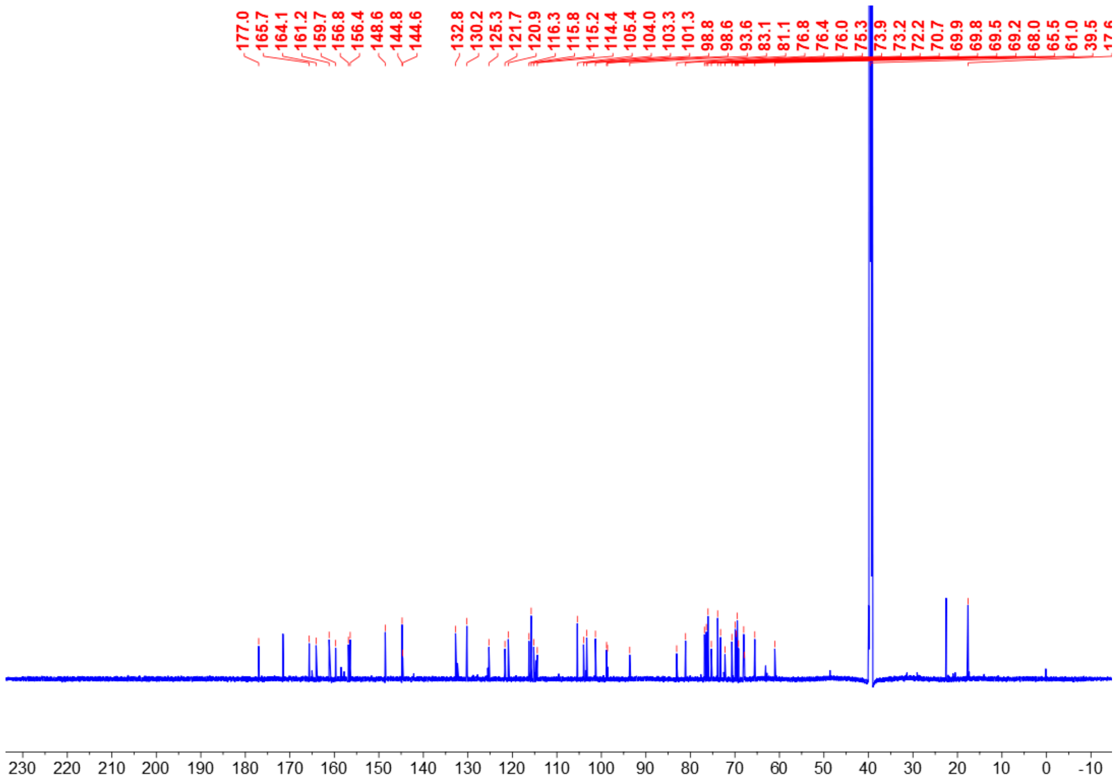

Figure S70. The ^{13}C NMR spectrum of compound **8** (151 MHz, DMSO-d_6).

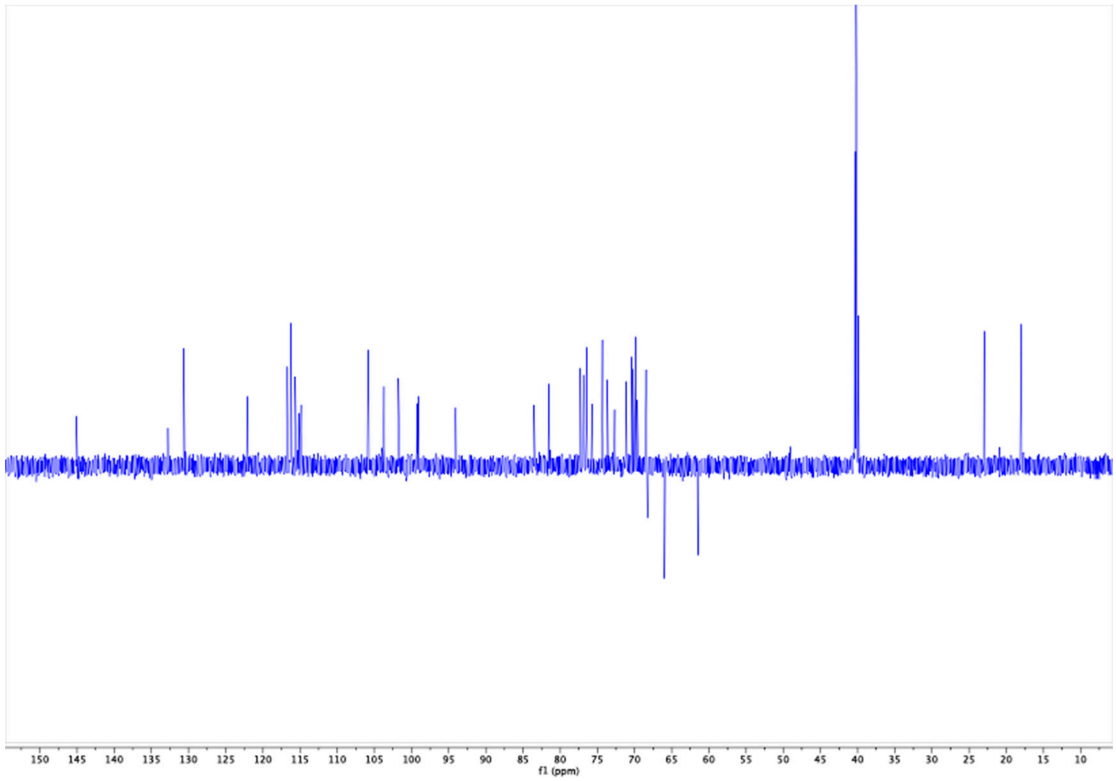

Figure S71. The DEPT135 spectrum of compound **8** (151 MHz, DMSO-d_6).

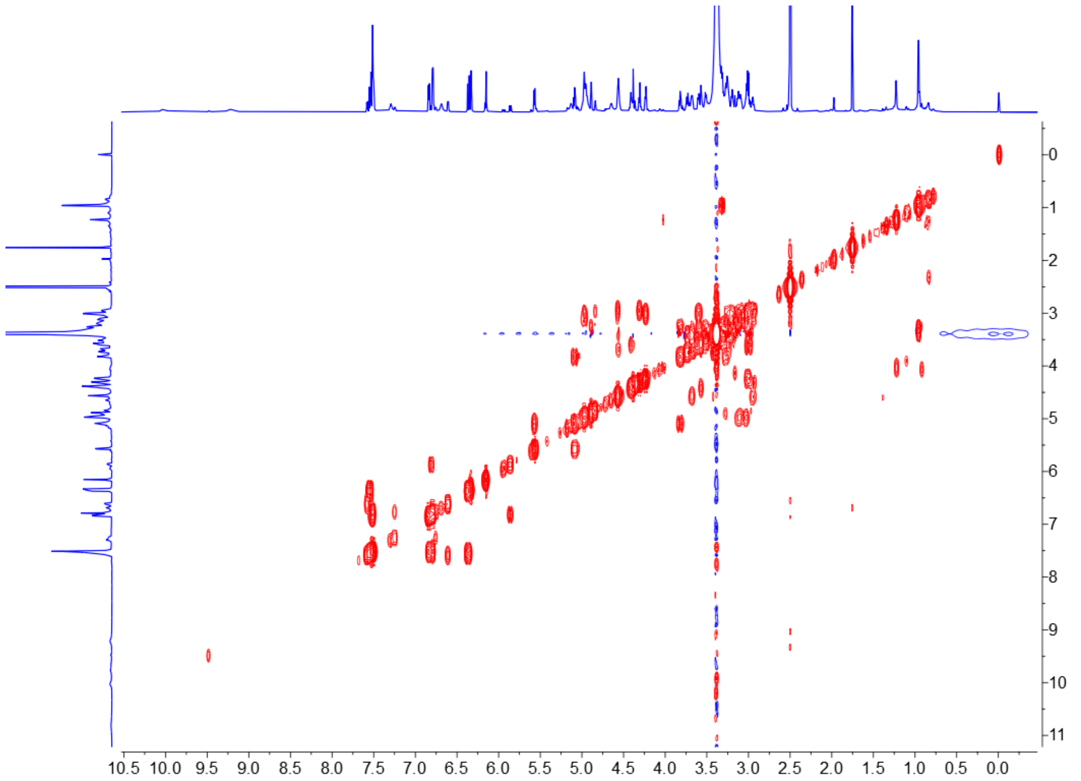

Figure S72. The ^{1}H-^{1}H COSY spectrum of compound **8** (600 MHz, DMSO-d_6).

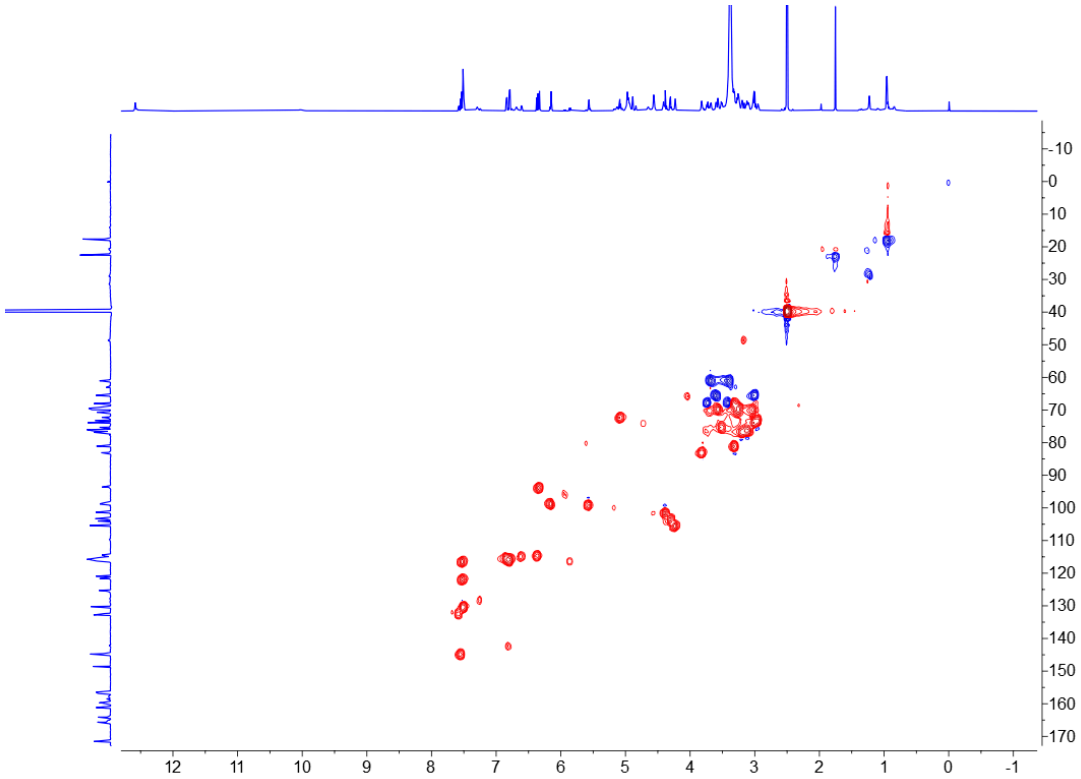

Figure S73. The HSQC spectrum of compound **8** (600/151 MHz, DMSO-d_6).

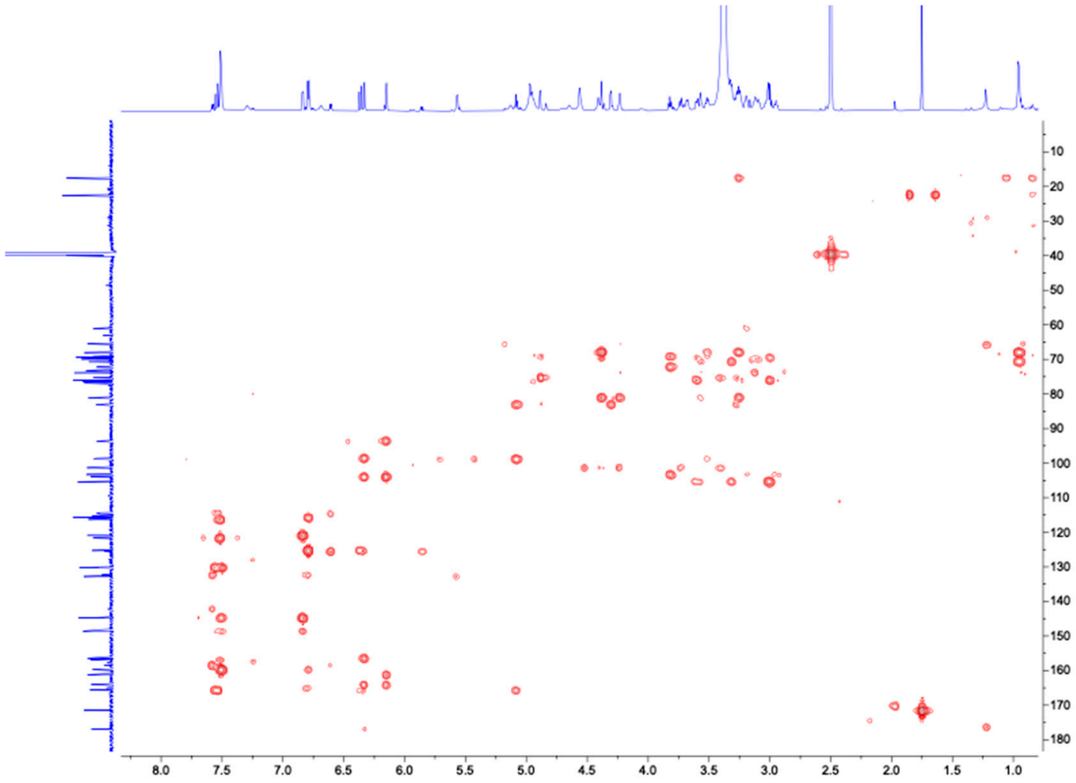

Figure S74. The HMBC spectrum of compound **8** (600/151 MHz, DMSO-d_6).

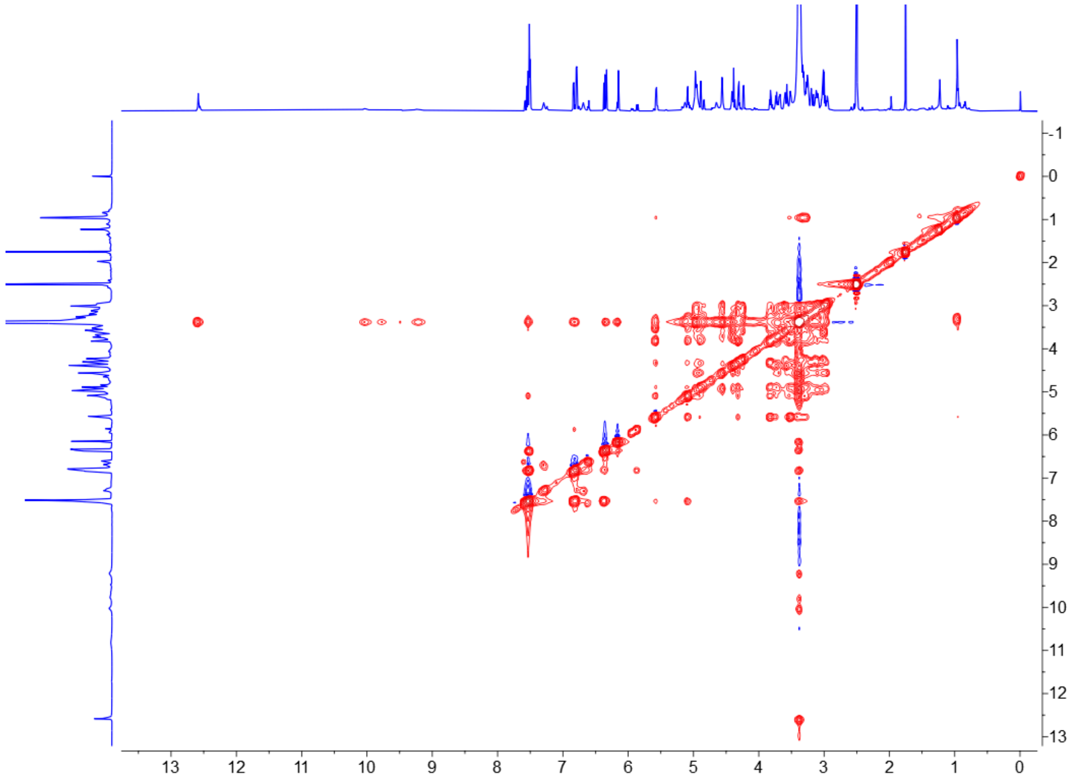

Figure S75. The NOESY spectrum of compound **8** (600 MHz, DMSO-d_6).

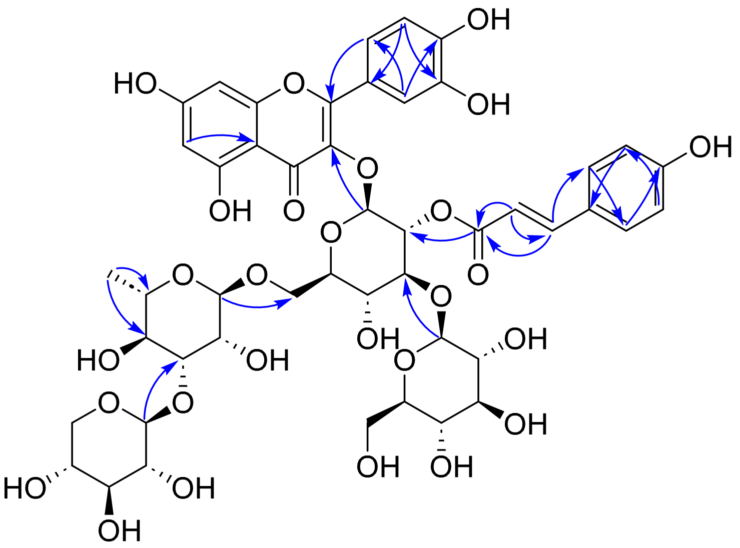

Figure S76. Key HMBC correlations of compound **8**.

9. Spectroscopic data for compound **9**

Elemental Composition Report **Page 1**

Single Mass Analysis
Tolerance = 20.0 PPM / DBE: min = -1.5, max = 50.0
Element prediction: Off
Number of isotope peaks used for i-FIT = 3

Monoisotopic Mass, Even Electron Ions
4242 formula(e) evaluated with 1 results within limits (up to 50 closest results for each mass)
Elements Used:
C: 47-47 H: 55-55 N: 0-100 O: 0-100 Na: 0-1
8--P-N
230705-9-MSY-293-2 4 (0.059)

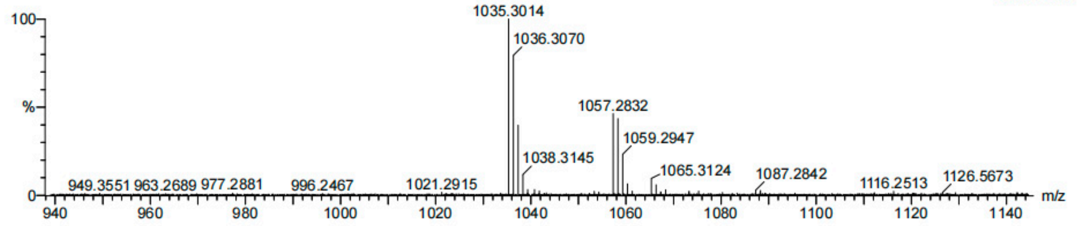

Minimum:				-1.5				
Maximum:		5.0	20.0	50.0				
Mass	Calc. Mass	mDa	PPM	DBE	i-FIT	Norm	Conf(%)	Formula
1035.3014	1035.2982	3.2	3.1	20.5	237.2	n/a	n/a	C47 H55 O26

Figure S77. The HR-ESI-MS of compound **9**.

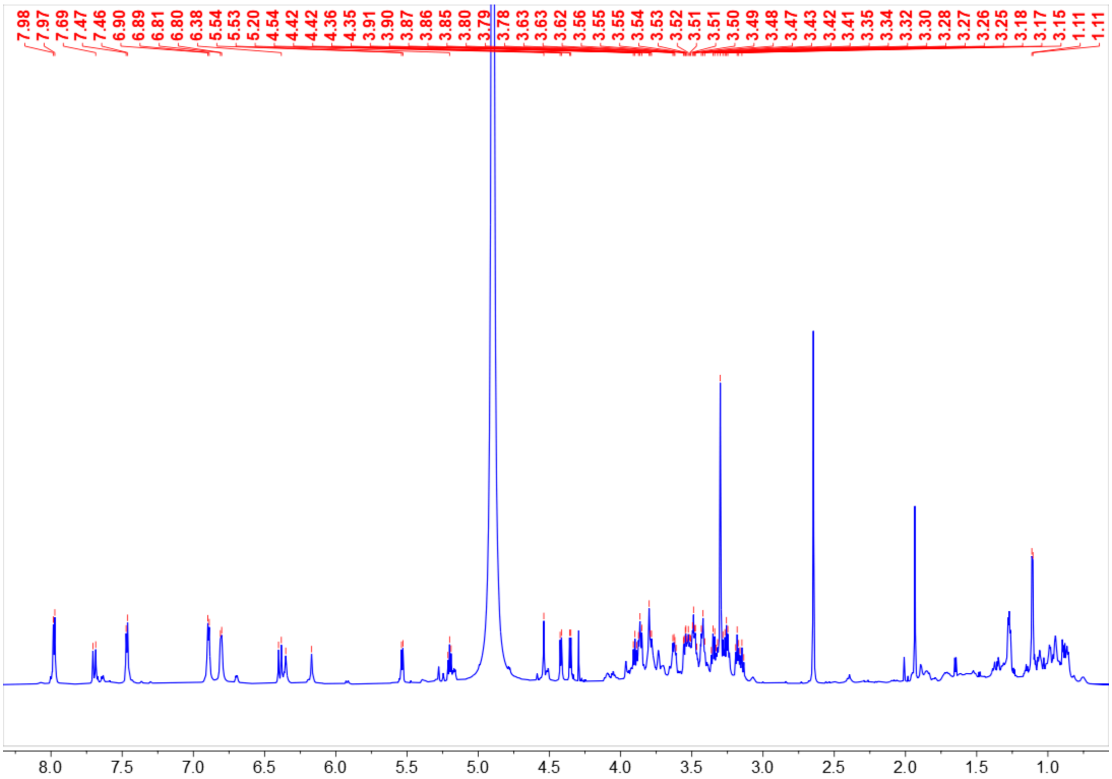

Figure S78. The ^{1}H NMR spectrum of compound **9** (600 MHz, CD_3OD).

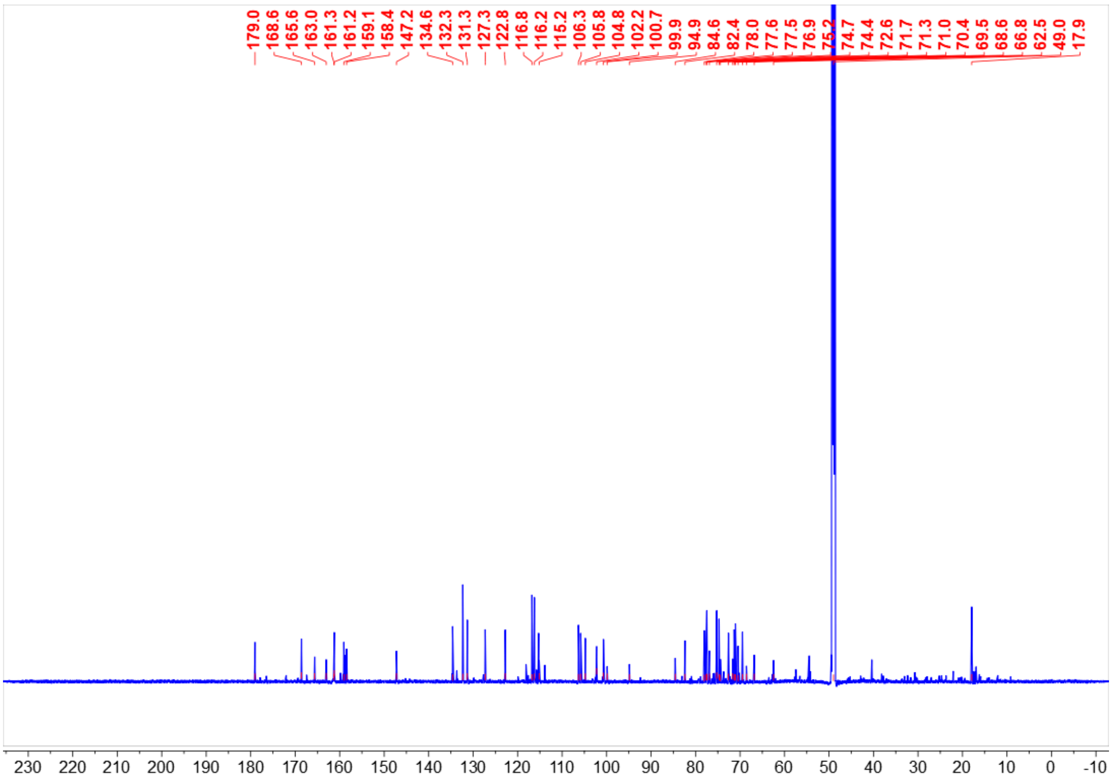

Figure S79. The ^{13}C NMR spectrum of compound **9** (151 MHz, CD_3OD).

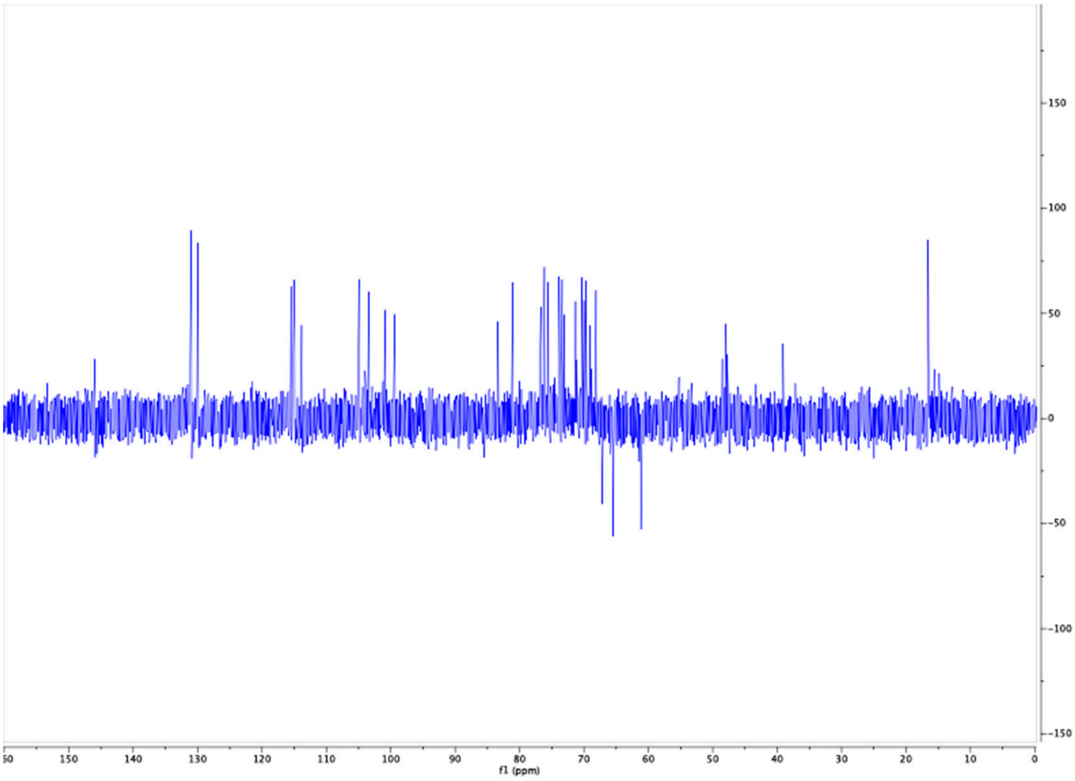

Figure S80. The DEPT135 spectrum of compound **9** (151 MHz, CD_3OD).

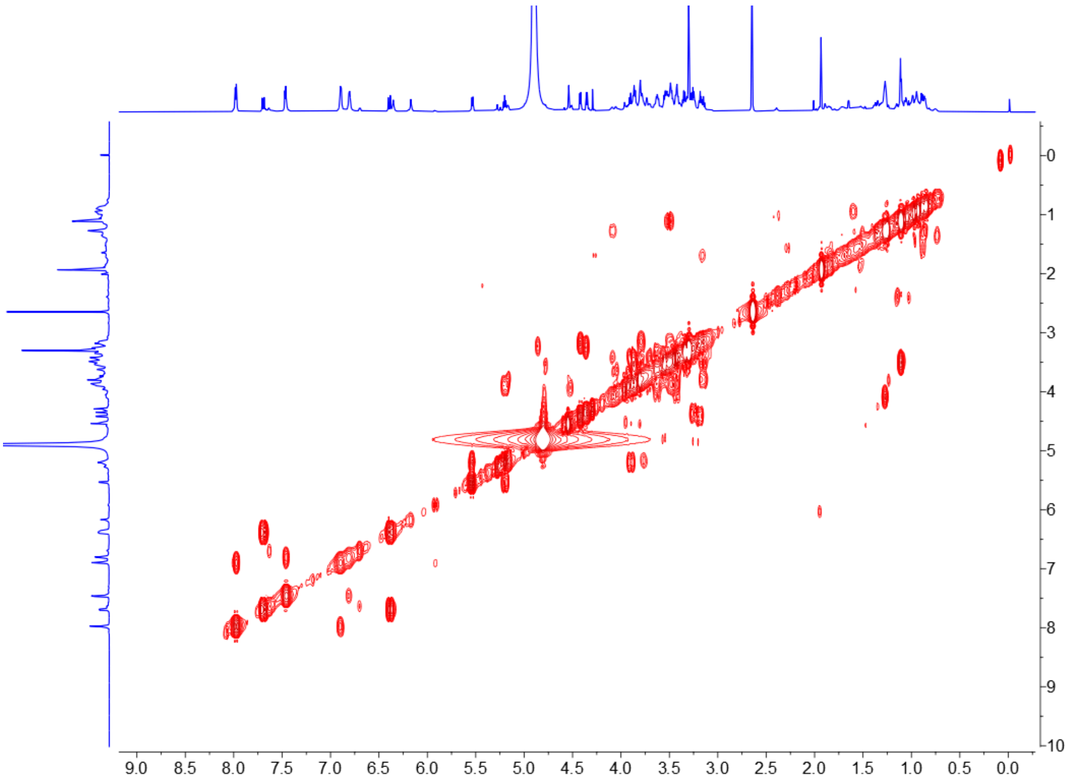

Figure S81. The ^{1}H-^{1}H COSY spectrum of compound **9** (600 MHz, CD_3OD).

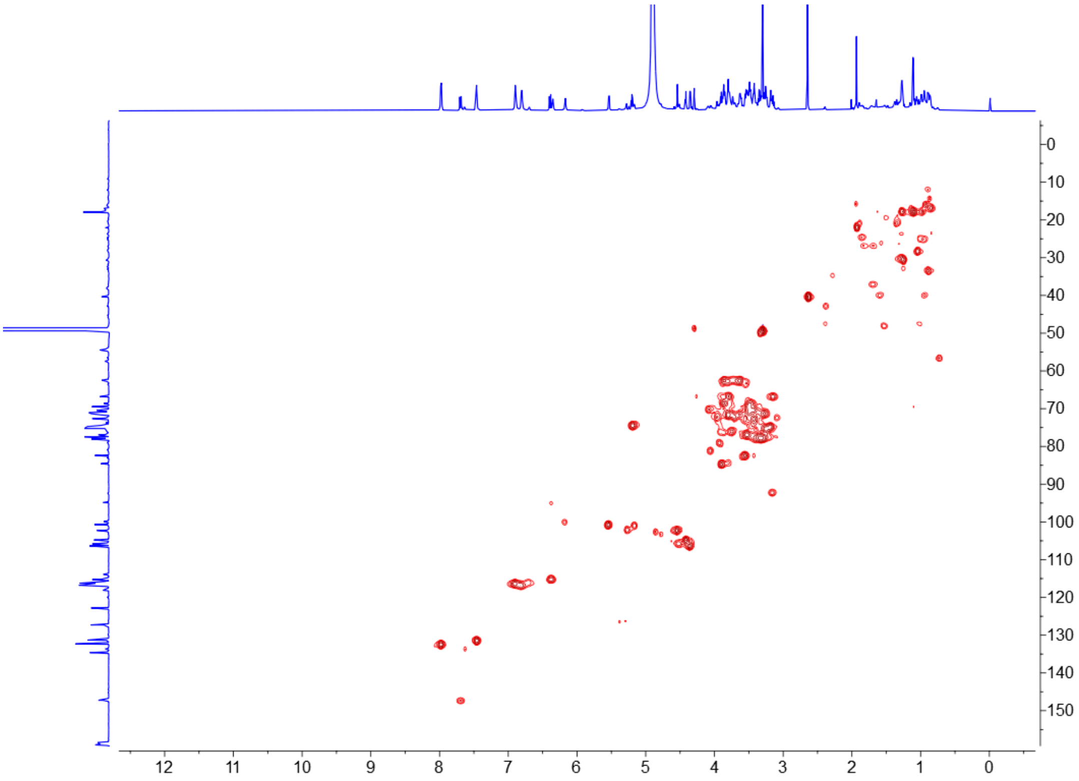

Figure S82. The HSQC spectrum of compound **9** (600/151 MHz, CD_3OD).

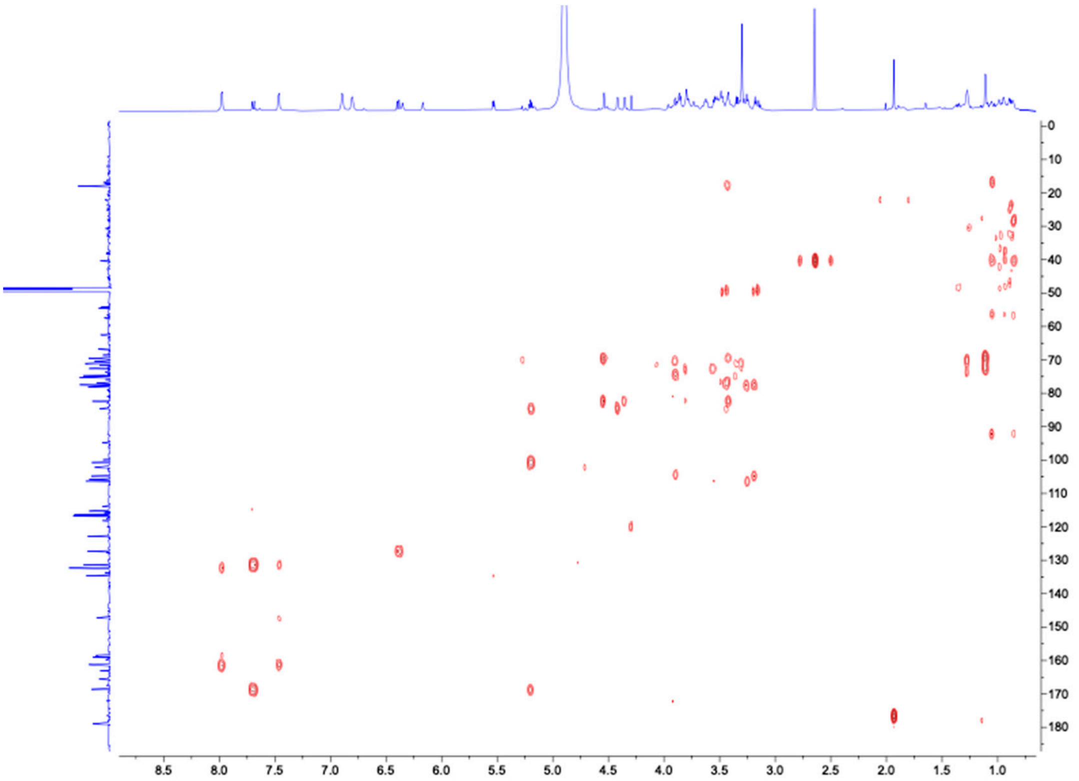

Figure S83. The HMBC spectrum of compound **9** (600/151 MHz, CD_3OD).

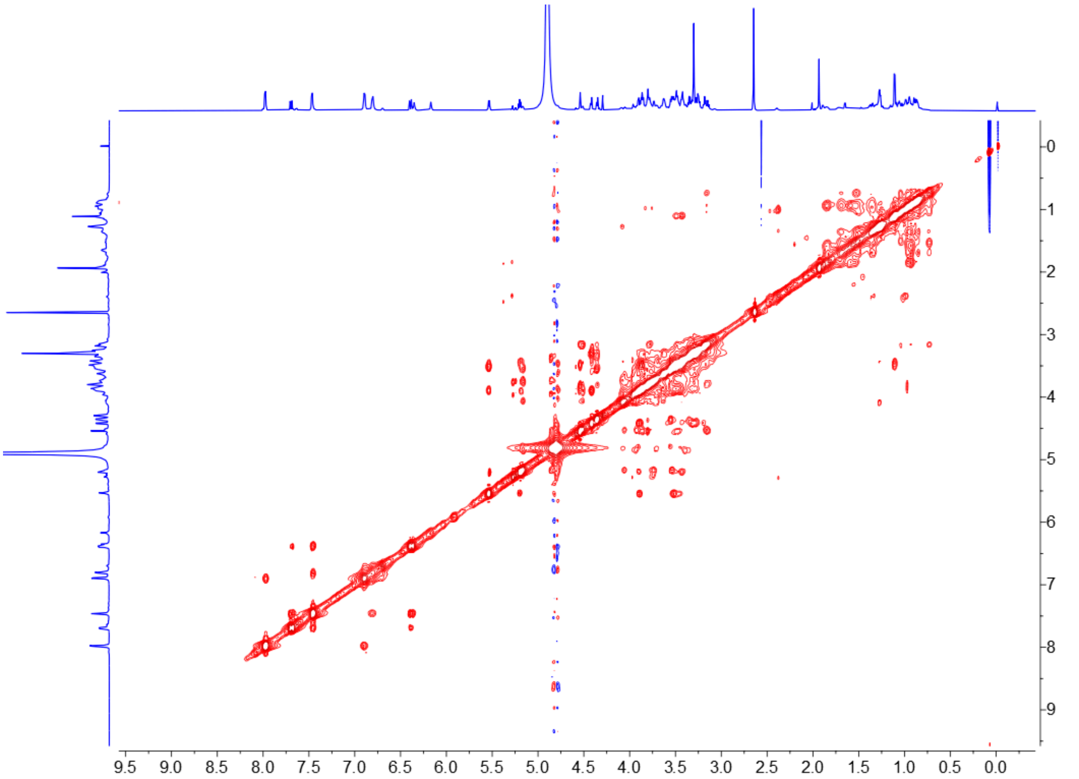

Figure S84. The NOESY spectrum of compound **9** (600 MHz, CD_3OD).

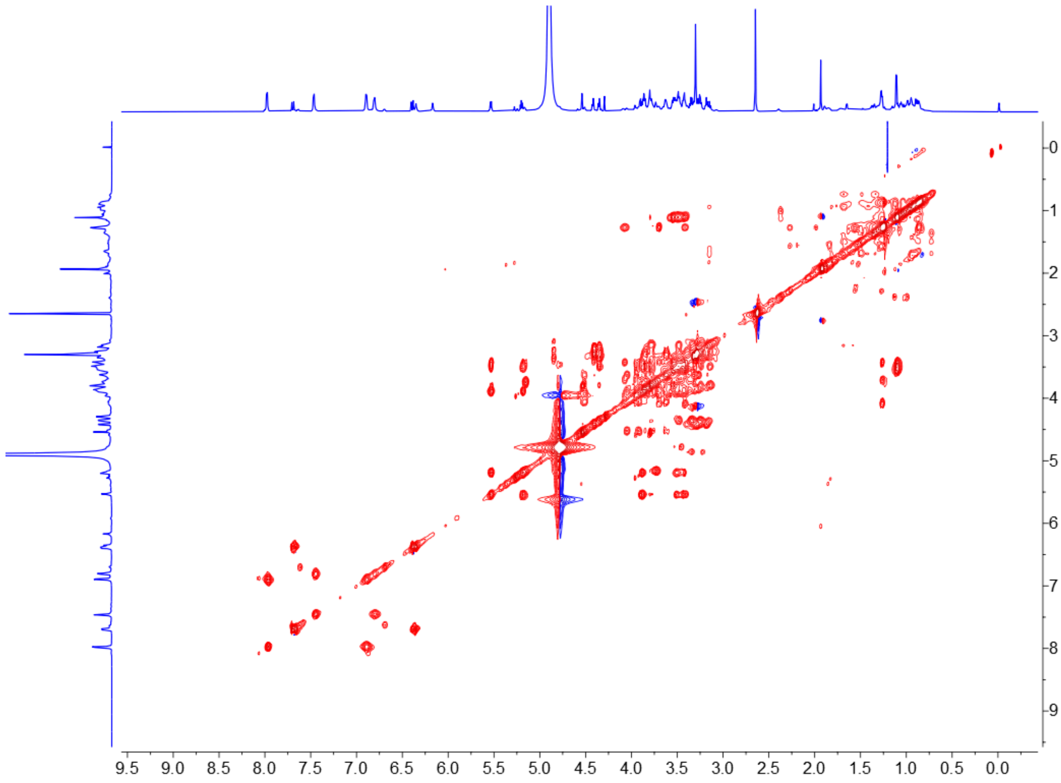

Figure S85. The TOCSY spectrum of compound **9** (600 MHz, CD_3OD).

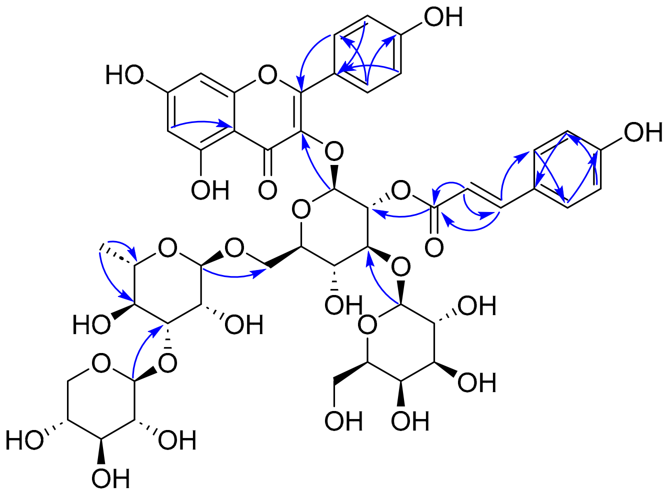

Figure S86. Key HMBC correlations of compound **9**.

10. Spectroscopic data for compound **10**

Elemental Composition Report **Page 1**

Single Mass Analysis
Tolerance = 20.0 PPM / DBE: min = -1.5, max = 50.0
Element prediction: Off
Number of isotope peaks used for i-FIT = 3

Monoisotopic Mass, Even Electron Ions
4005 formula(e) evaluated with 1 results within limits (up to 50 closest results for each mass)
Elements Used:
C: 46-46 H: 53-53 N: 0-100 O: 0-100 Na: 0-1
8--P-N
230705-9-MSY-294-2 4 (0.059)

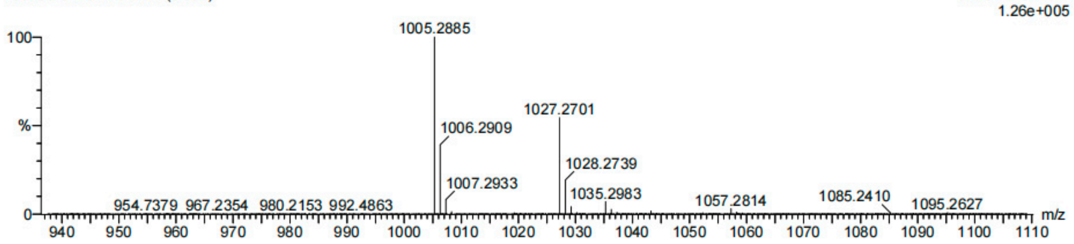

Minimum:				-1.5				
Maximum:		5.0	20.0	50.0				
Mass	Calc. Mass	mDa	PPM	DBE	i-FIT	Norm	Conf(%)	Formula
1005.2885	1005.2876	0.9	0.9	20.5	241.1	n/a	n/a	C46 H53 O25

Figure S87. The HR-ESI-MS of compound **10**.

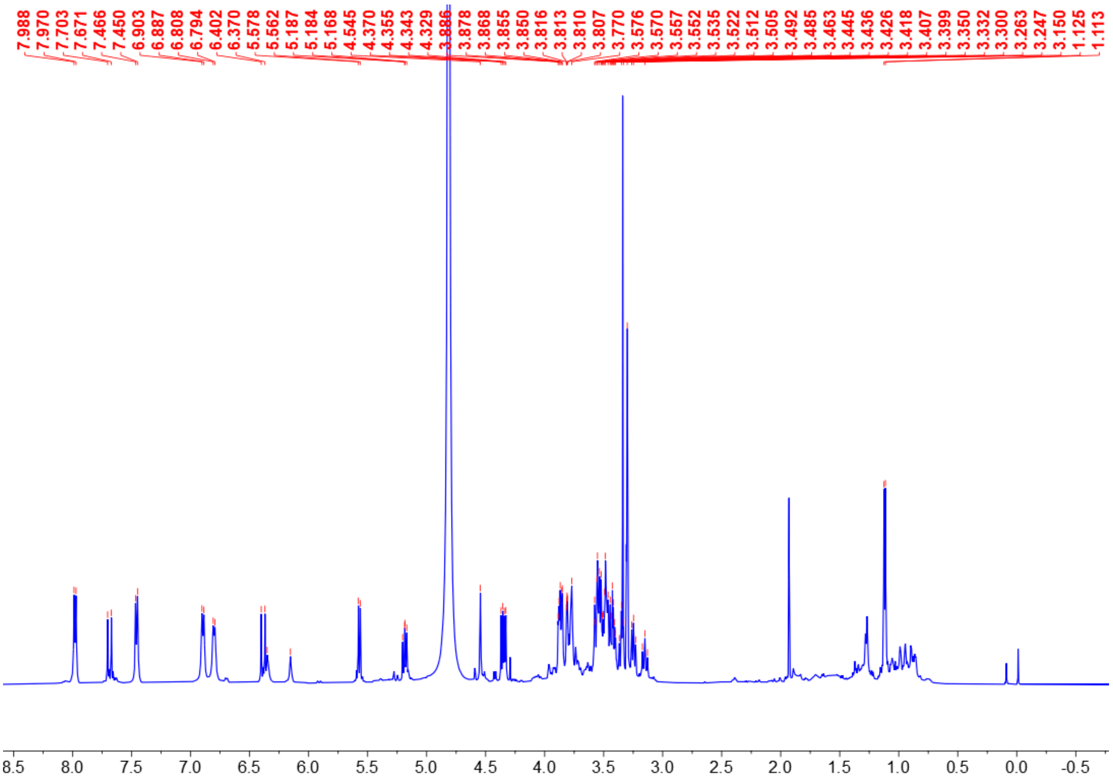

Figure S88. The ^{1}H NMR spectrum of compound **10** (600 MHz, CD_3OD).

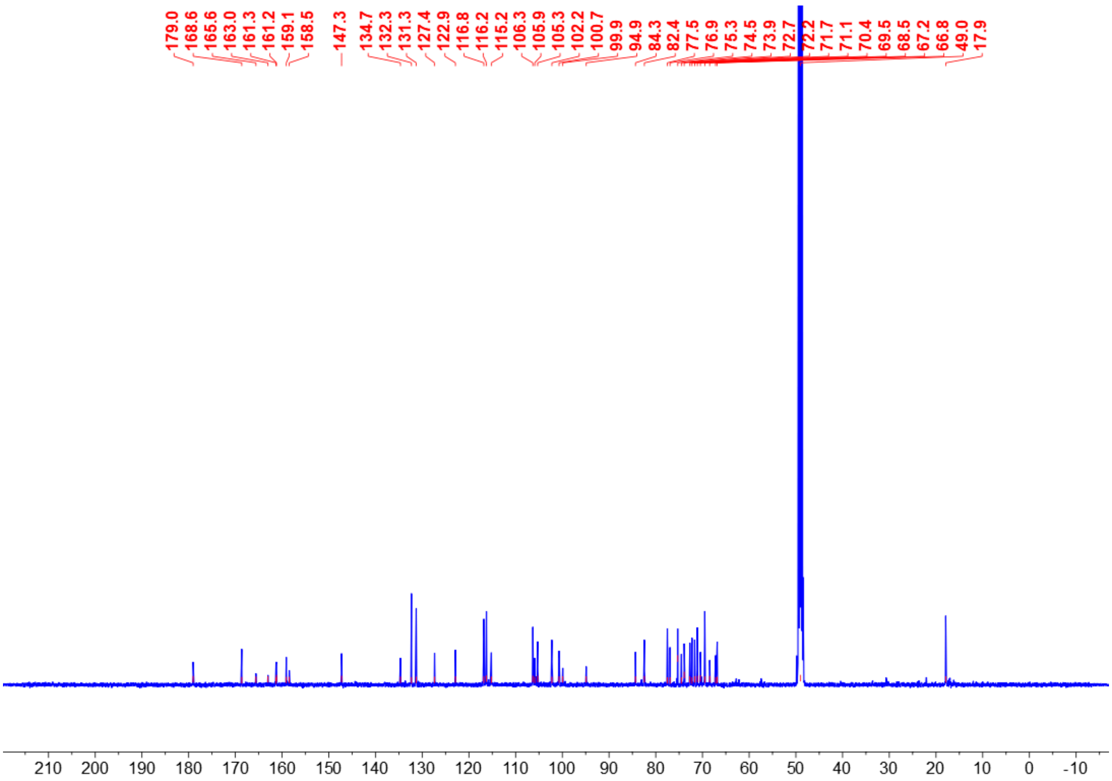

Figure S89. The ^{13}C NMR spectrum of compound **10** (151 MHz, CD_3OD).

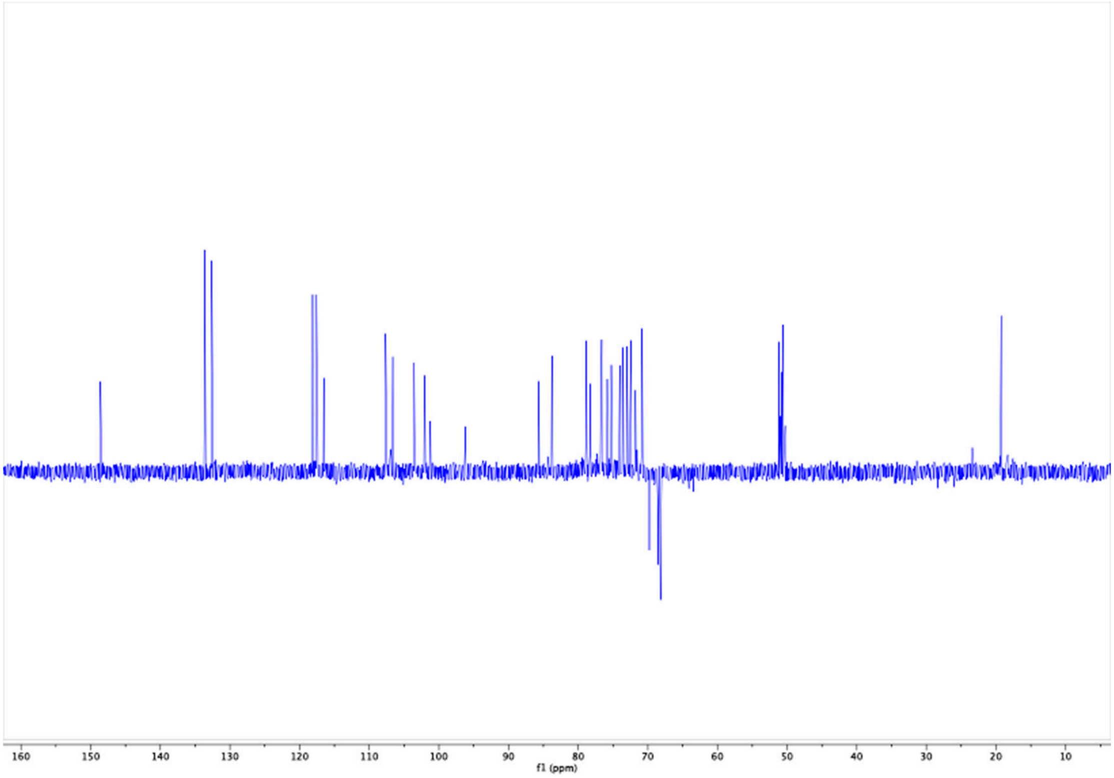

Figure S90. The DEPT135 spectrum of compound **10** (151 MHz, CD_3OD).

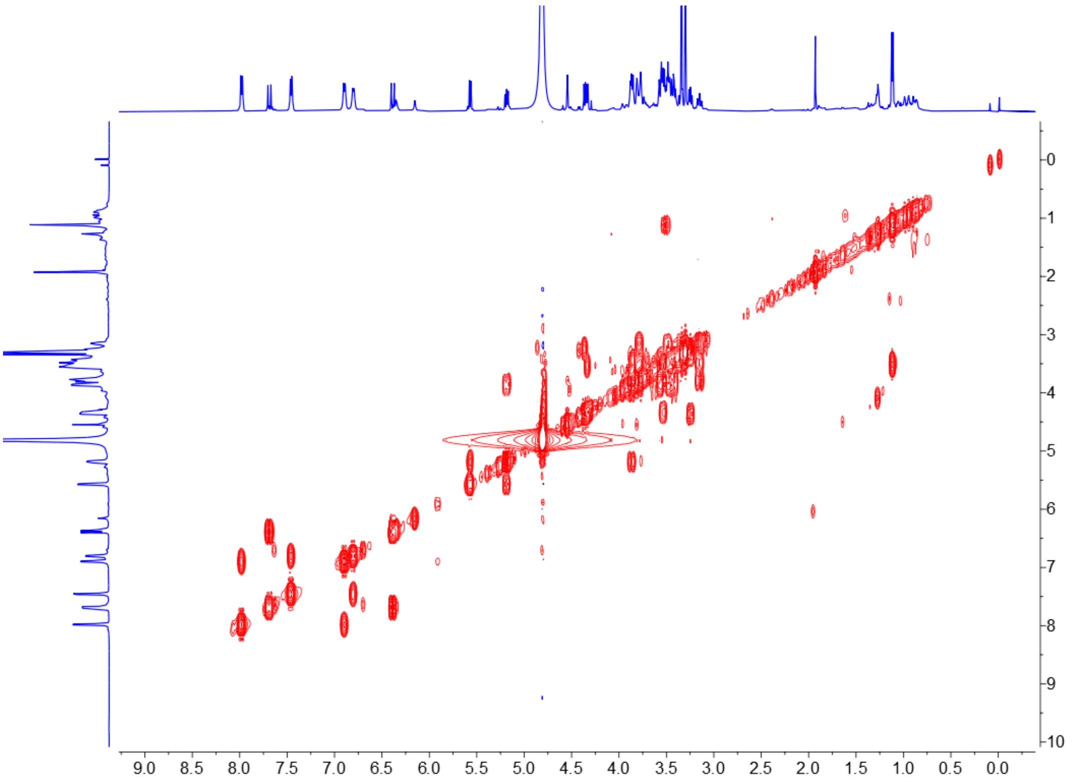

Figure S91. The ^{1}H-^{1}H COSY spectrum of compound **10** (600 MHz, CD_3OD).

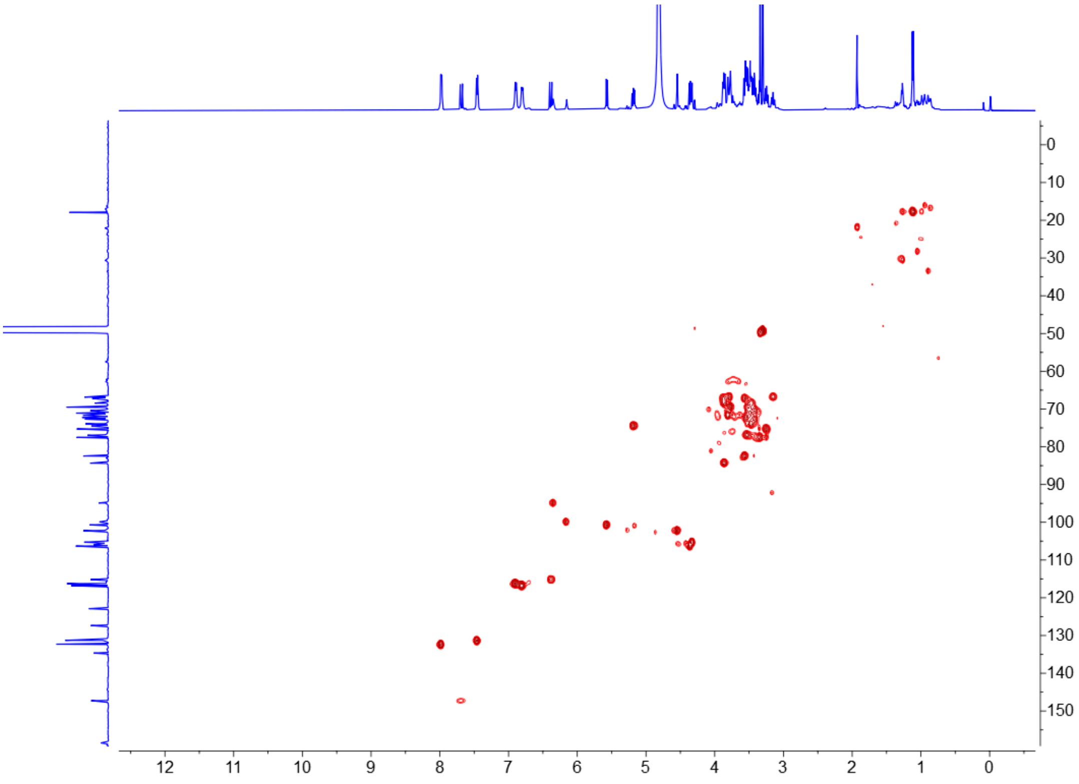

Figure S92. The HSQC spectrum of compound **10** (600/151 MHz, CD_3OD).

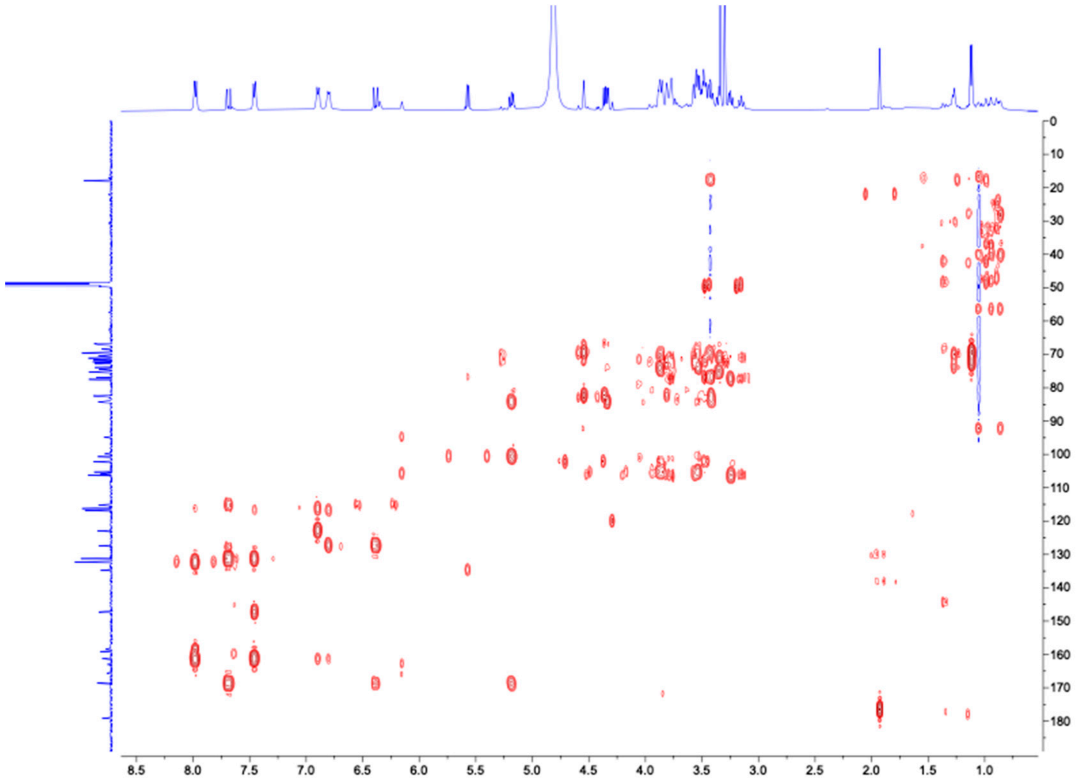

Figure S93. The HMBC spectrum of compound **10** (600/151 MHz, CD_3OD).

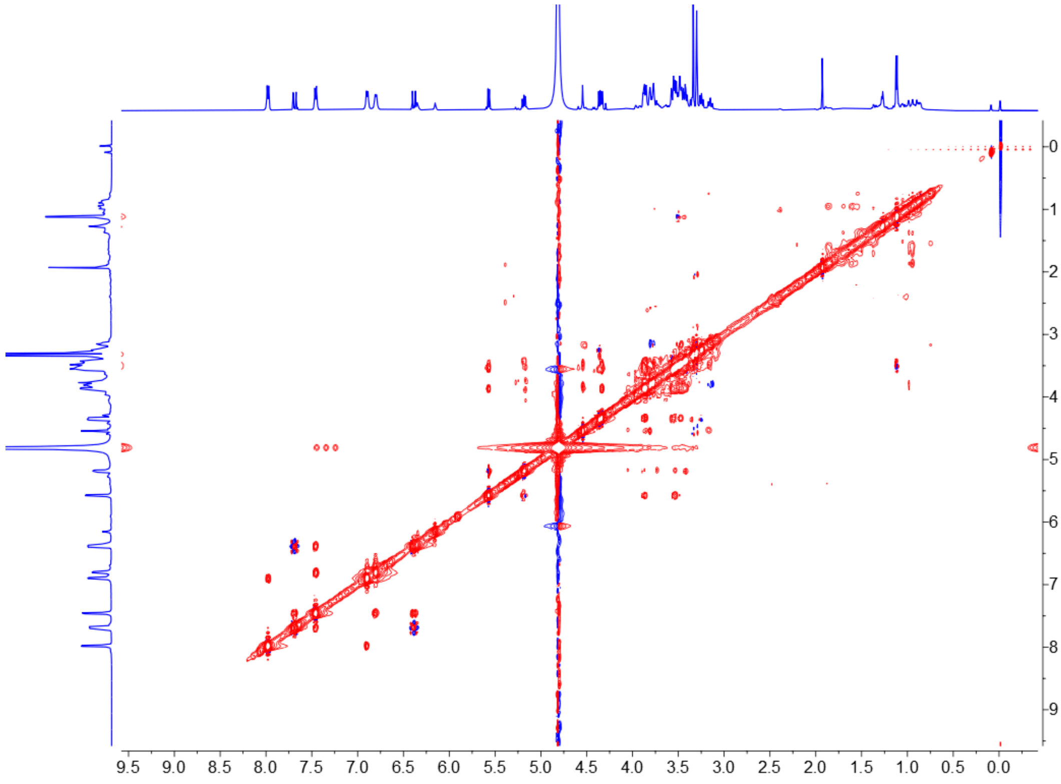

Figure S94. The NOESY spectrum of compound **10** (600 MHz, CD_3OD).

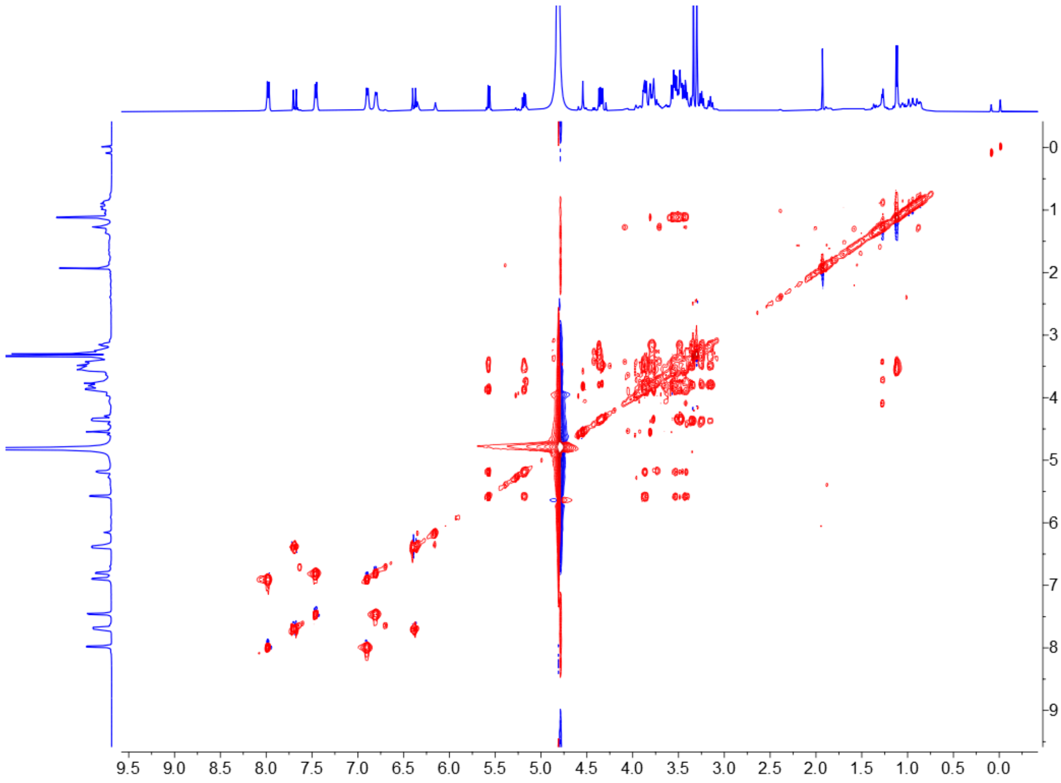

Figure S95. The TOCSY spectrum of compound **10** (600 MHz, CD_3OD).

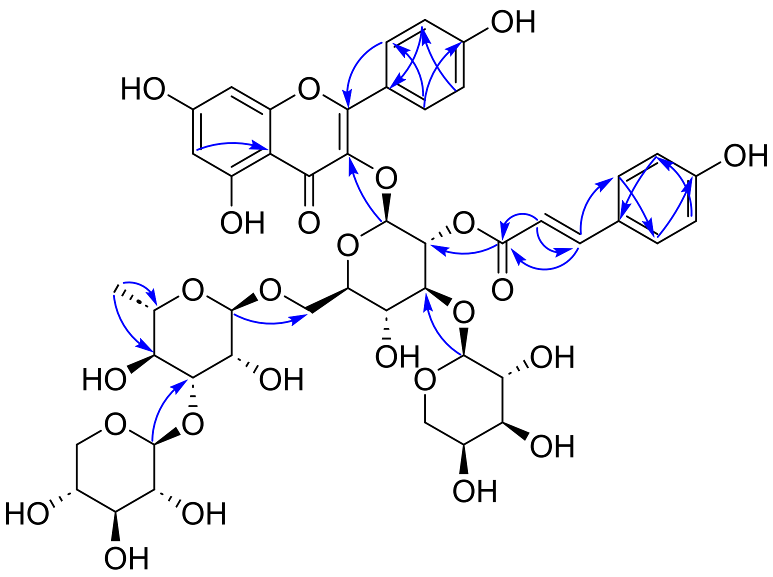

Figure S96. Key HMBC correlations of compound **10**.

11. ^{1}H and ^{13}C NMR spectroscopic data for compound **11−15**

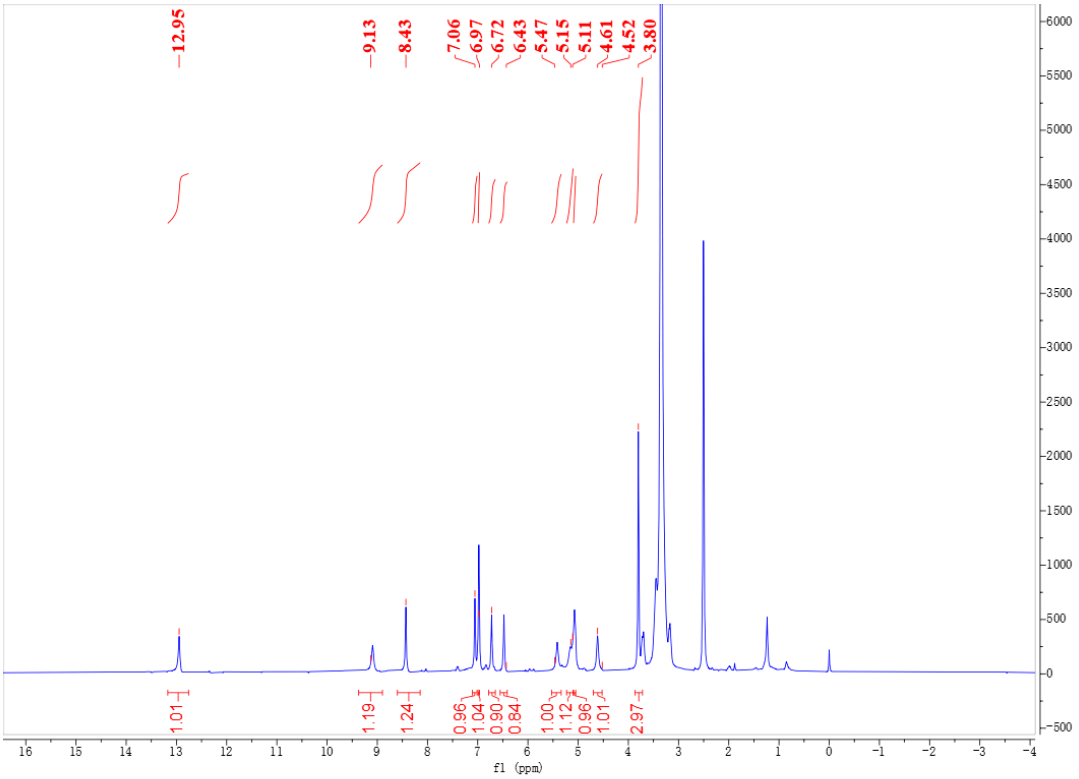

Figure S97. The ^{1}H NMR spectrum of compound **11** (600 MHz, DMSO-d_6).

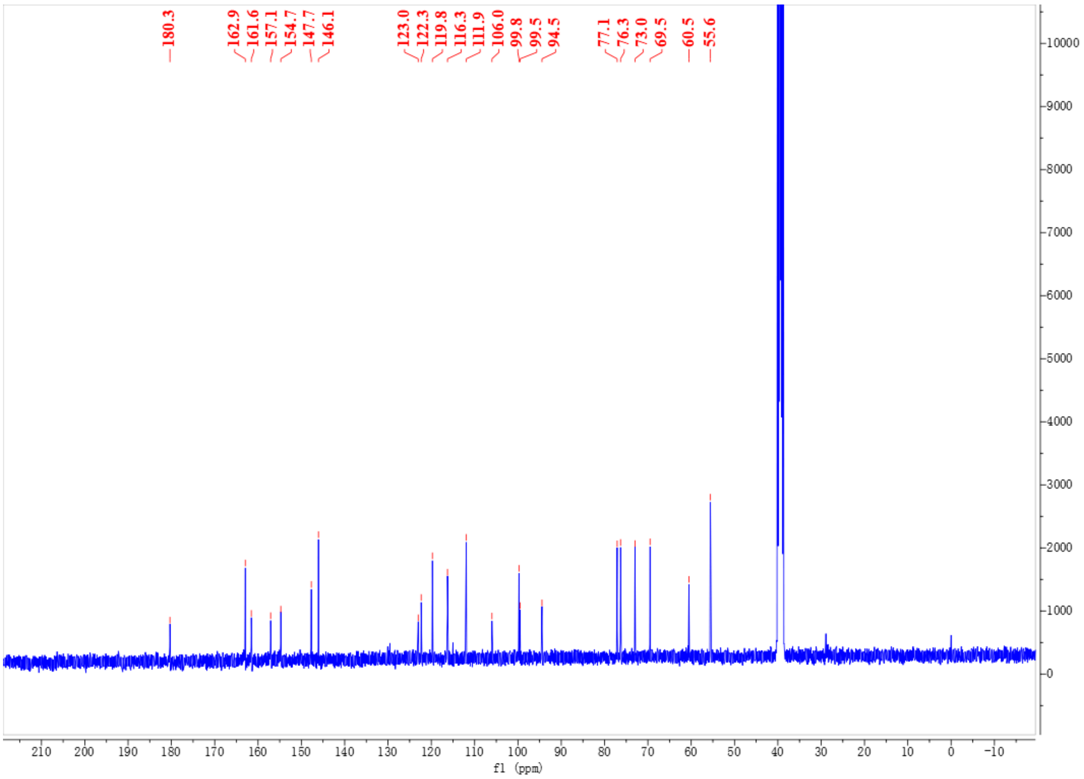

Figure S98. The ^{13}C NMR spectrum of compound **11** (151 MHz, DMSO-d_6).

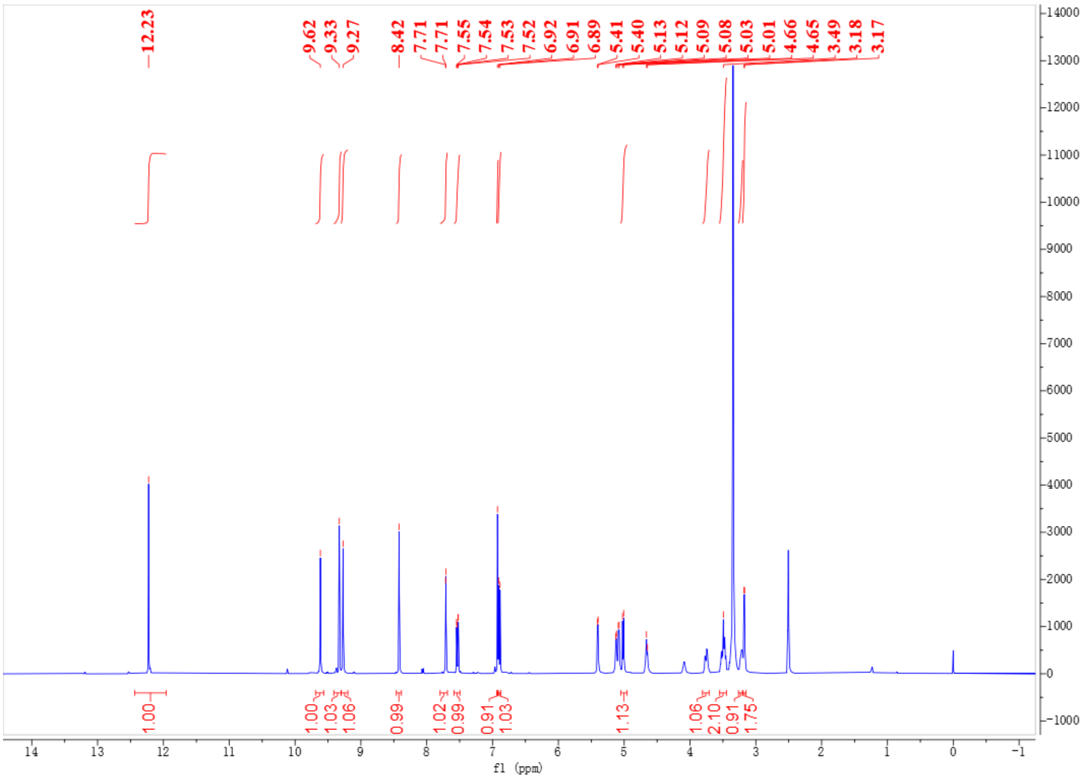

Figure S99. The ^{1}H NMR spectrum of compound **12** (600 MHz, DMSO-d_6).

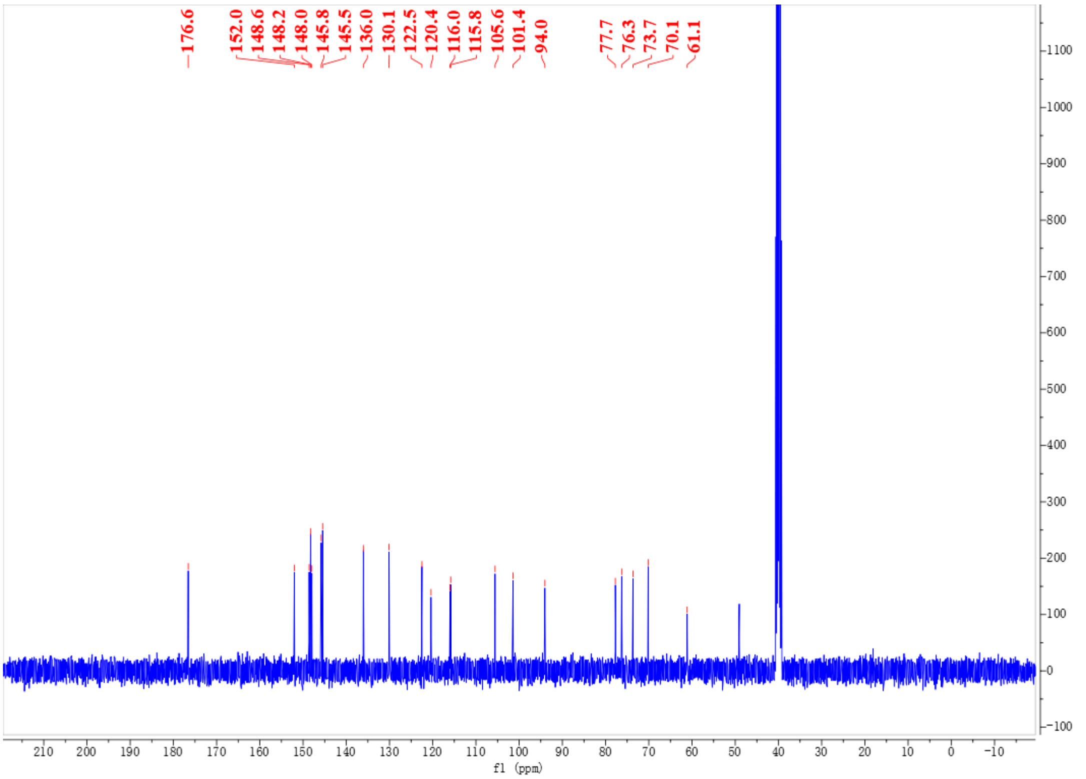

Figure S100. The ^{13}C NMR spectrum of compound **12** (151 MHz, DMSO-d_6).

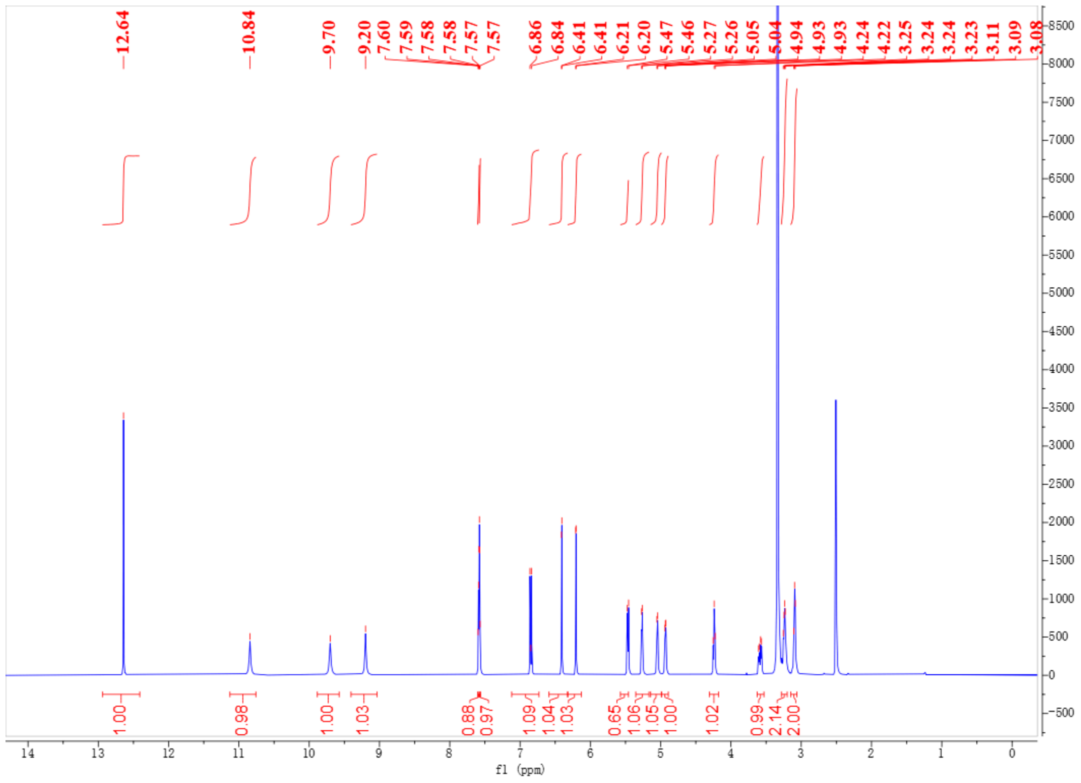

Figure S101. The ^{1}H NMR spectrum of compound **13** (600 MHz, DMSO-d_6).

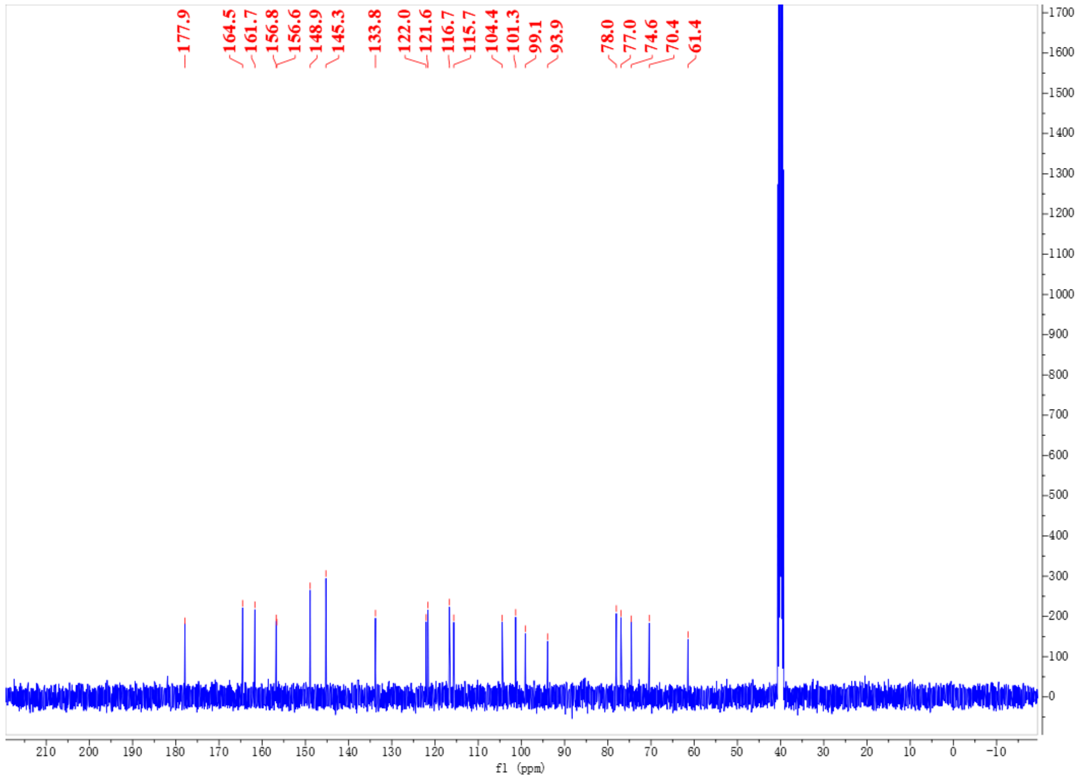

Figure S102. The ^{13}C NMR spectrum of compound **13** (151 MHz, DMSO-d_6).

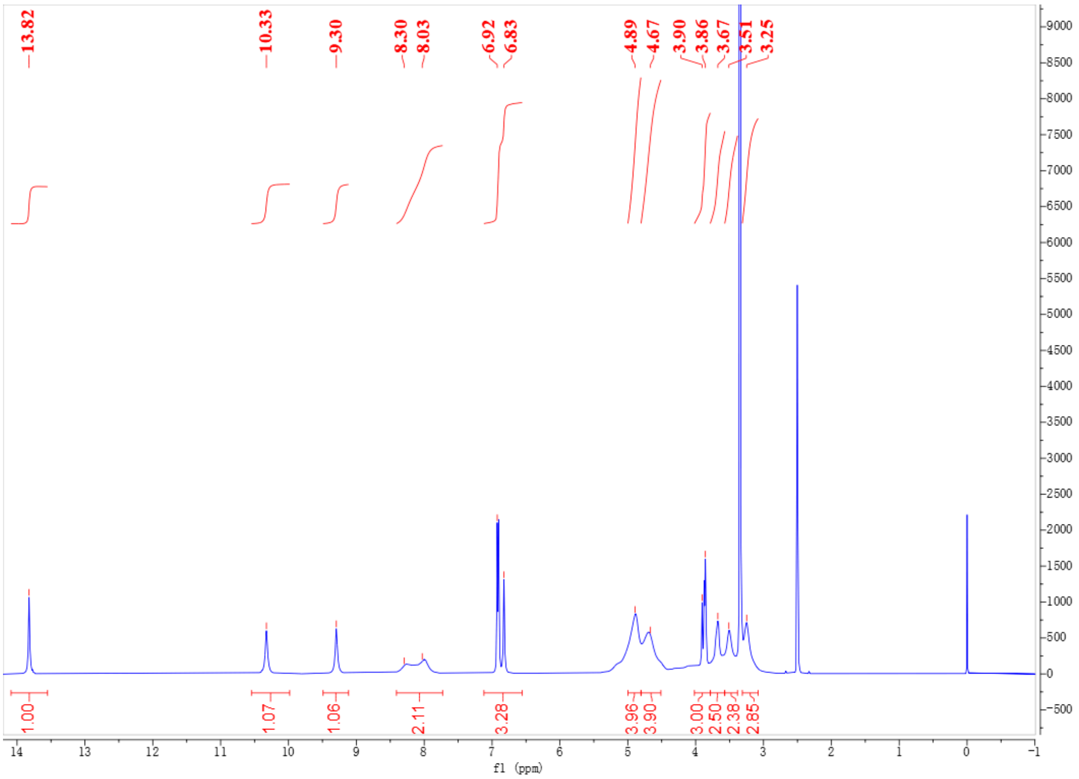

Figure S103. The ^{1}H NMR spectrum of compound **14** (600 MHz, DMSO-d_6).

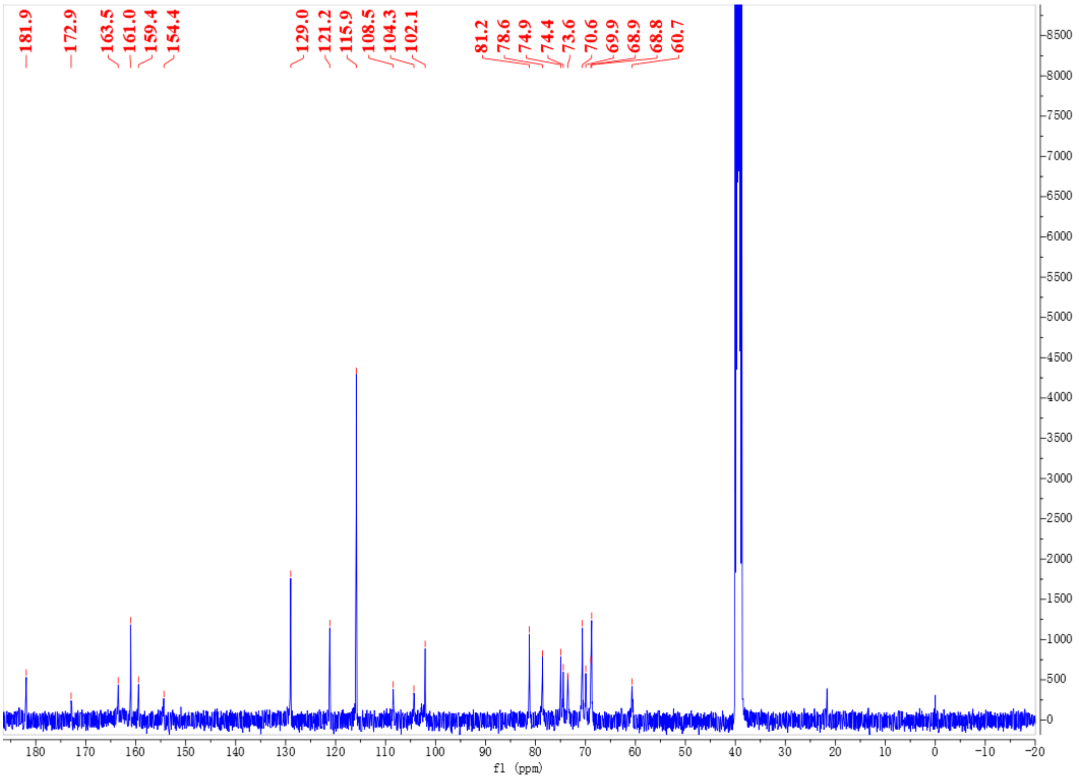

Figure S104. The ^{13}C NMR spectrum of compound **14** (151 MHz, DMSO-d_6).

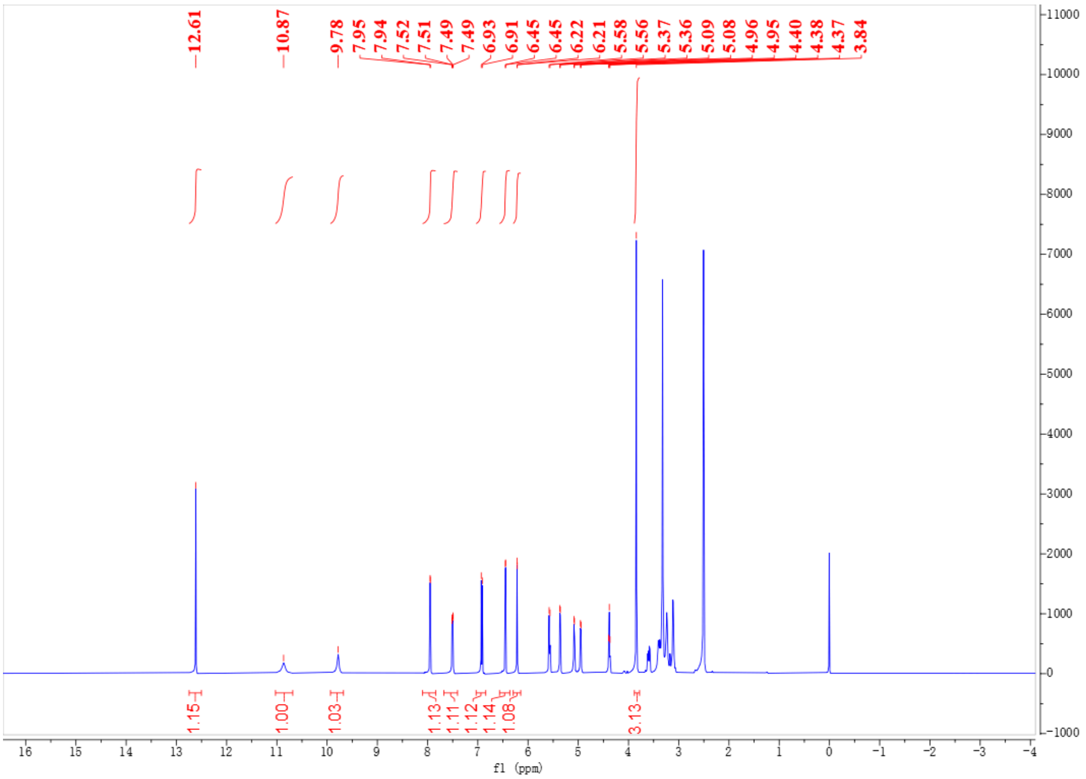

Figure S105. The ^{1}H NMR spectrum of compound **15** (600 MHz, DMSO-d_6).

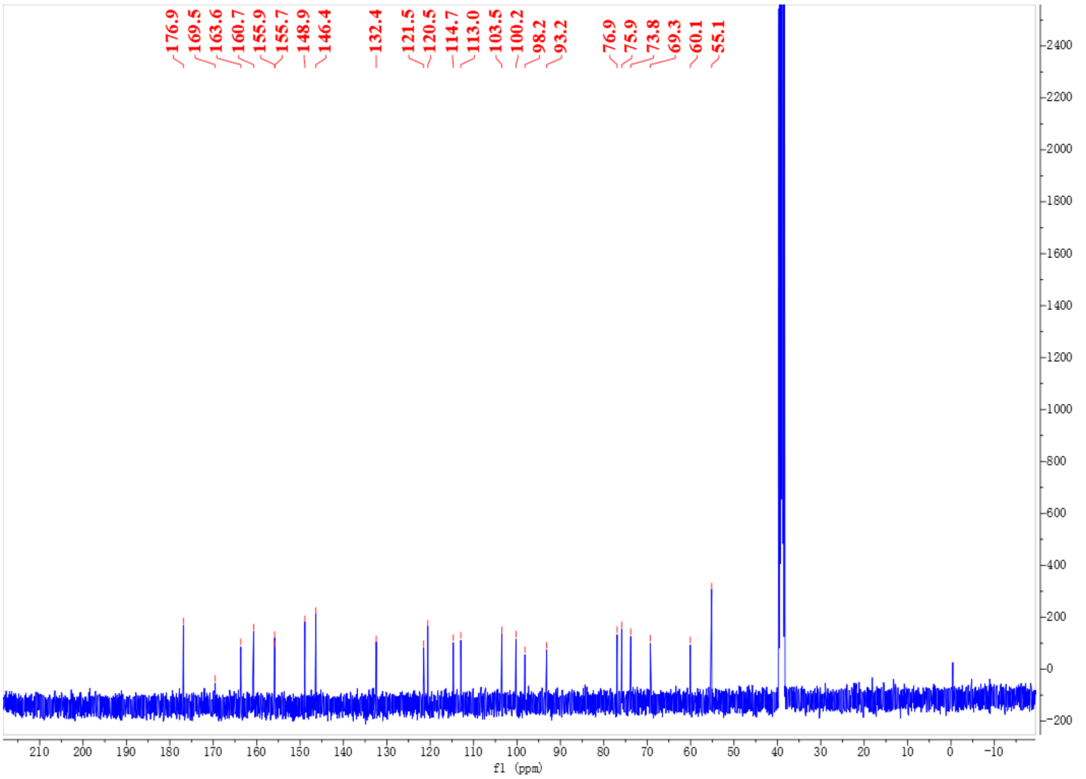

Figure S106. The ^{13}C NMR spectrum of compound **15** (151 MHz, DMSO-d_6).

12. Molecular docking results of compounds **1−7** and **9−10** with α-Glycosidase.

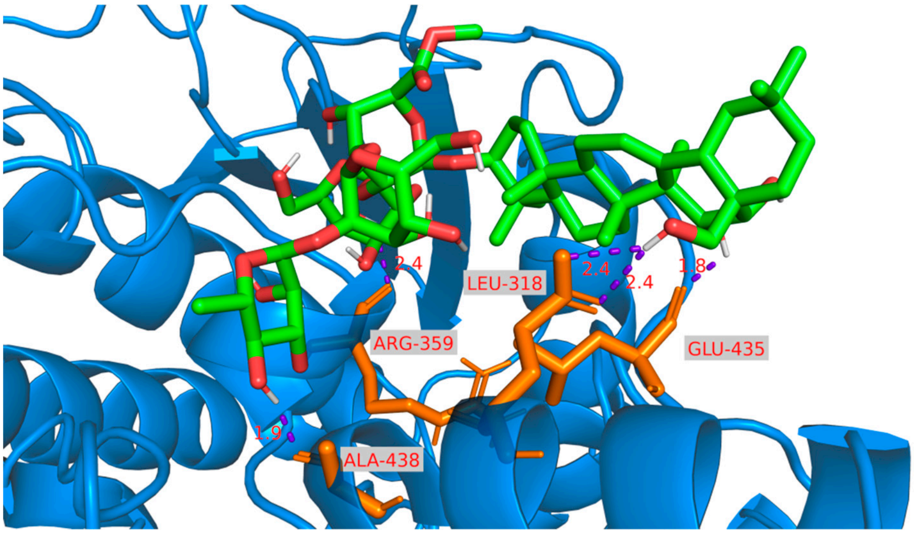

Figure S107. Molecular docking of compound **1**.

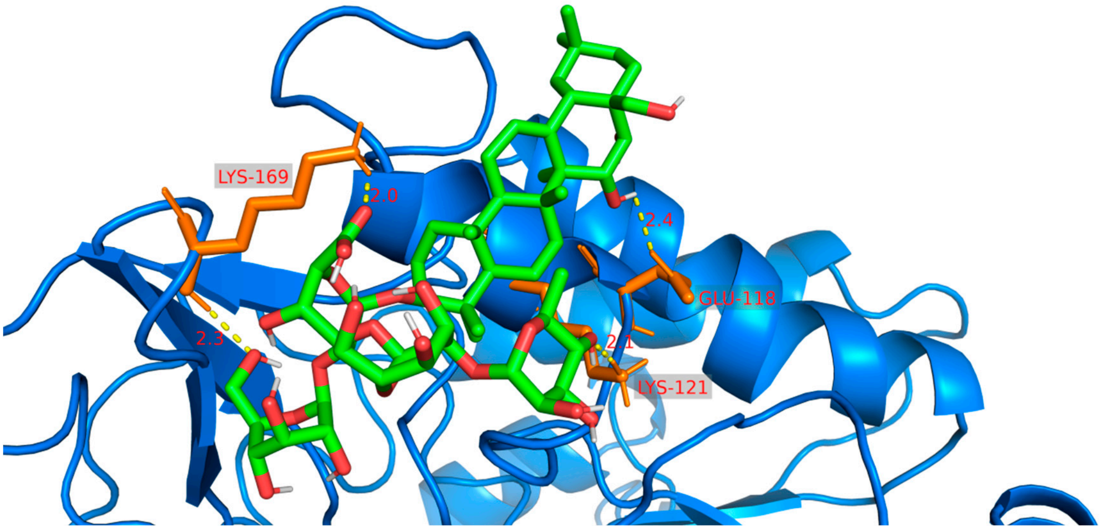

Figure S108. Molecular docking of compound **2**.

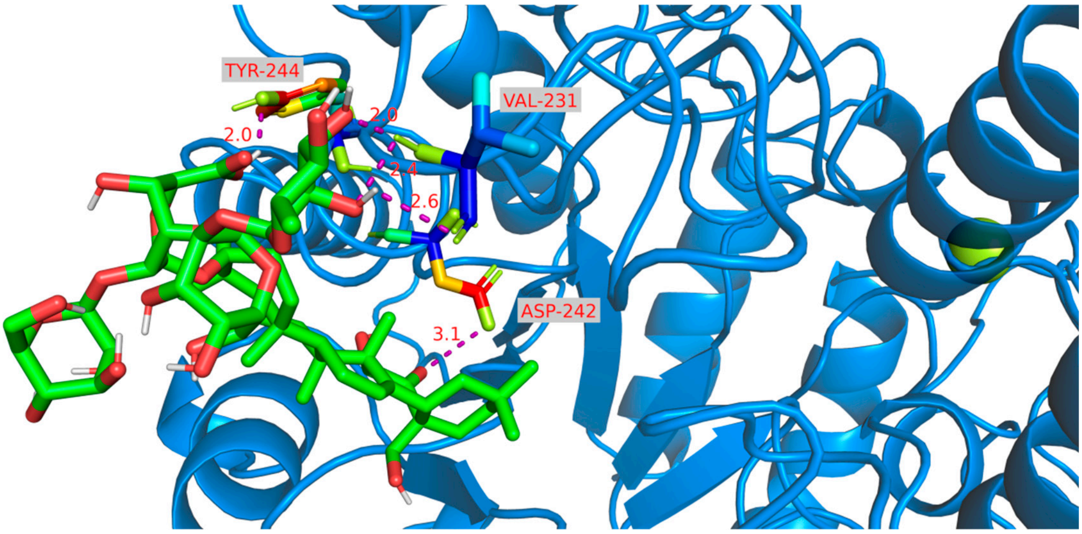

Figure S109. Molecular docking of compound **3**.

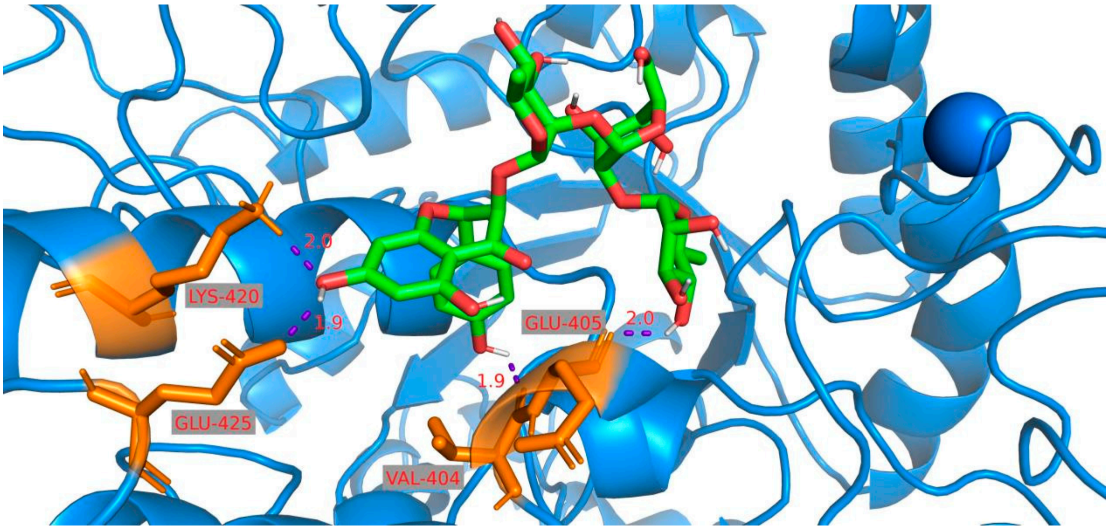

Figure S110. Molecular docking of compound **4**.

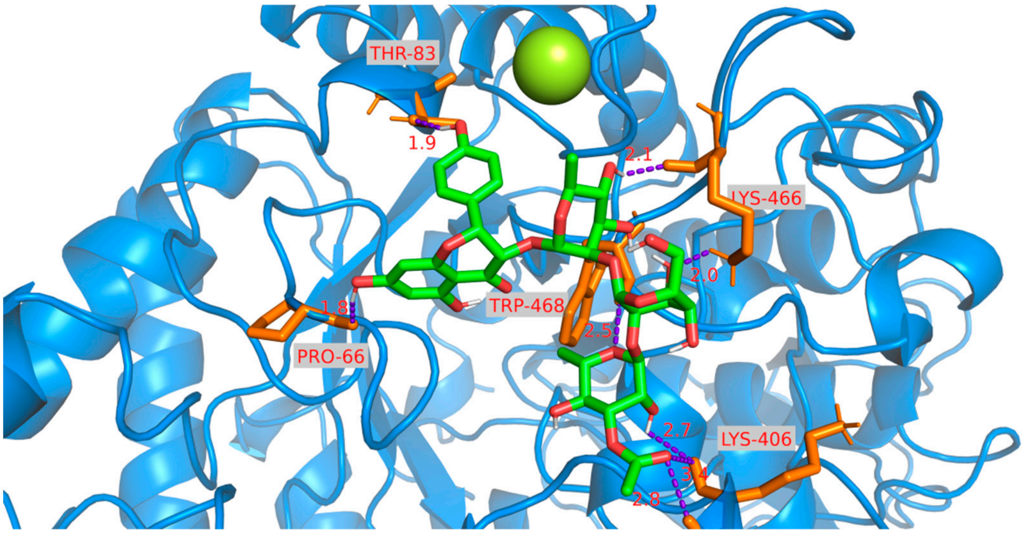

Figure S111. Molecular docking of compound **5**.

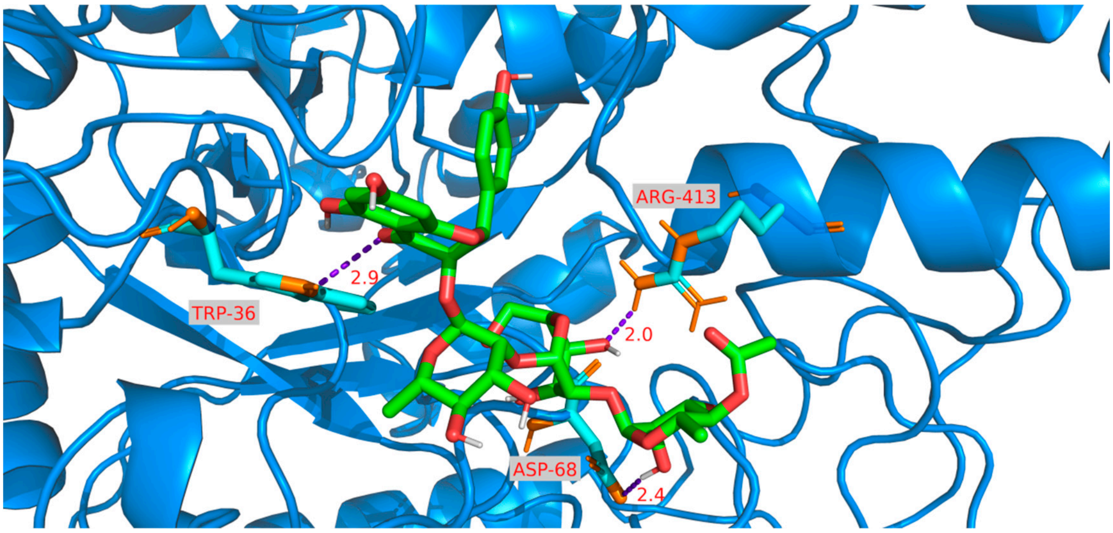

Figure S112. Molecular docking of compound **6**.

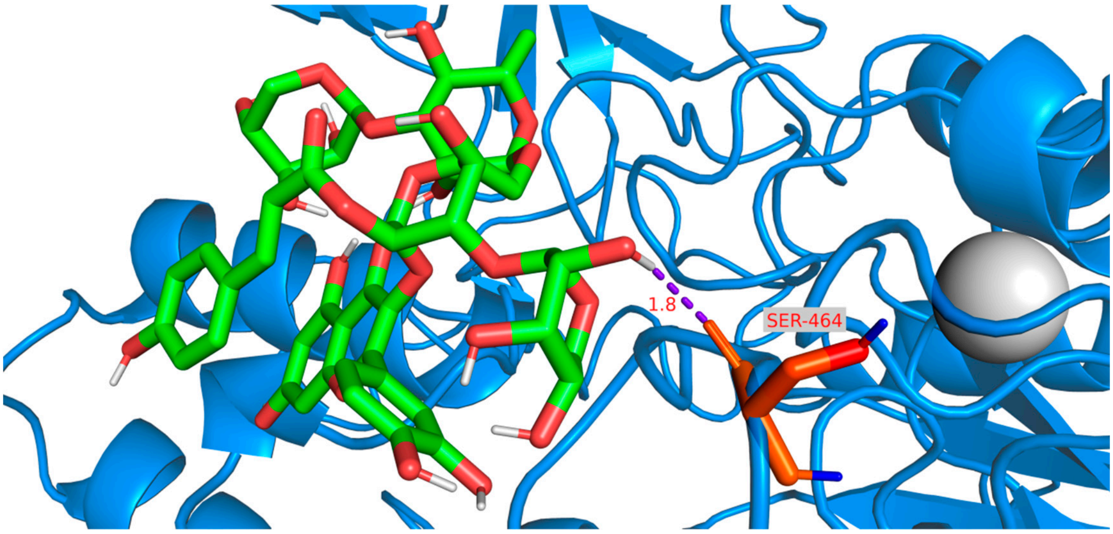

Figure S113. Molecular docking of compound **7**.

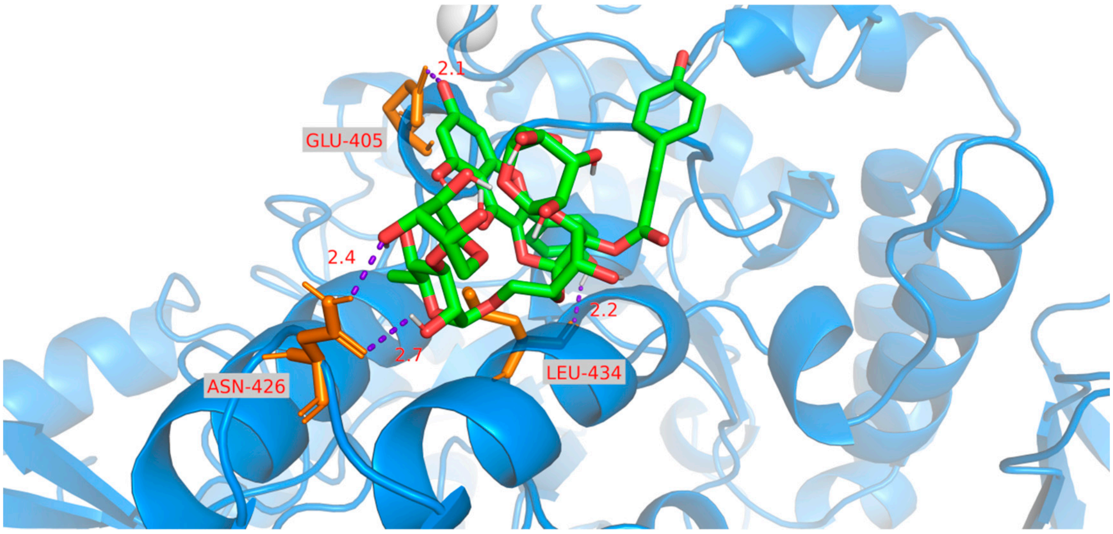

Figure S114. Molecular docking of compound **9**.

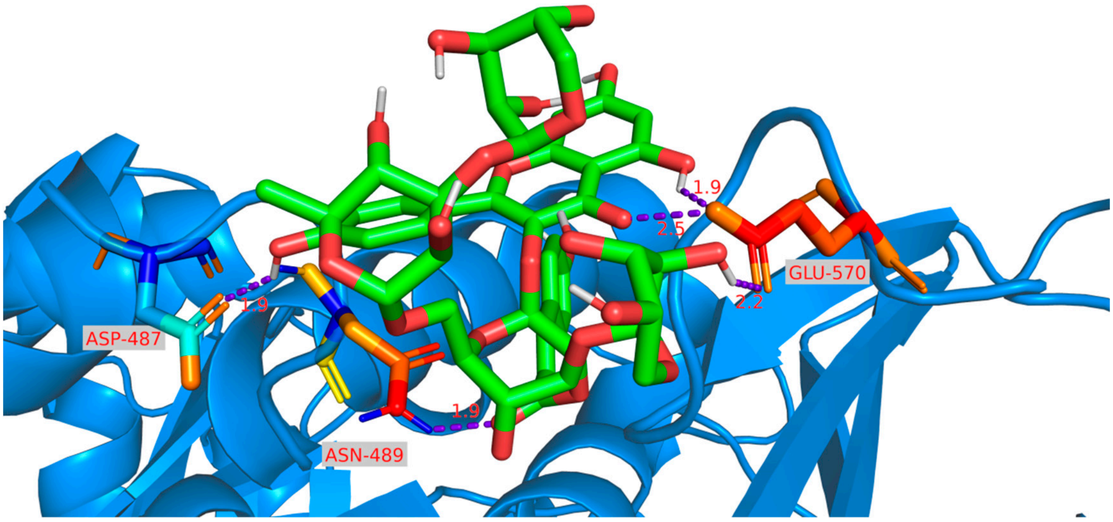

Figure S115. Molecular docking of compound **10**.

Table S1 Molecular docking results of compounds **1–10** with *α*-glucosidase

Compounds	Binding energy (kcal/mol)	Number of hydrogen bonds	H-bond length	Amino acid residue
**1**	-0.93	5	1.8、1.9、2.4、2.4、2.4	LEU-318、ALA-438、ARG-359、GLU-435
**2**	-0.68	4	2.0、2.1、2.3、2.4	LYS-169、LYS-121、GLU-118
**3**	-1.21	5	2.0、2.0、2.4、2.6、3.1	TYR-244、VAL-231、ASP-242
**4**	-2.32	4	1.9、1.9、2.0、2.0	GLU-425、VAL-404、LYS-420、GLU-405
**5**	-3.17	8	1.8、1.9、2.0、2.1、2.5、2.7、2.8、3.4	PRO-66、THR-83、LYS-466、TRP-468、LYS-406、GLU-405
**6**	-2.44	3	2.0、2.4、2.9	ARG-413、ASP-68、TRP-36
**7**	-1.52	1	1.8	SER-464
**8**	-5.48	5	2.9、2.9、3.1、3.2、3.5	ASP-307、PRO-320、GLU-322、THR-310
**9**	-2.68	4	2.1、2.2、24、2.7	GLU-405、LEU-434、ASN-426
**10**	-2.04	5	1.9、1.9、1.9、2.2、2.5	ASP-487、ASN-489、GLU-570
